# Establishing analytical validity of BeadChip array genotype data by comparison to whole-genome sequence and standard benchmark datasets

**DOI:** 10.1186/s12920-022-01199-8

**Published:** 2022-03-14

**Authors:** Praveen F. Cherukuri, Melissa M. Soe, David E. Condon, Shubhi Bartaria, Kaitlynn Meis, Shaopeng Gu, Frederick G. Frost, Lindsay M. Fricke, Krzysztof P. Lubieniecki, Joanna M. Lubieniecka, Robert E. Pyatt, Catherine Hajek, Cornelius F. Boerkoel, Lynn Carmichael

**Affiliations:** 1 Imagenetics, Sanford Health, 1410 W 25th St. Room #302, Sioux Falls, SD 57105 USA; 2 Sanford School of Medicine, University of South Dakota, Sioux Falls, SD USA; 3 Sanford Research Center, Sioux Falls, SD USA

**Keywords:** Clinical genotyping, Genotyping error, Analytical validation

## Abstract

**Background:**

Clinical use of genotype data requires high positive predictive value (PPV) and thorough understanding of the genotyping platform characteristics. BeadChip arrays, such as the Global Screening Array (GSA), potentially offer a high-throughput, low-cost clinical screen for known variants. We hypothesize that quality assessment and comparison to whole-genome sequence and benchmark data establish the analytical validity of GSA genotyping.

**Methods:**

To test this hypothesis, we selected 263 samples from Coriell, generated GSA genotypes in triplicate, generated whole genome sequence (rWGS) genotypes, assessed the quality of each set of genotypes, and compared each set of genotypes to each other and to the 1000 Genomes Phase 3 (1KG) genotypes, a performance benchmark. For 59 genes (MAP59), we also performed theoretical and empirical evaluation of variants deemed medically actionable predispositions.

**Results:**

Quality analyses detected sample contamination and increased assay failure along the chip margins. Comparison to benchmark data demonstrated that > 82% of the GSA assays had a PPV of 1. GSA assays targeting transitions, genomic regions of high complexity, and common variants performed better than those targeting transversions, regions of low complexity, and rare variants. Comparison of GSA data to rWGS and 1KG data showed > 99% performance across all measured parameters. Consistent with predictions from prior studies, the GSA detection of variation within the MAP59 genes was 3/261.

**Conclusion:**

We establish the analytical validity of GSA assays using quality analytics and comparison to benchmark and rWGS data. GSA assays meet the standards of a clinical screen although assays interrogating rare variants, transversions, and variants within low-complexity regions require careful evaluation.

**Supplementary Information:**

The online version contains supplementary material available at 10.1186/s12920-022-01199-8.

## Background

Clinical genotyping requires assays with high positive predictive value (PPV) and minimal error [1]. The impact of genotyping error has been observed for variant association tests [2], sibling-pair analyses [3], and variant and genotype interpretation [4]. Genotyping errors occur when the observed genotype does not correspond to an individual’s true genotype [5]. Such errors arise from multiple factors including, but not limited to, biases in modeling algorithms [6], sample and technical batch effects [7], paralogous genomic regions [8], sample contamination [9], allele frequency differences on genotyping platforms [10], and DNA sample quality [11].

Several methods have been developed to detect and minimize genotyping errors. These include the quality control (QC) metrics of genotype call rate [12, 13] and sample contamination detection [14]. Additional methods include assessing departure from Hardy–Weinberg Equilibrium (HWE) [15–17], information content for each chromosome before and after removal of SNPs with high linkage disequilibrium (LD) [18], likelihood of error [19], departure from expected Mendelian inheritance [4], and pedigree information [20].

QC of genotype data minimizes the likelihood of errors [11, 21, 22]. Estimating true genotypes and detecting errors require well-characterized benchmark datasets such as those described for bioinformatic genotyping pipelines [23], quality control algorithms [24], and sequencing platforms [25–27]. Additionally, theoretical benchmark datasets are needed for analysis of genotype data and estimating genotyping error [28]. Compared to NGS [26, 29, 30], genotyping via DNA hybridization has distinct, well described genotyping and platform biases [10, 31, 32].

Clinical genotyping using DNA hybridization, e.g., the Global Screening Array (GSA), requires a comprehensive analytical framework to detect and limit error. Based on current research methodologies, we propose analytical validation of GSA genotyping by assessment of quality metrics and by comparison to truth sets: those of the 1000 Genomes Phase 3 (1KG), the National Institute of Standards and Technology (NIST), and the Genome in a Bottle Consortium (GiAB). To test this, we selected 263 Coriell DNA samples (Additional file 1: Table S1) and, for each sample, generated whole genome sequence (rWGS) at > 37× read depth and GSA genotypes in triplicate. These data were compared to each other and to the corresponding publicly available truth sets. Additionally, we characterized each GSA assay performance and biases by stratifying GSA assays according to allele frequency, nucleotide variant class, low-complexity regions, medically actionable variants, and other genomic features.

## Methods

### Aim and design of study

**Fig. 1:**
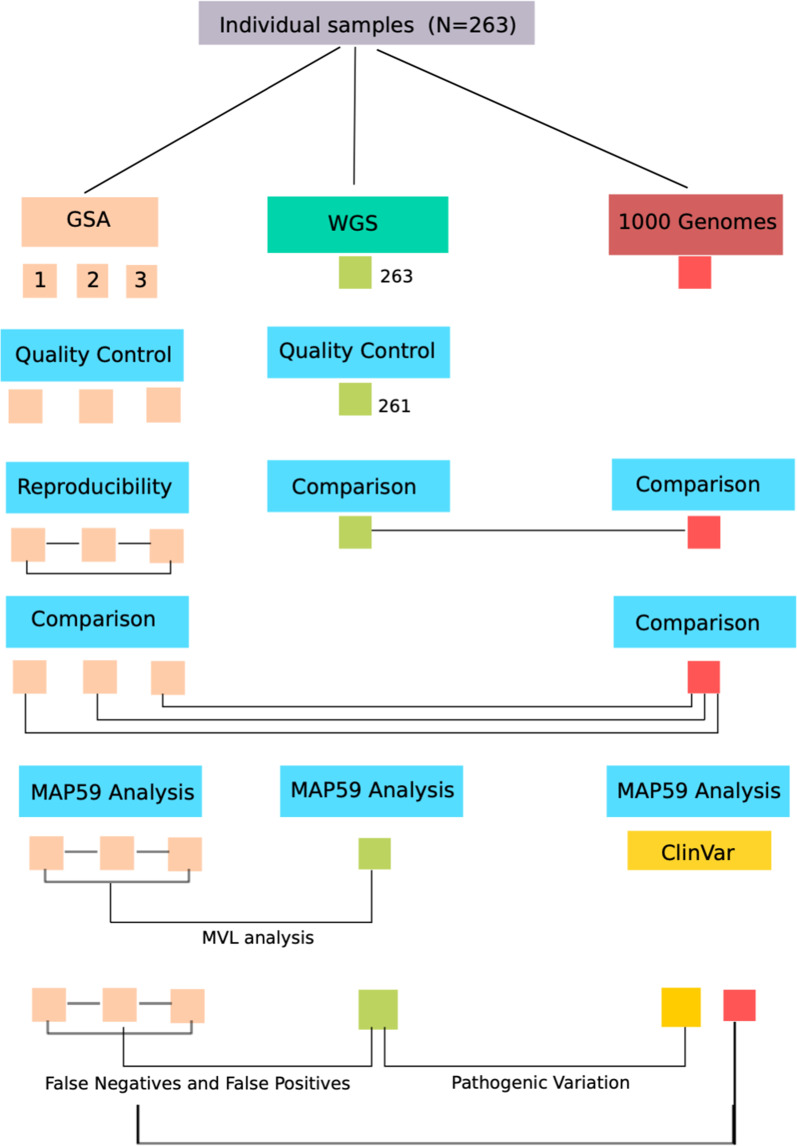
A flow-diagram showing the analytical validation framework for detecting and limiting genotyping error in BeadChip array data

This study defines an analytical validation framework for detecting and limiting genotyping error in GSA data (Fig. 1). To minimize platform specific genotyping biases, internally generated genotype data from independent platforms were paired and compared with publicly available genotype datasets.

### Samples and datasets

To generate a reference genotype cluster file for the GSA, 664 DNA samples were purchased from the Coriell Institute for Medical Research, Camden, NJ (https://www.coriell.org) and 460 samples were selected from the Sanford Biobank. Individuals with biobank samples were enrolled in protocol number 03-11-061 approved by the Sanford Research Institutional Review Board. These samples were selected to cover different ethnicities (14 Coriell diversity panels, Additional file 1: Table S2) and the technical variability of the DNA extraction methods (460 samples from the Sanford Biobank). To capture the technical variability of the Infinium® HTS Assay protocol (Illumina Inc.), all samples were genotyped in triplicate (by different technicians, robot-instrument configurations, reagent lots, and days) using the Infinium Global Screening Array-24 v.1.0 BeadChip. The resulting data were loaded into GenomeStudio v2.0.2 and used to generate the genotype cluster files per manufacturer recommendations (https://www.illumina.com/Documents/products/technotes/technote_infinium_genotyping_data_analysis.pdf). Of the 1104 samples used in cluster file generation 72 were also included among the 263 samples used to define analytical validity (Supplementary Sects. “Methods” and “Aim and design of study”). Two hundred sixty-three DNA samples from Coriell were selected as representative of individuals from the 1000 Genomes Project Consortium (*n* = 258) and from the Genome in a Bottle Consortium (GiAB) [33] (*n* = 5) (Additional file 1: Table S1). Additionally, they were selected to assess assays genotyping alleles with ≥ 1% minor allele frequency (MAF) in the general population (Additional file 1 Table S3). These 263 DNA samples were resequenced with whole genome sequencing (rWGS) and genotyped in triplicate (263 × 3) with the GSA. These data were compared to 1KG and to publicly available Whole Genome Sequence (pWGS) data (1KG phase 3; downloaded: June 2018). This defined 4 genotype datasets for the 263 samples: (i) triplicate GSA genotypes (ii) pWGS, (iii) rWGS, and (iv) 1KG (Additional file 1: Table S4, Fig. S2, Sect. S3). All analyses including mapping, alignment, and genotyping were performed using HumanG1Kv37 (Genome Reference Consortium Human build 37).

### Data generation

#### Illumina Infinium GSA

Illumina’s GSA—24 v1.0 BeadChips (24-sample format) were processed following the standard Infinium High-throughput Screening (HTS) protocol using the Freedom EVO® platform (Tecan) and AutoLoader 2.x (Illumina, Inc.). Raw intensity data for each bead on a BeadChip were collected using the iScan® System (Illumina, Inc.) and saved as intensity files. The intensity files were converted to genotypes by the AutoConvert feature in the iScan Control software using the GenCall algorithm and the Illumina GSA manifest file. The normalized genotype data were saved as binary files and used as input for GenomeStudio v2.0.2 to generate preliminary Quality Control (QC) parameters (CallRate, p10GC), B-allele frequency files, log-likelihood files, and Variant Call Format (VCF) files (https://samtools.github.io/hts-specs/VCFv4.1.pdf). Genotypes were called relative to HumanG1Kv37 using *gtc_to_vcf.py* (v1.1.1) (https://github.com/Illumina/GTCtoVCF). Alleles matching the reference allele were encoded as ‘0’, first alternate allele as ‘1’, second alternate allele as ‘2’, and third alternate allele as ‘3’. The allelic combinations for genotypes were encoded as 0/0, 0/1, 1/1, 0/2, etc. for a total of 10 possible genotypes. All possible genotypes and their comparisons are shown in Table 1.

**Table 1:**
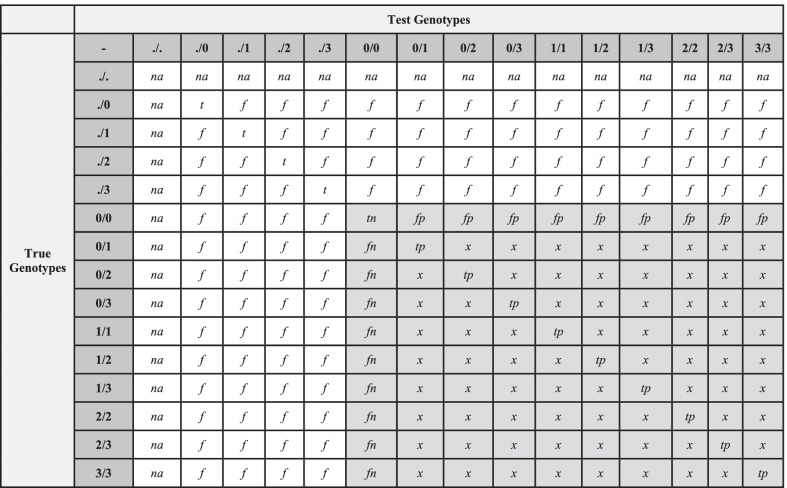
Definition of genotypes and comparison of test and truth sets to each other

*tp* true positive, *fp* false positive, *tn* true negative, *fn* false negative, *x* other discordant genotypes, *na* no data, *f* false genotype, *t* true genotype

#### Whole genome sequencing (rWGS)

The 263 DNA validation samples purchased from Coriell (Additional file 1: Table S1) were sequenced using the Illumina HiSeqX by Genome.One (Sydney, Australia). rWGS produced an average of 731 million 150 bp paired-end reads to give an average of 37× depth of coverage (range: 32× –42×) across HumanG1Kv37 (Additional file 1: Tables S4, S5, S6 and S7). Fastq were transferred to GenomeNext (http://genomenext.com) and processed using the Churchill pipeline [34]. QC data and genotypes were saved as VCF, genomic VCF (gVCF), and binary alignment (BAM) files. In total, 22.3 TB of rWGS data were archived on Amazon Web Services Storage 3 (AWS S3). 262 rWGS BAM files (all sequence data) were submitted to NCBI SRA database and are publicly available (https://www.ncbi.nlm.nih.gov/sra/; BioProject: PRJNA792997. Additional file 1: Sect. S8).

### Data processing

#### GSA quality control (QC)

##### Laboratory QC

Genotype clusters for the variants used for clinical reporting were manually curated to ensure accurate variant calling. Other variants were automatically curated using Illumina-recommended filters (https://www.illumina.com/Documents/products/technotes/technote_infinium_genotyping_data_analysis.pdf). Using the data of DNA samples from 1104 individuals run on the GSA in triplicate, the cluster file analyses of each GSA assay found that 610,771 (92%) assays passed and 50,355 (8%) assays failed clustering quality control. Those that failed were excluded and marked as no-calls (./.) in the VCF files.

##### Bioinformatics QC

**Fig. 2:**
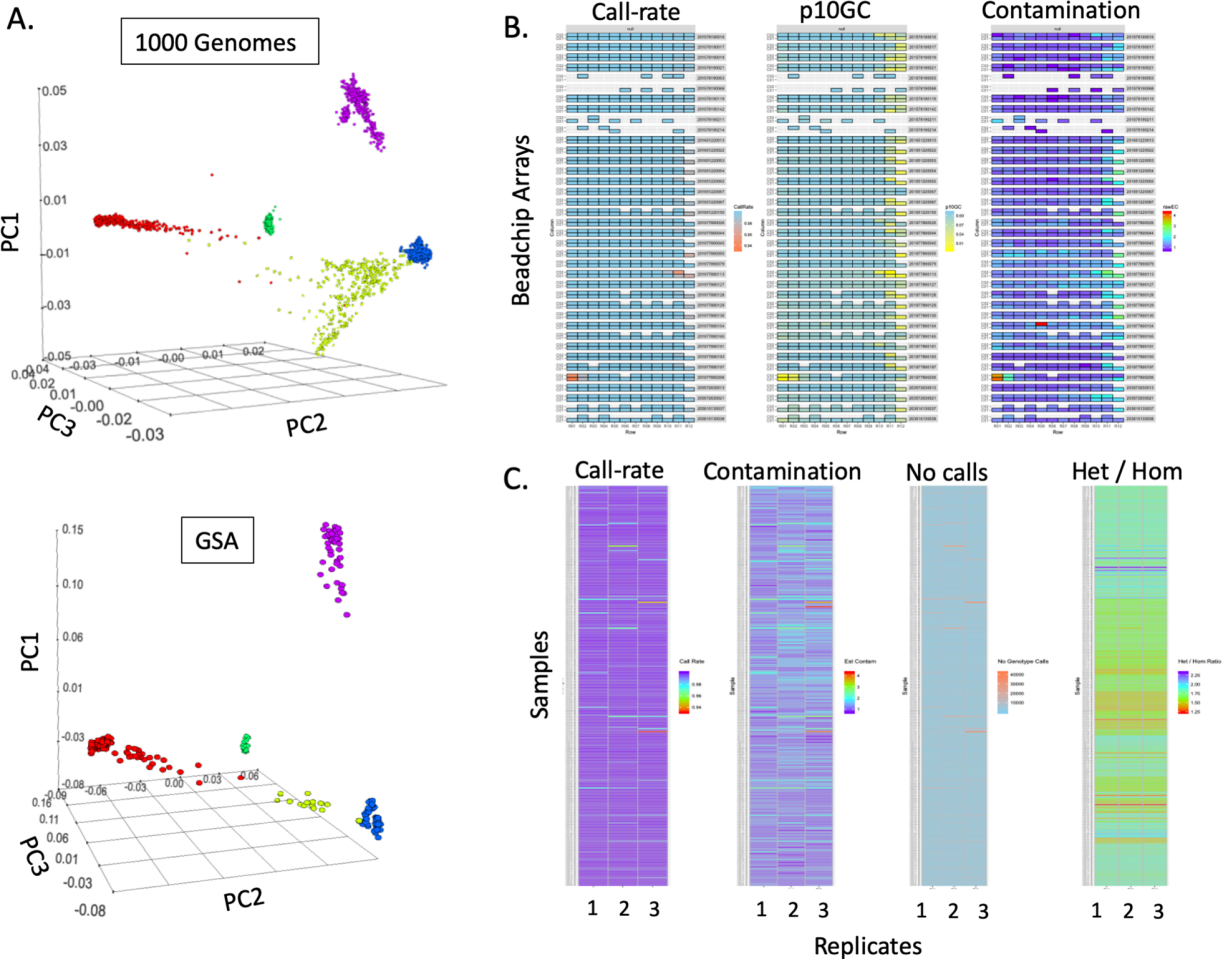
Aggregate quality control analysis of the GSA data. **A** Principal Component Analysis (PCA) plots of 1KG data and GSA genotype data. red: African (AFR), yellow-green: Admixed Americans (AMR), dark-green: East Asian (EAS), blue: European (EUR), purple: South Asian (SAS). **B** Heatmaps of BeadChip array quality control analysis of call-rate (left), p10GC (middle), and estimated DNA contamination (right). Color gradient scales for the three panels are as follows: call-rate (orange < 0.94–blue > 0.99), p10GC (yellow < 0.50–blue > 0.60) and estimated DNA contamination (rainbow gradient: purple ~ 1%, blue ~ 2%, green ~ 3%, orange/red ~ >4%). **C** Heatmaps of reproducibility quality control analysis using replicate data as measured by call rate, estimated DNA contamination, number of assays with no genotype calls, and heterozygote to homozygote ratio. Color gradient scales for these four heatmaps are as follows: No genotype calls (blue < 166,000–orange > 400,000), and rainbow gradient for call rate (purple > 0.99–red < 0.94), estimated DNA contamination (purple < 1%–red > 4%), and heterozygote/homozygote ratio (purple > 2.25–red < 1.25), respectively

The GSA data (*n* = 263 × 3 replicates) were stratified by the BeadChip identifier and the sample location on the BeadChip (row, column) and grouped by sample replicate. For each sample, the 610,771 assays that passed cluster file QC were used to evaluate the following parameters: (i) genotype call rate, (ii) p10GC, and (iii) estimated sample contamination. Sample contamination was estimated according to the method of Jun, G. and colleagues (Jun et al. 2012) (Methodology in Additional file 1: Sect. S5). Aggregate QC analyses are shown in Fig. 2. Replicated GSA data for 262 samples (in triplicate) were deposited to the dbSNP database (Additional file 1: Sect. S8).

### Data comparisons

#### Principal component analysis

Principal component analysis (PCA) was used to test for intact super-population structure as a corollary for absence of batch and technical artifacts in the genotyping datasets. PCA structure derived from GSA data was compared to the super-population structure derived from 1KG data.

### Whole genome sequence data quality control (QC)

#### Bioinformatics QC

For bioinformatics quality control of rWGS data (*n *= 263), central tendency and anomalous outlier data points were assessed for (i) total processed reads, (ii) discordant reads, (iii) mapq0 reads, (iv) unmapped reads, (v) mapped reads, and (vi) average depth of sequencing (Additional file 1: Tables S4 and S5). On average > 95% of processed reads per sample (731,227,993/767,540,183 reads) mapped to the reference sequence. Because the concordance of two rWGS datasets (HG00111 and HG00257) with the 1KG data were 0.870 and 0.622, they were dropped from our GSA analyses leaving a total of 261 samples in the rWGS dataset. Variation data for 260 samples (SNVs and short indels ≥ 20× coverage and a Phred score ≥ 30) were submitted to the dbSNP database (Additional file 1: Sect. S8).

### Performance metrics

#### Genotype concordance, sensitivity, specificity and positive predictive value (PPV)

GSA and rWGS genotypes were compared to each other and to 1KG genotypes using the following performance metrics: (i) genotype concordance (C), (ii) sensitivity (S), (iii) specificity (P), and (iv) positive predictive value (PPV). We used the following definitions of genotype classification to label genotypes as positive [true positive (*tp*), false positive (*fp*)], negative [true negative (*tn*), false negative (*fn*)], or discordant (*x*) (Table 1):

1 $$a = \sum tp$$

2 $$b = \sum fp$$

3 $$c = \sum tn$$

4 $$d = \sum fn$$

5 $$z = \sum x$$

Given the above definitions of true/false positive and negative and discordant genotypes (Table 1), we computed the performance metrics as follows:

Genotype concordance (*C*)

6 $$C = \left( {\frac{a + c}{{a + b + c + d + z}}} \right)$$

Sensitivity (S)

7 $$S = \left( {\frac{a}{a + d}} \right)$$

Specificity (*P*)

8 $$P = \left( {\frac{c}{c + b}} \right)$$

Positive predictive value (PPV)

9 $${\text{PPV}} = \left( {\frac{a}{a + b}} \right)$$

### Classification of GSA assays

#### Variation type

GSA assays were stratified according to variant classes: single nucleotide variants (SNVs; 656,601), multi-allelic variants (MAVs; 616), deletions (DEL; 2799), and insertions (INS; 1110).

##### Nucleotide change class

By parsing the VCF files and cataloging the alternate nucleotide, SNVs were stratified by whether the nucleotide change was a transition or a transversion.

##### Allele frequency

SNVs were binned into 13 strata based on the alternate allele frequency reported in the 1KG VCF file (allele frequency × 100): (a) [0–0.1%], (b) (0.1–1%], (c) (1–5%], (d) (5–10%], (e) (10–20%], (f) (20–30%], (g) (30–40%], (h) (40–50%], (i) (50–60%], (j) (60–70%], (k) (70–80%], (l) (80–90%], and (m) (90–100%].

##### Genomic complexity of variation locus (low-complexity regions)

To categorize SNVs based on the genomic complexity of the GSA assay locus, we used the UCSC genome browser bed-file definitions to define simple-repeats, micro-satellite regions, and low-complexity regions. The SimpRep, Microsatellites, and RepeatMasker bedfiles were downloaded from the UCSC Genome Browser FTP site and intersected with the GSA manifest file. Across the HumanG1Kv37 reference sequence, there were 962,715 simple repeat, 41,573 microsatellite, and 5,298,131 RepeatMasker regions.

### GSA panels

#### Medically actionable predispositions (MAP) 59 gene panel

GSA assays targeting potentially disease-associated variants in MAP59 genes [35] were selected in a multistep process (Table 2). Firstly, GSA assays that interrogated positions within 1000-bases upstream and downstream of the transcript start and end in HumanG1Kv37 were selected for the RefSeq transcript chosen for each gene. Secondly, alleles were annotated with their respective ClinVar classifications, and those that had at least one classification of pathogenic or likely pathogenic were selected. Thirdly, these assays were curated by clinical and laboratory staff to define a managed variant list (MVL) of 1883 assays appropriate for clinical reporting.

**Table 2:** Selection process for GSA assays targeting genotypes considered medically actionable predispositions

GSA MAP59 subsets	Number of assays
GSA MAP59 (+ / − 1 kb)	6841
GSA MAP59 (*select:* “ClinVar” AND “Predicted Path”)	5075
GSA MAP59 (*select:* “ClinVar” AND “Predicted Path” AND “HGMD”)	3082
GSA MAP59 MVL (*select:* “ClinVar” AND “Predicted Path” AND “HGMD” AND “Curated”)	1883

*HGMD* Human Gene Mutation Database, *Path* pathogenic, *MVL* Managed Variant List

### Statistics and compute infrastructure

Statistical analyses and data visualization were performed using R (version 3.4.3). Data analysis was done on a Linux Operating System with the following configuration: x86_64, 32 CPUs, 2.8 GHz AMD Opteron Processor 6320. AWS EC2 instances were spun-up for large compute jobs. All NGS and GSA data were archived on AWS S3. In-house software and data processing code and scripts were written primarily in Perl, Ruby, awk, and bash.

## Results

### Data summary

DNA samples from 263 individuals were purchased from Coriell and genotyped in triplicate (*n *= 789) with the GSA. Genotypes and data for each replicate were saved to a VCF file. The GSA data were grouped and summarized as replicate datasets 1, 2, and 3. Of the 263 samples, 258 were present in the 1KG. Of the other 5 samples, 3 were from the Personal Genomes Project (PGP) [36], and 2 were from the NIGMS Human Genetic Cell Repository. The 263 × 3 data were compared with the 1KG data and with two WGS datasets, the resequenced WGS data (*n* = 261; rWGS = 37×) and the downloaded public WGS data (*n* = 24; pWGS = 51×) (Additional file 1: Sect. S3).

### Principal component analysis defines the same population structure in GSA data and 1KG data

Principal component analysis (PCA) on each replicate of autosomal GSA data identified 5 major super populations conserved across replicates. PCA of the 1KG autosomal genotype data from the same loci generated a similar population structure (Fig. 2A). This suggested that the GSA data did not have confounding technical factors skewing the PCA plot. To determine if fewer GSA genotypes were sufficient for this test, we randomly sub-sampled close to 10,000 genotypes; these recapitulated the population structure (Additional file 1: Sect. S4).

### GSA triplicate data analysis shows data reproducibility in the majority of samples and no detectable stochastic QC failure

**Fig. 3:**
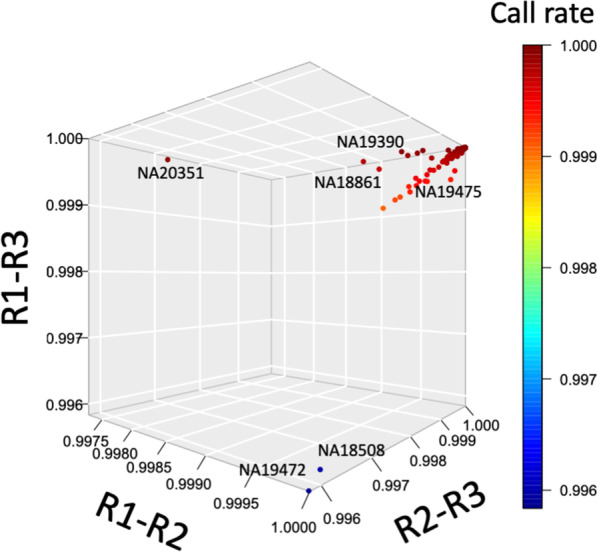
Three-dimensional scatterplot showing reproducibility of GSA call rate measured in three replicates for each Coriell sample (pairwise analysis of triplicate data). The data is plotted as correlation across triplicates for all measured GSA genotypes for a given DNA sample. Note that most samples had concordance greater than 0.999 between replicates suggesting high reproducibility. A few samples had off-diagonal points, i.e., those with poor call rates or reproducibility. The color rainbow gradient is from blue (< 0.996) to dark red (1.00)

Given that PCA did not detect major technical confounders within the GSA genotypes, we analyzed the 263 × 3 data for quality and reproducibility [10] (Table 3; Fig. 3). Data were stratified by BeadChip identifiers and sample location on the BeadChip (row, column). Additionally, samples were grouped by replicates, and each replicate sample was evaluated for (i) genotype call-rate (*n* = 610,771 assays), (ii) p10GC, and (iii) estimated DNA sample contamination (Additional file 1: Sect. S5). Aggregate quality control analysis showed a lower p10GC in higher numbered rows on the BeadChip (Fig. 2B); excluding contaminated samples, p10GC ranged from 0.56–0.61 (mean = 0.60,  *SD* = 0.0085) in row 1 and from 0.50–0.61 (mean = 0.55, *SD* = 0.03) in row 12. Over 99% (782/789) of samples had a call rate of > 0.98. 3 samples in the third replicate dataset were contaminated, and 2 of these 3 samples had a call rate < 0.98 (0.93 and 0.94, Fig. 2C).

**Table 3:** Summary of GSA triplicate data and average number of genotypes detected in all triplicate samples

	Replicate 1	Replicate 2	Replicate 3	All data
Total genotypes called	609,852 (± 1625)	609,723 (± 2548)	609,648 (± 3501)	609,741 (± 2668)
Missing genotypes	919 (± 1625)	1048 (± 2548)	1122 (± 3501)	1030 (± 2668)
Autosomal genotypes	599,666 (± 1619)	599,538 (± 2538)	599,467 (± 3459)	599,557 (± 2645)
Autosomal heterozygous genotypes	103,328 (± 4061)	103,221 (± 4063)	103,309 (± 4087)	103,286 (± 4066)
Autosomal homozygous alternate genotypesª	60,652 (± 3081)	60,643 (± 3098)	60,623 (± 3081)	60,639 (± 3083)

ªWe define the alternate genotype as a genotype different from homozygous reference sequence genotype

To test if call-rates were reproducible across replicates, we measured deviations from expectation and dispersion. The first approach, a Z-score method, computes the number of standard deviations a replicate sample call-rate is from the expected as defined by the global dataset average and standard deviation. The second approach computes the average call-rate of all replicates for a given sample and then computes variation around the average. Using the Z-score method, 7 samples had a Z-score ≤  − 4. With a more conservative cut-off (Z-score < −3), 11 samples deviated from expectation (Additional file 1: Sect. S6; Fig. S12). When analyzed relative to the BeadChip row and column, outlier Z-scores occurred for wells on the edge of the Illumina BeadChip—R12C01 or R11C01; the only exceptions were two contaminated samples that were in wells R01C01 and R01C02. Dispersion metrics calculated for call rates across each set of three replicates (Table 4) identified higher relative dispersion for the same samples detected by the Z-score method.

**Table 4:** Dispersion data paired with Z-score data

Sample	R1 call rate	R2 call rate	R3 call rate	Average	Z-score detected replicate	Dispersion (call rate)	Estimated contamination
NA20351	0.9987	0.9544	0.9989	0.984	R2	0.000447	2.75
NA19475	0.9793	0.9856	0.9961	0.987	R1	0.000048	2.5
NA19472	0.9986	0.9993	0.9451	0.981	R3	0.000659	3.75
NA19390	0.9918	0.9706	0.9989	0.987	R2	0.000146	2.5
NA18861	0.9991	0.9707	0.9817	0.984	R2	0.000139	2.5
NA18508	0.9987	0.9988	0.9318	0.976	R3	0.001021	4
HG03279	0.9793	0.9878	0.9967	0.988	R1	0.000051	2.5
NA19466	0.9989	0.9988	0.9971	0.998	–	0.000001	4.25 (R3)

Replicate pairwise concordance was calculated to assess the stochastic nature of sample genotyping quality and these were plotted as a 3D scatter-plot: [R1 vs. R2 (x-axis), R2 vs. R3 (y-axis), and R1 vs. R3 (z-axis)] (Fig. 3). The data along the diagonal of the cube are correlated data values across triplicates for all measured GSA genotypes for a given DNA sample. 260 of 263 samples in the triplicate dataset (262/263 R1 vs. R2; 260/263 R2 vs. R3; 261/263 R1 vs. R3) had concordance greater than 0.999 between replicates suggesting high reproducibility. Off-diagonal points, i.e., those with poor call rates (< 0.98) (Fig. 2B), were along the edge of Illumina chip or contaminated; we did not observe random occurrence of poor call rates.

### Grouping GSA assays by variation type shows that SNVs have > 0.99 performance relative to the benchmark dataset 1KG across all metrics

**Fig. 4:**
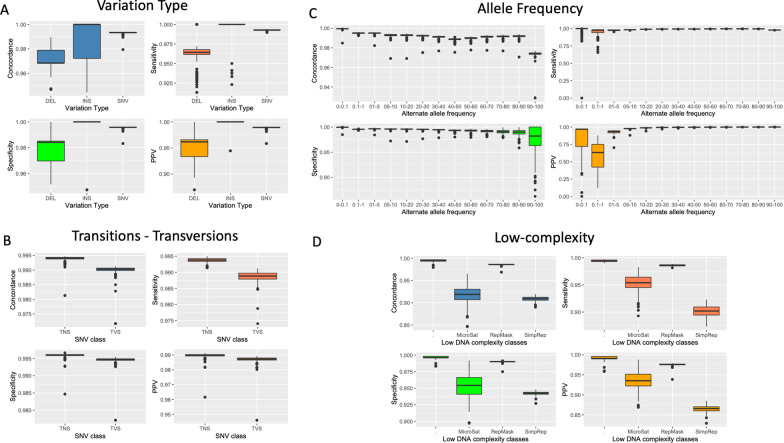
Boxplot analysis of the performance metrics of GSA vs 1KG benchmark dataset when assays are classified according to **A** variation type (deletion (DEL), insertion (INS), single nucleotide variant (SNV)), **B** type of single nucleotide change (transition (TNS), transversion (TVS)), (**C**) frequency of the alternate allele in the 1000 Genomes (1KG) data, and (**D**) interrogation of a low complexity genomic region (microsatellite region (MicroSat), RepeatMasker region (RepMask), or simple repeat (SimRep)). The performance metrics measured and plotted as boxplots for each class/panel are concordance (blue), sensitivity (coral), specificity (green) and positive predictive value (PPV) (orange)

Of the 263 samples with GSA data, 258 had corresponding 1KG genotype data for computing performance metrics of concordance, sensitivity, specificity, and PPV. Each GSA assay was grouped according to the type of nucleotide change assessed: (a) single nucleotide variant (SNV), (b) multi-allelic variant (MAV), (c) insertion, and (d) deletion (Table 5). SNVs accounted for 99.3% (656,601/661,126); 610,771 of these passed cluster file quality control, and 594,361 detected genotypes present in the 1KG. Among the MAV assays, 526 of 616 passed cluster file QC; however, because only 3 of these had genotypes present in the 1KG, we excluded MAVs from further analysis. Among insertion assays, 1044 of 1110 passed cluster file QC, and 36 of these had genotypes present in the 1KG. Among deletion assays 2677 of 2799 assays passed cluster file QC, and 95 of these had genotypes present in the1KG. Using the three replicate GSA genotype datasets, the performance metrics of SNV assays were > 0.99. In contrast, insertion assays had highly variable concordance with the 1KG, and deletion assays had poor performance metrics (Fig. 4A).

**Table 5:** Summary of GSA assays subgrouped by nucleotide variation type

Nucleotide variant type assay subsets	All GSA data	GSA pass manifest clusterfile QC	GSA pass manifest QC and present in 1KG Phase 3
Single nucleotide variants (SNVs)	656,601	606,524	594,230
Multi-allelic variants (MAVs)	616	526	3*
Insertions	1110	1044	36
Deletions	2799	2677	95
Total	661,126	610,771	594,361

*Poor overlap with 1KG MAVs and excluded from further analysis

### GSA assays for transitions perform better than do those for transversions

Classifying the GSA-detected SNVs as transitions (purine-to-purine OR pyrimidine-to-pyrimidine) or transversions (purine-to-pyrimidine or vice versa) identified 522,938 (79.6%) assays for transitions and 133,663 (20.4%) for transversions. 476,908 (91.2%) transition assays and 117,322 (87.8%) transversion assays passed cluster file QC and had genotypes present in the 1KG.

Assays for transitions performed better than those for transversions across all performance metrics. Overall concordance, sensitivity, specificity and positive predictive value for transitions versus transversions were 0.9985 vs. 0.9965, 0.9982 vs. 0.9965, 0.9994 vs. 0.9985 and 0.998 vs. 0.996, respectively (Fig. 4B). The assays for transversions between complementary nucleotides (i.e., A>T, T>A, C>G, G>C; Additional file 1: Sect. S7) had lower sensitivity (< 0.99) and lower cluster file QC pass rate (66–73%; Table 6) than did those for other transversions.

**Table 6:** Distribution of GSA (reference (Ref) to alternate (Alt) allele) SNV assays present in the 1KG Phase 3 data versus number of assays passing QC

	Ref\Alt	Purine	Purine	Pyrimidine	Pyrimidine
		A	G	C	T
Purine	A	–	101,994/111,493 (91%)	25,107/28,214 (89%)	1368/2065 (66%)
Purine	G	136,350/149,635 (91%)	–	2404/3280 (73%)	29,629/33,115 (89%)
Pyrimidine	C	30,130/33,566 (90%)	2339/3216 (73%)	–	136,392/149,801 (91%)
Pyrimidine	T	1362/2074 (66%)	24,983/28,133 (89%)	102,172/112,009 (91%)	–

### GSA assays for rare variants are harder to evaluate and confirm using benchmark datasets

Using the allele frequency in the 1KG as a surrogate for the general population variant allele frequency, we interrogated the effect of alternate allele (variant allele) frequency on the performance metrics. 643,012 GSA SNV assays were binned according to the alternate allele frequency extracted from the 1KG VCF file (allele frequency × 100): [0–0.1%], (0.1–1%], (1–5%], (5–10%], (10–20%], (20–30%], (30–40%], (40–50%], (50–60%], (60–70%], (70–80%], (80–90%], and (90–100%] (Table 7). On average the QC process removed 7–8% of assays from each bin. The bins [0–0.1%] and (90–100%] had 2% and 12% respectively removed (Table 7); this might reflect the small number of assays in these bins (17,830 and 4552, respectively). Consistent with previous publications (Ritchie et al. 2011), the average performance metrics for GSA assays passing cluster file QC in each bin showed that PPV and sensitivity suffered when the alternate allele frequency was < 5%, whereas specificity and concordance declined as the alternate allele frequency increased (Fig. 4C).

**Table 7:** Number of GSA assays and their relative percentages binned by alternate allele frequency in 1KG Phase 3 data

Alternate allele frequency bins (%)	All GSA assays and in 1KG	GSA pass QC and in 1KG	Percent assays that failed QC (%)
0–0.1	17,830	17,454	2
0.1–1	148,959	138,342	7
1–5	113,374	104,272	8
5–10	63,688	58,421	8
10–20	84,729	78,631	7
20–30	56,601	52,398	7
30–40	39,684	36,620	8
40–50	30,095	27,476	9
50–60	25,078	23,053	8
60–70	21,944	20,134	8
70–80	20,866	19,210	8
80–90	15,612	14,312	8
90–100	4552	4023	12
Total	643,012	594,346	8

**Fig. 5:**
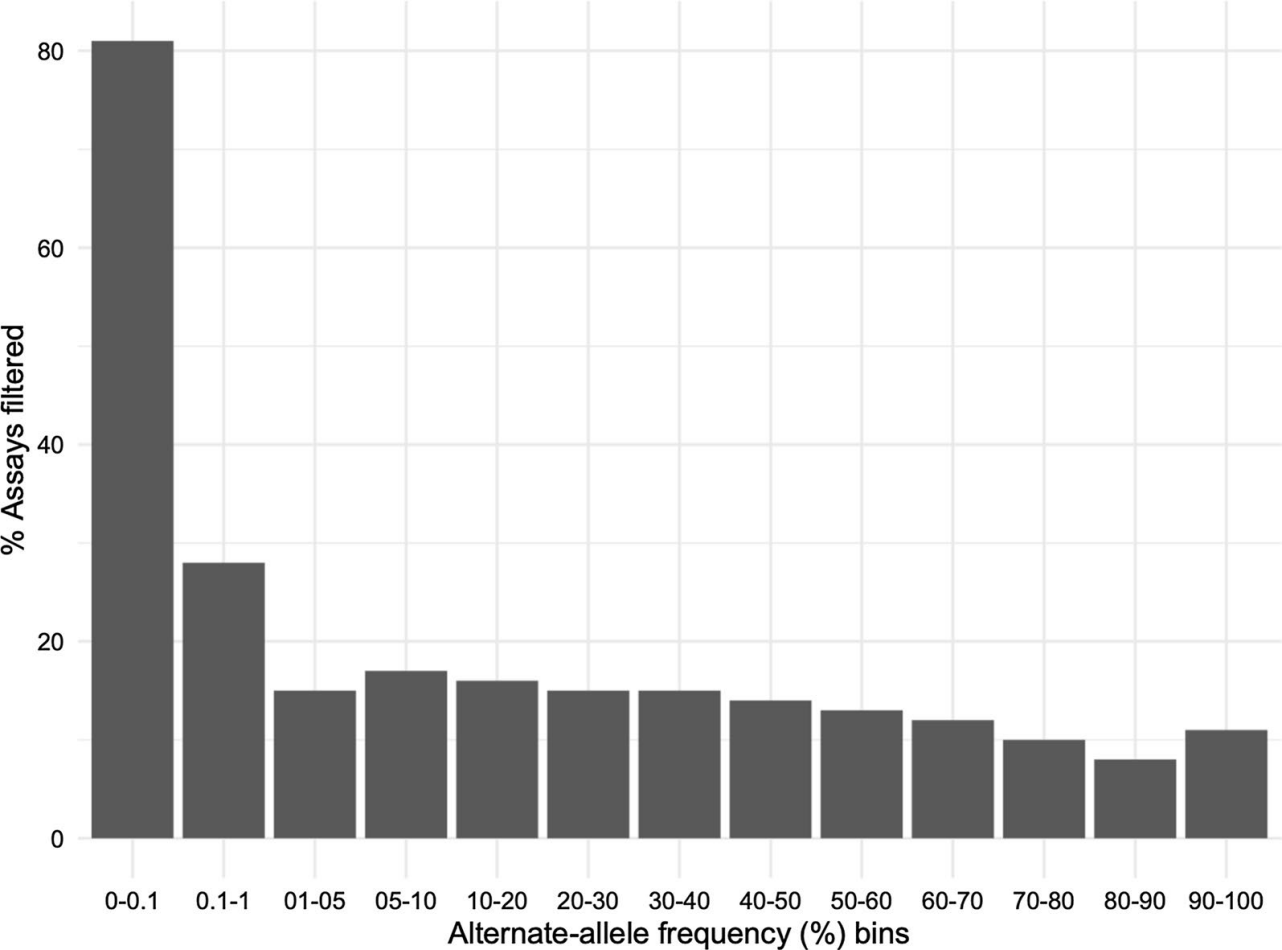
Bar plot of percentage of GSA assays with a positive predictive value (PPV) < 1 as a function of alternate allele frequency bins (allele frequency bins as percentage). The alternate allele frequency bins were defined based on the frequency information in 1000 Genomes (1KG) data

Among the 594,346 GSA SNV assays with IKG genotypes, 476,707 had a PPV equal to 1 (zero false positives) based on concordance with the 1KG and rWGS genotypes (Table 8). We observed that 81% of GSA assays in the [0–0.1%] and 28% of GSA assays in the (0.1–1%] bins had PPV < 1 (Fig. 5; Table 9), whereas other allele frequency bins had an average of 13% (8–17%) with a PPV < 1 (Table 8). These results are consistent with prior observations showing that accurate calling of rare alleles (MAF < 0.01) by genotyping arrays is compromised by low genotype frequencies and an absence of the homozygous alternate alleles needed for construction of cluster files [22, 37].

**Table 8:** GSA assays with a PPV = 1 based on concordance with the1KG Phase 3 data and the rWGS data. Data is binned by alternate allele frequency

Allele frequency bins	Total QC pass assays	Assays with PPV = 1; GSA versus 1KG and GSA versus rWGS	% left	% filtered
0–0.1	17,454	3283	19	81
0.1–1	138,342	99,508	72	28
1–5	104,272	88,235	85	15
5–10	58,421	48,532	83	17
10–20	78,631	66,027	84	16
20–30	52,398	44,511	85	15
30–40	36,620	31,223	85	15
40–50	27,476	23,563	86	14
50–60	23,053	20,041	87	13
60–70	20,134	17,799	88	12
70–80	19,210	17,318	90	10
80–90	14,312	13,103	92	8
90–100	4023	3564	89	11
Total	594,346	476,707

**Table 9:** Summary of performance metrics for GSA and rWGS relative to 1KG Phase 3 data

Performance metrics	Global screening array (GSA) versus. 1KG mean (± std.dev)	Whole genome sequencing (WGS) versus 1KG mean (± std.dev)
Concordance	0.9932 (± 0.0005)	0.9981 (± 0.0005)
Sensitivity	0.9927 (± 0.0007)	0.9981 (± 0.0005)
Specificity	0.9957 (± 0.0003)	0.9991 (± 0.0003)
Positive predictive value (PPV)	0.9892 (± 0.0008)	0.9977 (± 0.0007)

### GSA assays interrogating low-complexity genomic regions perform poorer than other assays

To determine assay performance characteristics within repetitive regions of the genome, we intersected GSA assays with annotated low complexity regions (LCRs) including simple repeats, microsatellites, and repeat masked (RepeatMasker-defined) regions in the human genome. Of a total of 594,346 assays passing QC and present in the 1KG, 203,901 (~ 34%) assessed a variant within one of the three annotation classes. 201,579 GSA assays mapped within the RepeatMasker class. Overlapping partially with the other two classes, 431 GSA assays mapped within the simple repeat class. GSA assays targeting genotypes within each LCR class had poorer performance metrics than did assays interrogating genotypes outside of these regions (Fig. 4D).

### rWGS performed better than GSA relative to the benchmark dataset 1KG

rWGS data corresponding to GSA assays passing QC were extracted from the rWGS gVCF files and compared to the 1KG. Restricting the analyses to GSA assays for which > 90% of rWGS samples had genotype data defined 602,582 assays and excluded 38,093 GSA assays. An additional 9642 assays on the chromosome X were excluded due to discrepancies in genotype representation in comparison datasets. For the remaining 592,940 autosomal assays, the rWGS genotypes with ≥ 20 × coverage and a Phred score ≥ 30 were used for calculation of performance metrics. These analyses, i.e., GSA vs. 1KG and rWGS vs. 1KG, showed consistent average metrics and small standard deviations among datasets (Table 9).

**Fig. 6:**
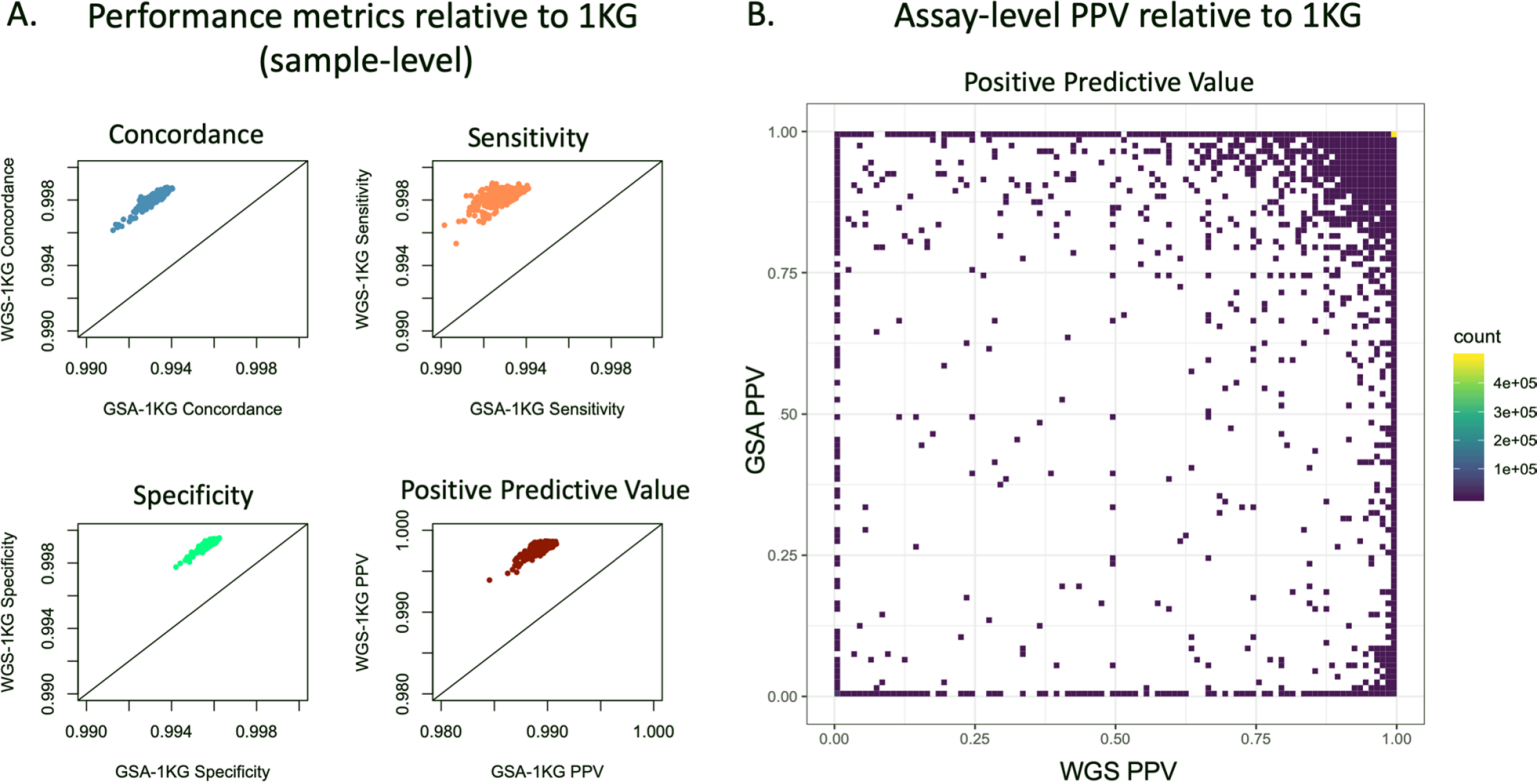
Scatter-plot comparison of performance metrics of whole genome sequencing (rWGS) and GSA using 1KG as the benchmark dataset. **A** Scatter plots show sample-level performance metrics of rWGS and GSA relative to 1KG reference data. Plots are concordance (top left; blue), sensitivity (top right; orange), specificity (bottom left; green) and positive predictive value (PPV) (bottom right; maroon) respectively. Each dot represents a single sample’s performance metric value. **B** Density scatterplot of each GSA assay’s positive predictive value computed for GSA (y-axis) vs. rWGS (x-axis) using 1KG as the benchmark dataset. Each square represents PPV measured for GSA and rWGS relative to 1KG benchmark dataset, and the color indicates the number of assays within each square. The color gradient of each square ranges from 1 assay (dark purple) to 476,828 assays (yellow); therefore, the color on the scatterplot indicates the density of data-points in 2 dimensions

For the 256 Coriell samples with 1KG data, we observed that rWGS performed better than GSA across all 4 performance metrics (Fig. 6A; Table 9). Overall average concordance, sensitivity, and specificity for rWGS vs. 1KG were 0.9981, 0.9981 and 0.9991, respectively, whereas for GSA vs. 1KG, they were 0.9932, 0.9927, and 0.9957, respectively. PPV was 0.9977 for rWGS vs. 1KG and was 0.9892 for GSA vs. 1KG (Table 9).

### Over 82% of all GSA assays have a PPV = 1

We compared the GSA and rWGS genotypes to the 1KG and computed the PPV. As shown in Fig. 6B, over 82% (476,828) of assays had a PPV of 1 for both the GSA and rWGS. Approximately 1.5% (8710) of rWGS assays had a PPV of 1 when GSA was 0, whereas only 0.12% (699) of GSA assays did when rWGS was 0.

### GSA MAP59 secondary findings validated using rWGS, pWGS, and 1KG

**Fig. 7:**
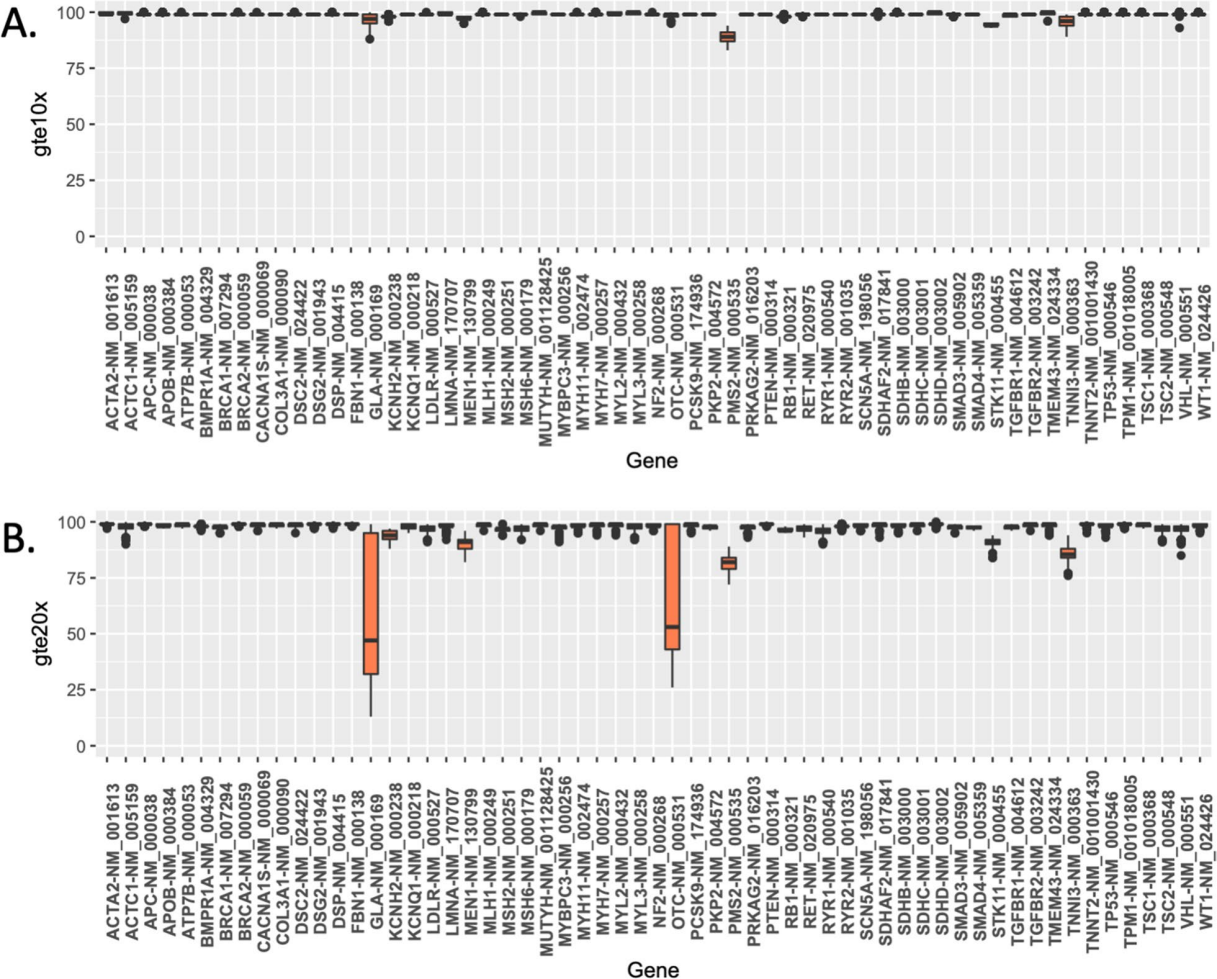
Plot of the average percentage of bases within each MAP59 gene covered by whole genome sequencing (rWGS) to a read depth of **A** ×10 or more (*gte10x*) **B** ×20 or more (*gte20x*) among the 263 samples. Each rWGS nucleotide was required to have a Phred-based quality score of greater than 30 to be considered for this analysis

Given that > 80% of GSA assays have a PPV = 1, we assessed rare variation detection within the 59 medically actionable predisposition genes (MAP59) defined by the American College of Medical Genetics (ACMG) [35]. Given the expected secondary finding rate of 1–2% [38–40] and the limited genomic space profiled by the GSA, we hypothesized 2–3 or fewer samples with GSA-detectable variants in the 261 cohort. Additionally, we hypothesized that comparison of these data to the 1KG and the rWGS data identifies false negative and false positive variants as well as pathogenic variation undetected by the GSA. Focusing on nucleotides with ≥ 20 × rWGS coverage (Fig. 7), we found that an average of 6347 (± 88) sites were genotyped by both rWGS and GSA in any given DNA sample. The GSA vs. rWGS average concordance, sensitivity, specificity, and PPV were 0.99897, 0.99367, 0.99962, and 0.9946, respectively.

For clinically reportable rare variants curated into the managed variant list (MVL), the GSA and rWGS were concordant for a heterozygous variant (*MUTYH* p.(Gly368Asp); rs36053993) in three samples and across GSA replicates. Two of the 3 samples had 1KG data and were concordant; one of these two had pWGS data that was also concordant. Highlighting the potential for false positives, rWGS and 1KG data refuted a GSA call of *PKP2* p.(Arg355Ter) (rs754912778) in one sample. Conversely, highlighting the potential for false negatives, rWGS and 1KG detected two variants that were not detected by GSA: *RB1* p.(Arg661Trp) (rs137853294), which the GSA called homozygous reference in triplicate, and *MUTYH* p.(Pro391Leu) (rs529008617), which the GSA called “no-call” in triplicate. In summary, the GSA identified 1 pathogenic variant (true positive), 1 false positive, and 2 false negatives (2 assayed and missed) among the MAP59.

To identify rare pathogenic variation discovered by rWGS and not assayed by the GSA (lack of probe coverage), we intersected rWGS data with ClinVar pathogenic variation and found 4 heterozygote variants not assayed by the GSA. These were *APOB* p.(Arg3527Trp) (rs144467873), *SDHAF2* p.(Asn103GlufsTer4) (frameshift insertion; rs753554501), *BRCA2* p.(Ser1748Ter) (insertion (NM_000059.3:c.5241_5242insTA); rs749980674) and *ATP7B* p.(Thr991Met) (rs41292782). One of these 4 (*APOB* p.(Arg3527Trp); rs144467873) was present in 1KG. The *ATP7B* p.(Thr991Met) (rs41292782) variant was likely absent from the 1KG due to poor coverage. In summary, rWGS identified 7 rare pathogenic variants in MAP59 genes in 9 samples; the GSA lacked assays for 4 rare pathogenic variants detected by WGS.

The rWGS rate of detection of rare pathogenic variants in the MAP59 genes was 0.034 (3.4%); 7 variants in 9 samples from a population of 261. Removing the 3 variants that were not independently confirmed by the 1KG due to lack of 1KG data gives 4 pathogenic variants in 5 individuals from a population of 261 or a rate of 0.019 (1.9%). This range (0.019–0.034) of pathogenic variants in the MAP59 genes is consistent with the published discovery rate [38, 39, 41, 42].

## Discussion

We report an approach to analytical validation of the GSA through quality analyses and through assessment of performance by comparison to benchmark datasets and independent whole-genome sequencing data. To the best of our knowledge, this is the first comprehensive analytical validation of the GSA for clinical genotyping. Our findings support and extend recently reported research studies assessing the utility of the GSA for genetic screening in primary immunodeficiency [43], for population-based genomic screening for rare and medically relevant variation [44], and for detecting rare and clinically relevant markers in multiethnic Indian populations [45].

In our study we used call rate and sample contamination as preliminary parameters of quality control for genotype analysis. Call rate is a primary quality control parameter in all genotyping studies [12, 13]. A high threshold for call rate not only ensures inclusion of samples with high quality genotype data but also allows, independent of sample DNA quality, for detection of assays that perform poorly. Additionally, sample contamination detection [14] is key in preventing return of false positive genotypes and is demonstrated by our results. While more advanced quality control methods such as Hardy–Weinberg Equilibrium (HWE) test [15], likelihood of error [19], departure from Mendelian inheritance, and pedigree information are used in various research studies [4, 20], they are implemented in analyses that follow genotype generation and are dependent on what analyses are subsequently performed using the genotype data. HWE is used to detect genotypes that deviate from the expectation of HWE, and it is typically applied to variants with a MAF of greater than 0.05 [12]. Consequently, because of our interest in variants of lower MAF, we did not implement this QC metric; however, HWE might be useful within certain cut-offs for MAF as implemented by Suratannon et al. [43] and Narang et al. [45]. Similarly, Mendelian inheritance and pedigree information quality control are critical for linkage and segregation analyses and did not apply to our individual-focused assay.

This evaluation of GSA data is consistent with previous studies that demonstrated the utility of sample data quality metrics like genotype call-rate, p10GC, and DNA contamination detection [11, 22]. By analysis of replicates, we show that the majority of the GSA data are highly reproducible. Outliers arose either from positioning along the edges of the Illumina BeadChip or from contamination. Characterization of each GSA assay by variation class, type, genomic DNA complexity, and alternate allele frequency showed that the GSA has the highest performance for SNVs and transition nucleotide changes in genomic regions of high complexity. In contrast, assays interrogating low-complexity regions, rare alleles, or transversions performed poorly. Transversions between complementary nucleotides likely performed poorly because of the characteristics of the assays for these particular transversions (Additional file 1: Sect. S7). Also, consistent with previous reports [46–48], assays for rare alleles (< 0.001) had lower performance and might be improved by using algorithms for rare variant detection [10, 31, 32] or joint-calling [22] rather than the default genotype caller (GenCall). These should be considered in the future to improve detection of rare variants by genotyping chips.

The analytical framework implemented in this study followed a three-way analysis (GSA-rWGS-1KG) to assess the strengths and limitations of individual GSA assays. Unlike many published analyses in which WGS is the test dataset and the BeadArray genotypes are the truth [25–27, 30], our study had the BeadArray as the test dataset and WGS as the truth. The reversal of test and truth datasets is a major challenge for comparing our results to the published literature. To overcome this challenge, we ensured that the rWGS data had performance metrics (concordance = 0.9981) comparable to that previously published (concordance = 0.9984 [25]). The three-way analysis framework also allowed detection of false positive and false negative genotypes on the GSA platform. Though not evaluated in the current study, the three-way comparison framework in our analysis allows for modeling of genotyping-error specific to variation classes and categories triaged during characterization of the GSA.

Over 82% of assays on the GSA returned genotypes with a high positive predictive value (PPV). The GSA detected some pathogenic variation (MAP59) in the test dataset of 261 Coriell samples, and these variants were independently validated by either the 1KG data or the rWGS/pWGS data or both. Although we attempted to compare GSA results to other chip results, the comparison to previous work was impeded by differences in probe content and density as well as chip design (e.g., 610 k assays on GSA, vs. 247 k assays on HumanExome chip). Some of the pros and cons of using the GSA are summarized in Table 10 below.

**Table 10:** Pros and cons of arrays versus whole genome sequencing [49]

Feature	SNP arrays (GSA)	WGS
Cost	Lower cost	Higher cost
Genomic coverage	Best for variants for which DNAs of all genotype combinations are available, i.e., not robust for rare variants	Appropriate for detection of nearly all genetic variation in the genome depending on the depth of sequencing, i.e., not robust for difficult to sequence regions
	Requires prior knowledge of the variant, i.e., unable to detect private variants not previously reported	Reduced accuracy in genomic regions of low complexity
	Reduced accuracy in genomic regions of low complexity	
Analyses	Well established analytical protocols and tools for data analyses	High computational costs and greater analytical complexity
		Larger multiple testing burden when conducting single-variant tests
		Greater costs to store, process, analyze and interpret the resulting data
Suitability	Screening	Diagnostic testing
	Analyzing known or candidate associations in large cohorts	Detecting and fine-mapping rare variants
	Detecting low-frequency, common variant associations in large sample sizes	Detecting ultra-rare risk variants when it becomes economically viable to perform WGS at a very large scale

The test characteristics of the GSA compared to WGS clearly show that the GSA is not a diagnostic genomic test for individuals with rare disorders. As shown by our MAP59 results and recent research studies [43, 44], the GSA lacks robustness for genotyping rare variants as well as probes for detection of private familial disease variants. On the other hand, we show that the GSA has the analytical robustness to serve as a clinical screen for genotypes for which one can establish robust cluster files for the AA, AB, and BB genotypes. This is most easily accomplished for more common genotypes that contribute to polygenic predispositions to disease, particularly common diseases. Screening of an asymptomatic population to assess the likelihood of predisposition to a disease is well established within medicine, and examples include newborn screening for inborn errors of metabolism, mammography for breast cancer, and cholesterol levels for coronary artery disease [50, 51]. A major objective of screening tests is to reduce morbidity and mortality in the subject population through risk stratification to target surveillance, early detection, and treatment. With the characterization of genomic risk for drug responsiveness and predisposition to various cancers and cardiovascular disease [52–54], we propose that the GSA offers a potential clinical tool for genomic screening.

### Limitations of our study

Our comparison of BeadChip arrays to NGS and benchmark datasets has some limitations. Firstly, we evaluated our dataset using accepted algorithms. This did not take into account the benefits of consensus genotyping by multiple algorithms for GSA or NGS data; Hwang et al. found that consensus genotyping minimized false findings [47]. Secondly, cell-line derived variation or low-level somatic variation might also have contributed to differences between datasets [25]. Thirdly, we did not analyze variants close to or overlapping other variation in the same location, e.g., insertions/deletions and copy number variation, because these loci are eukaryotic mutation hotspots [55]. Fourthly, our analysis would benefit from comparison to variant benchmark datasets defined in more recent publications [47] and to NIST/GiAB datasets.

## Conclusions

We established the analytical validity of the GSA via a systematic approach utilizing benchmark and rWGS data to evaluate the performance of each assay. We highlight that the GSA assays interrogating rare variants, transversions, and variants within low-complexity regions need careful evaluation. GSA assays can be analytically validated to clinically screen for common genotypes predisposing to disease.

## Supplementary Information

**Additional file 1.** Supplementary figures and tables.

## Data Availability

The data were deposited to NCBI SRA and dbSNP databases (BioProject: PRJNA792997; Additional file 1: Sect. S8). BioSample metadata are available in the NCBI BioSample database (https://www.ncbi.nlm.nih.gov/biosample; accession numbers SAMN24495081—SAMN24495343) (Additional file 1: Table S8). The BAM files with accession numbers for WGS data (262 samples) are available in the Sequence Read Archive (SRA) database (https://www.ncbi.nlm.nih.gov/sra) (Additional file 1: Table S9). The GSA (replicated in triplicate; samples = 262) and WGS (QC pass and concordant with GSA samples = 260) variation data were submitted to dbSNP database (https://www.ncbi.nlm.nih.gov/snp) are available at https://ftp.ncbi.nlm.nih.gov/snp/submission/SANFORD_IMAGENETICS/.

## Abbreviations

SNV: Single nucleotide variation
MAV: Multi-allelic variant
DEL: Deletion
INS: Insertion
PPV: Positive predictive value
QC: Quality control
NGS: Next-generation sequencing
GSA: Global screening array
rWGS: Whole-genome sequencing
IKG: 1000 Genomes
HTS: High-throughput sequencing
VCF: Variant call file
AWS: Amazon web services
PCA: Principal component analysis
TP: True positive
TN: True negative
FP: False positive
FN: False negative
MAP: Medically actionable predisposition
LCR: Low-complexity regions
GiAB: Genome in a bottle
