# Supplementary material for: Establishing analytical validity of BeadChip array genotype data by comparison to whole-genome sequence and standard benchmark datasets
Source: BMC Med Genomics. 2022 Mar 14;15:56. doi: 10.1186/s12920-022-01199-8 (PMC8919546; doi:10.1186/s12920-022-01199-8)
Supplement: Supplementary file 1 — Additional file 1. Supplementary figures and tables. [file 12920_2022_1199_MOESM1_ESM.docx]

Supplementary Information

Additional file 1

Table of Contents

1 Samples and Infinium Global Screening Array (GSA) cluster file generation 2

2 Study sample selection rationale 8

3 Data, Datasets, and Data organization 8

3.1 WGS data 10

4 Principal Component Analysis (PCA) 12

4.1 Methods 12

4.1.1 Datasets 12

4.1.2 Principal Component Analysis 12

4.1.3 PCA analysis testing – randomization experiment 13

4.2 Results 13

4.3 R code 16

5 Sample DNA contamination estimation and detection 17

5.1 Regression-based method to detect sample contamination 17

6 GSA quality control analyses 20

6.1 Quality control for BeadChip Array 20

6.2 Quality control for GSA triplicate samples 21

6.3 Quality control of GSA triplicate VCF files 22

7 Transitions and transversions 23

7.1 Transitions and transversions with special focus on complementary transversions 24

8 Data submitted to NCBI 25

References 40

Samples and Infinium Global Screening Array (GSA) cluster file generation

List of 263 samples purchased from the Coriell Institute for GSA analysis are listed in Table S1 (details of sample selection in next section). A total of 7 plates containing Coriell samples across 14 diversity panels and 5 plates of Sanford Biobank samples were run in order to create a cluster file for the GSA with custom content (Sanford chip). Each plate was run 3 times (by 3 different technicians, on two different robots, using 2 lots of the Infinium HTS kit). Genotyping data was generated for 1,104 individuals in triplicate. The proportion of females to males was roughly 1:1 and the ethnic composition was as follows:

Of a total of 1,415 Coriell samples that were purchased from the Coriell Institute (Table S2), 644 Coriell samples covering major ethnic groups (46 individuals per each group) were used:

- Finns in Finland
- Han Chinese
- British in the United Kingdom
- Puerto Ricans in Puerto Rico
- Mexicans in Los Angeles, USA
- Japanese in Tokyo
- Iberians in Spain
- Peruvians in Lima
- Yoruba in Ibadan
- Kinh in Ho Chi Minh City
- African ancestry in Southwest US
- African Caribbean in Barbados
- Mende in Sierra Leone
- Indian Telugu in the UK

460 individuals semi-randomly selected from the local population (from the Sanford Biobank) including Native Americans (50) and trios (10) from internal research.

With the exception of the Native Americans and research trios, samples from the biobank were selected to assess two different methods of DNA extraction in the laboratory. To evaluate biases in cluster file generation at two physically different locations and from different input DNA samples, we compared a cluster file generated by Illumina to the one generated in-house. We found high correlation of p10GC values when the cluster files were compared indicating no major physical location specific or sample selection biases. Additionally, the utility of the GSA for assaying genotypes within the MAP59 genes was checked by overlaying MAP59 assays on all assays in the plot; this found no major differences between cluster files; minor off-diagonal differences were assays with different p10GC values relative to each other. The majority of the MAP59 assays had lower p10GC values in both the Illumina cluster file and the Imagenetics cluster file (Figure S1). Assessment of the reproducibility of cluster file curation between institutes was performed in collaboration with the bioinformatics staff at Illumina, Inc. (https://www.illumina.com, Ryan Kelly and Jeremy Pierce, unpublished data).

**Table S1.** List of 263 Coriell DNA samples purchased from the Coriell Institute and used for GSA analytical validity analyses.

| HG00096 | HG00345 | HG02052 | HG03705 | NA18543 | NA19023 | NA19314 | NA19457 | NA20276 | NA20519 |
| --- | --- | --- | --- | --- | --- | --- | --- | --- | --- |
| HG00099 | HG00366 | HG02271 | HG03713 | NA18544 | NA19024 | NA19319 | NA19466 | NA20278 | NA20520 |
| HG00101 | HG00403 | HG02304 | HG03742 | NA18552 | NA19026 | NA19320 | NA19468 | NA20287 | NA20524 |
| HG00102 | HG00419 | HG02360 | HG03788 | NA18559 | NA19031 | NA19321 | NA19471 | NA20289 | NA20525 |
| HG00103 | HG00452 | HG02568 | HG03790 | NA18572 | NA19036 | NA19323 | NA19472 | NA20291 | NA20532 |
| HG00106 | HG00759 | HG02628 | HG03848 | NA18606 | NA19037 | NA19324 | NA19473 | NA20294 | NA20763 |
| HG00108 | HG01051 | HG02651 | HG03854 | NA18856 | NA19041 | NA19327 | NA19475 | NA20296 | NA20767 |
| HG00110 | HG01083 | HG02684 | HG03871 | NA18861 | NA19042 | NA19347 | NA19625 | NA20299 | NA20812 |
| HG00111 | HG01089 | HG02922 | HG03885 | NA18915 | NA19043 | NA19350 | NA19648 | NA20320 | NA20814 |
| HG00116 | HG01112 | HG03006 | HG03896 | NA18916 | NA19056 | NA19355 | NA19658 | NA20321 | NA20822 |
| HG00118 | HG01170 | HG03052 | HG03967 | NA18933 | NA19070 | NA19374 | NA19682 | NA20332 | NA20845 |
| HG00120 | HG01398 | HG03267 | HG03974 | NA18939 | NA19072 | NA19376 | NA19700 | NA20339 | NA20846 |
| HG00122 | HG01441 | HG03279 | HG04060 | NA18944 | NA19076 | NA19380 | NA19701 | NA20342 | NA20863 |
| HG00126 | HG01455 | HG03366 | HG04094 | NA18948 | NA19102 | NA19390 | NA19704 | NA20346 | NA20864 |
| HG00133 | HG01500 | HG03369 | HG04131 | NA18953 | NA19119 | NA19395 | NA19713 | NA20348 | NA20867 |
| HG00174 | HG01530 | HG03401 | HG04219 | NA18959 | NA19130 | NA19399 | NA19764 | NA20351 | NA20887 |
| HG00185 | HG01565 | HG03464 | NA06989 | NA18965 | NA19131 | NA19403 | NA19780 | NA20355 | NA20889 |
| HG00188 | HG01583 | HG03472 | NA07045 | NA18966 | NA19147 | NA19429 | NA19789 | NA20356 | NA20890 |
| HG00253 | HG01595 | HG03514 | NA12156 | NA18970 | NA19159 | NA19434 | NA19795 | NA20412 | NA20901 |
| HG00257 | HG01605 | HG03575 | NA12878 | NA18971 | NA19160 | NA19435 | NA19834 | NA20502 | NA20904 |
| HG00263 | HG01620 | HG03642 | NA17281 | NA18974 | NA19189 | NA19436 | NA19835 | NA20509 | NA21090 |
| HG00265 | HG01879 | HG03681 | NA18486 | NA18983 | NA19209 | NA19437 | NA19909 | NA20510 | NA21093 |
| HG00266 | HG01941 | HG03685 | NA18488 | NA18990 | NA19238 | NA19440 | NA19921 | NA20511 | NA21105 |
| HG00268 | HG01944 | HG03686 | NA18508 | NA18994 | NA19239 | NA19443 | NA19984 | NA20513 | NA21118 |
| HG00276 | HG02012 | HG03687 | NA18511 | NA19017 | NA19248 | NA19445 | NA20126 | NA20515 | NA21128 |
| HG00332 | HG02032 | HG03694 | NA18525 | NA19019 | NA19308 | NA19452 | NA20274 | NA20516 | NA21135 |
| NA24143 | NA24149 | NA24385 |  |  |  |  |  |  |  |

**Table S2.** List of 1,415 Coriell DNA samples purchased from the Coriell Institute for GSA for the purpose of cluster file generation.

| HG00096 | HG02397 | HG03905 | NA18644 | NA19473 | HG01619 | HG03556 | NA11995 | NA19087 | NA20543 |
| --- | --- | --- | --- | --- | --- | --- | --- | --- | --- |
| HG00099 | HG02398 | HG03907 | NA18645 | NA19474 | HG01620 | HG03558 | NA12003 | NA19088 | NA20544 |
| HG00101 | HG02399 | HG03908 | NA18646 | NA19475 | HG01625 | HG03559 | NA12004 | NA19089 | NA20581 |
| HG00102 | HG02401 | HG03910 | NA18647 | NA19625 | HG01630 | HG03563 | NA12006 | NA19090 | NA20586 |
| HG00103 | HG02402 | HG03911 | NA18648 | NA19648 | HG01631 | HG03565 | NA12043 | NA19091 | NA20587 |
| HG00106 | HG02406 | HG03913 | NA18740 | NA19649 | HG01669 | HG03567 | NA12044 | NA19092 | NA20588 |
| HG00108 | HG02407 | HG03914 | NA18749 | NA19651 | HG01675 | HG03571 | NA12144 | NA19093 | NA20589 |
| HG00110 | HG02408 | HG03916 | NA18853 | NA19652 | HG01686 | HG03572 | NA12154 | NA19095 | NA20752 |
| HG00111 | HG02409 | HG03917 | NA18856 | NA19654 | HG01705 | HG03575 | NA12155 | NA19096 | NA20753 |
| HG00116 | HG02410 | HG03919 | NA18858 | NA19655 | HG01756 | HG03577 | NA12156 | NA19098 | NA20754 |
| HG00118 | HG02420 | HG03920 | NA18861 | NA19657 | HG01765 | HG03578 | NA12249 | NA19099 | NA20755 |
| HG00120 | HG02429 | HG03922 | NA18864 | NA19658 | HG01777 | HG03583 | NA12273 | NA19102 | NA20758 |
| HG00122 | HG02433 | HG03925 | NA18865 | NA19661 | HG01783 | HG03585 | NA12282 | NA19107 | NA20759 |
| HG00126 | HG02439 | HG03926 | NA18867 | NA19664 | HG01791 | HG03589 | NA12283 | NA19108 | NA20760 |
| HG00133 | HG02442 | HG03928 | NA18868 | NA19669 | HG01810 | HG03593 | NA12287 | NA19113 | NA20761 |
| HG00148 | HG02449 | HG03931 | NA18870 | NA19670 | HG01811 | HG03594 | NA12340 | NA19114 | NA20762 |
| HG00160 | HG02455 | HG03934 | NA18871 | NA19676 | HG01816 | HG03595 | NA12348 | NA19116 | NA20763 |
| HG00174 | HG02461 | HG03937 | NA18873 | NA19678 | HG01840 | HG03598 | NA12383 | NA19117 | NA20765 |
| HG00183 | HG02470 | HG03940 | NA18874 | NA19679 | HG01842 | HG03603 | NA12414 | NA19118 | NA20767 |
| HG00185 | HG02484 | HG03943 | NA18876 | NA19681 | HG01844 | HG03604 | NA12489 | NA19119 | NA20770 |
| HG00186 | HG02489 | HG03947 | NA18877 | NA19682 | HG01846 | HG03607 | NA12716 | NA19129 | NA20771 |
| HG00187 | HG02490 | HG03949 | NA18878 | NA19684 | HG01849 | HG03611 | NA12717 | NA19130 | NA20775 |
| HG00188 | HG02496 | HG03950 | NA18881 | NA19700 | HG01852 | HG03615 | NA12748 | NA19131 | NA20778 |
| HG00244 | HG02512 | HG03951 | NA18907 | NA19701 | HG01860 | HG03616 | NA12749 | NA19137 | NA20783 |
| HG00253 | HG02521 | HG03953 | NA18908 | NA19703 | HG01861 | HG03624 | NA12750 | NA19138 | NA20785 |
| HG00257 | HG02536 | HG03963 | NA18909 | NA19704 | HG01864 | HG03629 | NA12751 | NA19141 | NA20786 |
| HG00263 | HG02541 | HG03965 | NA18910 | NA19707 | HG01865 | HG03631 | NA12761 | NA19143 | NA20787 |
| HG00264 | HG02545 | HG03967 | NA18912 | NA19711 | HG01866 | HG03634 | NA12763 | NA19144 | NA20792 |
| HG00265 | HG02554 | HG03968 | NA18915 | NA19712 | HG01867 | HG03636 | NA12776 | NA19146 | NA20795 |
| HG00266 | HG02557 | HG03969 | NA18916 | NA19713 | HG01872 | HG03640 | NA12812 | NA19147 | NA20796 |
| HG00267 | HG02568 | HG03971 | NA18917 | NA19716 | HG01873 | HG03642 | NA12813 | NA19149 | NA20798 |
| HG00268 | HG02585 | HG03973 | NA18923 | NA19717 | HG01879 | HG03643 | NA12815 | NA19152 | NA20801 |
| HG00271 | HG02597 | HG03974 | NA18924 | NA19719 | HG01885 | HG03644 | NA12828 | NA19153 | NA20802 |
| HG00276 | HG02600 | HG03976 | NA18933 | NA19720 | HG01914 | HG03645 | NA12829 | NA19159 | NA20803 |
| HG00277 | HG02603 | HG03977 | NA18934 | NA19722 | HG01917 | HG03646 | NA12830 | NA19160 | NA20805 |
| HG00278 | HG02610 | HG03978 | NA18939 | NA19723 | HG01941 | HG03649 | NA12872 | NA19171 | NA20806 |
| HG00280 | HG02613 | HG03986 | NA18940 | NA19725 | HG01944 | HG03652 | NA12873 | NA19172 | NA20809 |
| HG00284 | HG02620 | HG03989 | NA18942 | NA19729 | HG01947 | HG03653 | NA12878 | NA19175 | NA20810 |
| HG00310 | HG02623 | HG03990 | NA18943 | NA19732 | HG01950 | HG03660 | NA12889 | NA19184 | NA20811 |
| HG00311 | HG02628 | HG03995 | NA18944 | NA19735 | HG01953 | HG03668 | NA17281 | NA19185 | NA20812 |
| HG00321 | HG02634 | HG03998 | NA18945 | NA19741 | HG01961 | HG03673 | NA18486 | NA19189 | NA20814 |
| HG00329 | HG02651 | HG03999 | NA18946 | NA19747 | HG01970 | HG03680 | NA18488 | NA19190 | NA20815 |
| HG00332 | HG02654 | HG04001 | NA18948 | NA19749 | HG01974 | HG03681 | NA18489 | NA19197 | NA20822 |
| HG00335 | HG02657 | HG04002 | NA18949 | NA19750 | HG01977 | HG03684 | NA18498 | NA19198 | NA20827 |
| HG00345 | HG02660 | HG04003 | NA18950 | NA19752 | HG01979 | HG03685 | NA18499 | NA19200 | NA20845 |
| HG00351 | HG02681 | HG04006 | NA18951 | NA19756 | HG01982 | HG03686 | NA18501 | NA19201 | NA20846 |
| HG00360 | HG02684 | HG04014 | NA18952 | NA19759 | HG01986 | HG03687 | NA18502 | NA19204 | NA20847 |
| HG00366 | HG02727 | HG04015 | NA18953 | NA19761 | HG01988 | HG03691 | NA18504 | NA19206 | NA20849 |
| HG00369 | HG02789 | HG04017 | NA18954 | NA19762 | HG01990 | HG03693 | NA18505 | NA19207 | NA20850 |
| HG00372 | HG02813 | HG04018 | NA18956 | NA19764 | HG02002 | HG03694 | NA18507 | NA19209 | NA20851 |
| HG00375 | HG02816 | HG04020 | NA18957 | NA19770 | HG02008 | HG03695 | NA18508 | NA19210 | NA20852 |
| HG00382 | HG02819 | HG04022 | NA18959 | NA19771 | HG02009 | HG03697 | NA18510 | NA19213 | NA20853 |
| HG00403 | HG02860 | HG04033 | NA18960 | NA19773 | HG02012 | HG03698 | NA18511 | NA19214 | NA20854 |
| HG00419 | HG02881 | HG04035 | NA18961 | NA19774 | HG02013 | HG03702 | NA18516 | NA19222 | NA20856 |
| HG00421 | HG02895 | HG04038 | NA18962 | NA19776 | HG02014 | HG03703 | NA18517 | NA19223 | NA20858 |
| HG00442 | HG02922 | HG04039 | NA18963 | NA19777 | HG02017 | HG03705 | NA18519 | NA19225 | NA20859 |
| HG00445 | HG02947 | HG04042 | NA18964 | NA19779 | HG02020 | HG03706 | NA18520 | NA19235 | NA20861 |
| HG00448 | HG03006 | HG04047 | NA18965 | NA19780 | HG02023 | HG03708 | NA18522 | NA19236 | NA20862 |
| HG00452 | HG03052 | HG04059 | NA18966 | NA19782 | HG02026 | HG03709 | NA18523 | NA19238 | NA20863 |
| HG00457 | HG03120 | HG04060 | NA18967 | NA19783 | HG02029 | HG03711 | NA18525 | NA19239 | NA20864 |
| HG00472 | HG03166 | HG04061 | NA18968 | NA19786 | HG02032 | HG03713 | NA18526 | NA19247 | NA20866 |
| HG00475 | HG03202 | HG04062 | NA18970 | NA19788 | HG02035 | HG03714 | NA18528 | NA19248 | NA20867 |
| HG00478 | HG03212 | HG04076 | NA18971 | NA19789 | HG02040 | HG03716 | NA18530 | NA19256 | NA20868 |
| HG00500 | HG03224 | HG04080 | NA18972 | NA19792 | HG02047 | HG03720 | NA18531 | NA19257 | NA20869 |
| HG00524 | HG03225 | HG04090 | NA18973 | NA19794 | HG02050 | HG03727 | NA18532 | NA19307 | NA20870 |
| HG00530 | HG03234 | HG04093 | NA18974 | NA19795 | HG02051 | HG03729 | NA18533 | NA19308 | NA20872 |
| HG00533 | HG03235 | HG04094 | NA18975 | NA19818 | HG02052 | HG03730 | NA18534 | NA19309 | NA20874 |
| HG00556 | HG03237 | HG04098 | NA18976 | NA19819 | HG02053 | HG03731 | NA18535 | NA19310 | NA20875 |
| HG00580 | HG03238 | HG04100 | NA18977 | NA19834 | HG02058 | HG03736 | NA18536 | NA19312 | NA20877 |
| HG00595 | HG03240 | HG04107 | NA18978 | NA19835 | HG02061 | HG03742 | NA18537 | NA19314 | NA20881 |
| HG00598 | HG03241 | HG04118 | NA18979 | NA19900 | HG02064 | HG03744 | NA18538 | NA19315 | NA20882 |
| HG00607 | HG03246 | HG04131 | NA18980 | NA19901 | HG02067 | HG03746 | NA18539 | NA19316 | NA20884 |
| HG00619 | HG03247 | HG04134 | NA18981 | NA19904 | HG02070 | HG03753 | NA18541 | NA19317 | NA20885 |
| HG00625 | HG03258 | HG04140 | NA18982 | NA19908 | HG02073 | HG03754 | NA18542 | NA19318 | NA20887 |
| HG00650 | HG03265 | HG04141 | NA18983 | NA19909 | HG02076 | HG03755 | NA18543 | NA19319 | NA20888 |
| HG00653 | HG03267 | HG04144 | NA18984 | NA19913 | HG02079 | HG03757 | NA18544 | NA19320 | NA20889 |
| HG00662 | HG03268 | HG04146 | NA18985 | NA19914 | HG02082 | HG03760 | NA18546 | NA19321 | NA20890 |
| HG00674 | HG03270 | HG04152 | NA18986 | NA19916 | HG02085 | HG03762 | NA18547 | NA19323 | NA20891 |
| HG00683 | HG03271 | HG04153 | NA18988 | NA19917 | HG02088 | HG03765 | NA18548 | NA19324 | NA20894 |
| HG00689 | HG03279 | HG04155 | NA18989 | NA19920 | HG02090 | HG03770 | NA18549 | NA19327 | NA20895 |
| HG00692 | HG03291 | HG04156 | NA18990 | NA19921 | HG02104 | HG03771 | NA18550 | NA19328 | NA20896 |
| HG00701 | HG03294 | HG04158 | NA18991 | NA19922 | HG02107 | HG03774 | NA18552 | NA19331 | NA20897 |
| HG00707 | HG03298 | HG04159 | NA18993 | NA19923 | HG02116 | HG03777 | NA18553 | NA19332 | NA20901 |
| HG00759 | HG03300 | HG04161 | NA18994 | NA19982 | HG02122 | HG03778 | NA18555 | NA19334 | NA20902 |
| HG00844 | HG03303 | HG04162 | NA18995 | NA19984 | HG02128 | HG03779 | NA18557 | NA19338 | NA20903 |
| HG00881 | HG03311 | HG04164 | NA18997 | NA20126 | HG02131 | HG03780 | NA18558 | NA19346 | NA20904 |
| HG00982 | HG03342 | HG04171 | NA18998 | NA20127 | HG02134 | HG03781 | NA18559 | NA19347 | NA20905 |
| HG01028 | HG03343 | HG04173 | NA18999 | NA20274 | HG02137 | HG03782 | NA18560 | NA19350 | NA20906 |
| HG01031 | HG03351 | HG04176 | NA19000 | NA20276 | HG02138 | HG03787 | NA18561 | NA19351 | NA20908 |
| HG01051 | HG03366 | HG04177 | NA19001 | NA20278 | HG02141 | HG03788 | NA18562 | NA19355 | NA20910 |
| HG01069 | HG03367 | HG04180 | NA19003 | NA20281 | HG02146 | HG03789 | NA18563 | NA19360 | NA20911 |
| HG01082 | HG03369 | HG04182 | NA19004 | NA20282 | HG02150 | HG03790 | NA18564 | NA19372 | NA21086 |
| HG01083 | HG03372 | HG04183 | NA19005 | NA20287 | HG02219 | HG03793 | NA18565 | NA19374 | NA21087 |
| HG01089 | HG03376 | HG04185 | NA19006 | NA20289 | HG02221 | HG03796 | NA18566 | NA19375 | NA21089 |
| HG01101 | HG03378 | HG04186 | NA19009 | NA20291 | HG02224 | HG03800 | NA18567 | NA19376 | NA21090 |
| HG01112 | HG03380 | HG04188 | NA19010 | NA20294 | HG02233 | HG03802 | NA18570 | NA19377 | NA21091 |
| HG01130 | HG03382 | HG04189 | NA19011 | NA20296 | HG02236 | HG03803 | NA18571 | NA19378 | NA21092 |
| HG01170 | HG03388 | HG04194 | NA19012 | NA20298 | HG02250 | HG03805 | NA18572 | NA19379 | NA21093 |
| HG01197 | HG03391 | HG04195 | NA19017 | NA20299 | HG02253 | HG03808 | NA18573 | NA19380 | NA21094 |
| HG01250 | HG03394 | HG04198 | NA19019 | NA20317 | HG02255 | HG03809 | NA18574 | NA19383 | NA21095 |
| HG01256 | HG03397 | HG04202 | NA19020 | NA20318 | HG02259 | HG03812 | NA18577 | NA19384 | NA21098 |
| HG01259 | HG03401 | HG04206 | NA19023 | NA20320 | HG02262 | HG03814 | NA18579 | NA19385 | NA21099 |
| HG01302 | HG03410 | HG04210 | NA19024 | NA20321 | HG02265 | HG03815 | NA18582 | NA19390 | NA21100 |
| HG01308 | HG03419 | HG04211 | NA19025 | NA20332 | HG02271 | HG03817 | NA18591 | NA19391 | NA21101 |
| HG01325 | HG03428 | HG04214 | NA19026 | NA20334 | HG02274 | HG03821 | NA18592 | NA19393 | NA21102 |
| HG01350 | HG03432 | HG04216 | NA19027 | NA20339 | HG02277 | HG03823 | NA18593 | NA19394 | NA21103 |
| HG01356 | HG03433 | HG04219 | NA19028 | NA20340 | HG02283 | HG03824 | NA18595 | NA19395 | NA21104 |
| HG01362 | HG03436 | HG04222 | NA19030 | NA20342 | HG02285 | HG03826 | NA18596 | NA19397 | NA21105 |
| HG01365 | HG03437 | HG04227 | NA19031 | NA20346 | HG02291 | HG03829 | NA18597 | NA19399 | NA21106 |
| HG01374 | HG03439 | HG04229 | NA19035 | NA20348 | HG02299 | HG03830 | NA18599 | NA19401 | NA21107 |
| HG01392 | HG03442 | HG04235 | NA19036 | NA20351 | HG02304 | HG03832 | NA18602 | NA19403 | NA21108 |
| HG01395 | HG03445 | HG04238 | NA19037 | NA20355 | HG02307 | HG03833 | NA18603 | NA19404 | NA21109 |
| HG01398 | HG03446 | HG04239 | NA19038 | NA20356 | HG02314 | HG03838 | NA18605 | NA19428 | NA21110 |
| HG01412 | HG03449 | NA06984 | NA19041 | NA20357 | HG02317 | HG03844 | NA18606 | NA19429 | NA21111 |
| HG01437 | HG03451 | NA06986 | NA19042 | NA20359 | HG02323 | HG03846 | NA18608 | NA19430 | NA21112 |
| HG01440 | HG03452 | NA06989 | NA19043 | NA20362 | HG02330 | HG03848 | NA18609 | NA19431 | NA21113 |
| HG01441 | HG03455 | NA06994 | NA19055 | NA20412 | HG02332 | HG03849 | NA18610 | NA19434 | NA21114 |
| HG01443 | HG03457 | NA07000 | NA19056 | NA20502 | HG02334 | HG03850 | NA18612 | NA19435 | NA21115 |
| HG01455 | HG03458 | NA07037 | NA19057 | NA20504 | HG02343 | HG03851 | NA18613 | NA19436 | NA21116 |
| HG01461 | HG03460 | NA07045 | NA19058 | NA20509 | HG02353 | HG03854 | NA18614 | NA19437 | NA21117 |
| HG01464 | HG03461 | NA07051 | NA19059 | NA20510 | HG02355 | HG03856 | NA18615 | NA19438 | NA21118 |
| HG01479 | HG03464 | NA07347 | NA19060 | NA20511 | HG02356 | HG03857 | NA18616 | NA19439 | NA21119 |
| HG01485 | HG03469 | NA07357 | NA19062 | NA20512 | HG02360 | HG03863 | NA18617 | NA19440 | NA21120 |
| HG01491 | HG03470 | NA10847 | NA19063 | NA20513 | HG02367 | HG03868 | NA18618 | NA19443 | NA21122 |
| HG01497 | HG03472 | NA10851 | NA19064 | NA20515 | HG02371 | HG03870 | NA18619 | NA19445 | NA21123 |
| HG01500 | HG03473 | NA11829 | NA19065 | NA20516 | HG02373 | HG03871 | NA18621 | NA19446 | NA21124 |
| HG01509 | HG03476 | NA11830 | NA19066 | NA20518 | HG02374 | HG03872 | NA18623 | NA19448 | NA21125 |
| HG01515 | HG03479 | NA11831 | NA19067 | NA20519 | HG02375 | HG03873 | NA18624 | NA19449 | NA21126 |
| HG01524 | HG03484 | NA11832 | NA19068 | NA20520 | HG02379 | HG03874 | NA18625 | NA19451 | NA21127 |
| HG01527 | HG03485 | NA11840 | NA19070 | NA20521 | HG02380 | HG03875 | NA18626 | NA19452 | NA21128 |
| HG01530 | HG03488 | NA11843 | NA19072 | NA20522 | HG02382 | HG03882 | NA18627 | NA19454 | NA21129 |
| HG01556 | HG03491 | NA11881 | NA19075 | NA20524 | HG02383 | HG03885 | NA18628 | NA19455 | NA21130 |
| HG01565 | HG03499 | NA11892 | NA19076 | NA20525 | HG02384 | HG03886 | NA18630 | NA19456 | NA21133 |
| HG01571 | HG03511 | NA11894 | NA19079 | NA20527 | HG02385 | HG03887 | NA18632 | NA19457 | NA21135 |
| HG01583 | HG03514 | NA11918 | NA19080 | NA20528 | HG02386 | HG03888 | NA18634 | NA19461 | NA21137 |
| HG01589 | HG03517 | NA11919 | NA19081 | NA20529 | HG02389 | HG03890 | NA18635 | NA19462 | NA21141 |
| HG01595 | HG03518 | NA11920 | NA19082 | NA20532 | HG02390 | HG03894 | NA18636 | NA19463 | NA21142 |
| HG01596 | HG03520 | NA11931 | NA19083 | NA20534 | HG02391 | HG03895 | NA18637 | NA19466 | NA21143 |
| HG01605 | HG03539 | NA11932 | NA19084 | NA20536 | HG02392 | HG03896 | NA18638 | NA19467 | NA21144 |
| HG01606 | HG03547 | NA11992 | NA19085 | NA20538 | HG02394 | HG03897 | NA18640 | NA19468 | NA24143 |
| HG01615 | HG03548 | NA11994 | NA19086 | NA20539 | HG02395 | HG03899 | NA18641 | NA19471 | NA24149 |
| HG02396 | HG03902 | NA18642 | NA19472 | NA24385 |  |  |  |  |  |


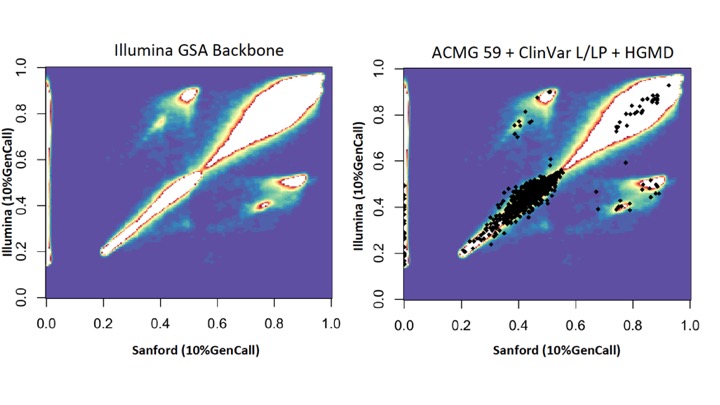


**Figure S1.** Comparison of p10GC between Illumina (*y-axis*) and Sanford GSA (*x-axis*) cluster files. *Left panel* shows all data and *right panel* shows ACMG59 ClinVar pathogenic / likely-pathogenic assays overlaid as black squares on top of all data-points.

Study sample selection rationale

Large-scale genotyping and sequencing studies select large sample sizes and typically target a sample size of over 1000 (1-4). To determine the validity of variation at a frequency >1% in the general population, Lee S et. al. proposed a framework to calculate a sample size given a target variation rate in the general population (Table S3) (5). With a finite sample size *N*, variants that are rare may not be observed. Supposing the population MAF for the *j^th^* variant is *m_j_* and $\theta_{j}$ is the chance that the variant *j* is observed in the sample size *N*,

$\theta_{j}=1-{(1-m_{j})}^{2N}$

then the sample size required to observe the variant *j* with at least $\theta_{j}$ is

$$N=\frac{ln(1-\theta_{j})}{2ln(1-m_{j})}$$

**Table S3.** Minimum number of samples required for a 99.9% chance to detect variation at a given minor allele frequency (MAF)

| **MAF** | **Minimum *N*** |
| --- | --- |
| **0.1** | 33 |
| **0.01** | 344 |
| **0.001** | 3,453 |
| **0.0001** | 34,537 |

To have a 99.9% chance of detecting variants with a MAF between ~1% and ~10%, a sample set of 263 samples from the Coriell Institute were selected to be genotyped in triplicate on the GSA as well as resequenced with Illumina WGS technology. The minor allele frequency for each GSA assay included in the 1000 Genomes Phase 3 data defined the number of needed samples, and this number was close to the theoretical number computed using the method of Lee S et al.

Data, Datasets, and Data organization

Dataset samples for analytical validation of Global Screening Array (GSA) data were selected from the 1000 Genomes project – Phase 3 (258) and the personal genomes project (5). The integrated genotype data from 1KG was downloaded and evaluated for coverage. 1KG genotype data covered 594,364 GSA assays. We also downloaded deep sequenced WGS from 1KG (pWGS; >51x) to evaluate rWGS, 1KG, and GSA data (ftp://ftp.1000genomes.ebi.ac.uk/vol1/ftp/phase3/data) (Table S4). We resequenced 263 samples on the Illumina HiSeqX to an average depth of 37x generating 731 million 150 bp paired-end reads (22.3 TB).

**Table S4.** Summary of number of samples in different datasets

| **Dataset** | **Total number of samples** | **Overlap with our dataset** | **Genotyping technology** | **Acronym** |
| --- | --- | --- | --- | --- |
| **1000 Genomes Genotype Data – Phase 3** | 2,504 | 258 | WES, WGS, Array | 1KG |
| **1000 Genomes WGS data*** | 27 | 24 | WGS | pWGS |
| **Resequenced WGS** | 263 | 263 | WGS | rWGS |
| **GSA** | 789 (263 x 3) | 789 (263x3) | Array | GSA |

*ftp://ftp.1000genomes.ebi.ac.uk/vol1/ftp/phase3/data

The data for resequenced WGS (rWGS) and public WGS (pWGS) were organized on AWS S3 bucket (Figure S2). All rWGS data files were mapped to canonical identifiers and file names were changed accordingly. Of the 1,104 samples used to generate the cluster file for GSA, 72 samples were included among the 263 samples in our primary validation dataset.


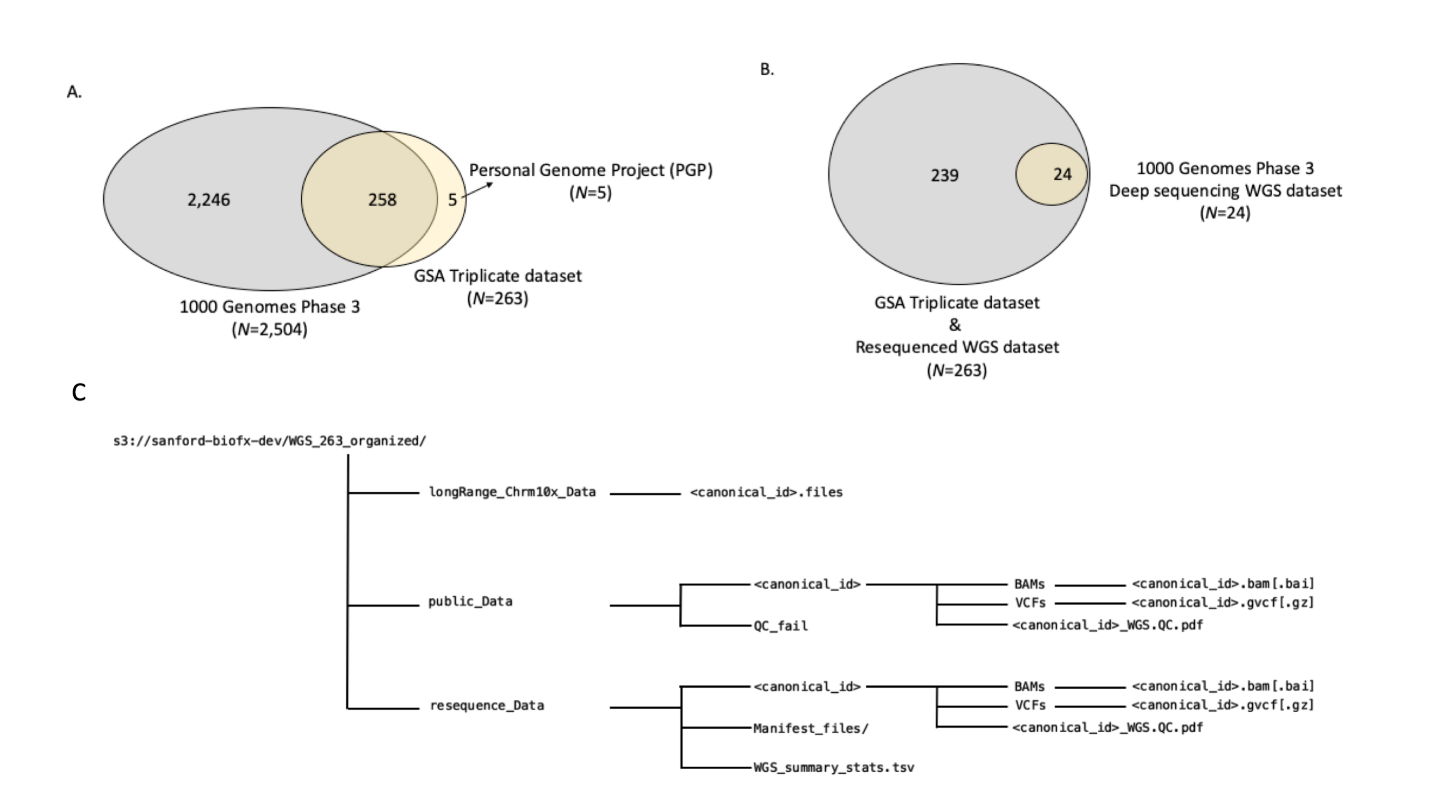


**Figure S2.** Datasets and their intersections with samples we selected from public benchmark databases. (A) Intersection of samples from 1000 Genomes Phase 3 benchmark dataset (2,504) and Personal Genomes Project (PGP; 5) with GSA validation samples (263; run in triplicate). (B) Intersection of 1000 Genomes Phase 3 deep sequencing WGS dataset (pWGS) relative to internally resequenced WGS dataset (rWGS) and GSA dataset. (C) The data-structure and organization of the WGS data archived on Sanford Imagenetics AWS S3 bucket (s3://sanford-biofx-dev).

WGS data

Whole Genome Sequence (WGS) data were either downloaded and reprocessed (pWGS) or resequenced (rWGS) and processed via GenomeNext and organized in an AWS S3 bucket (s3://sanford-biofx-dev). All WGS data and subsets are summarized in Table S4.

**Table S5.** WGS data subsets and summary

| **Datasets** | **Total samples** | **Pass QC** | **Fail QC** | **In_263** | **Data size** |
| --- | --- | --- | --- | --- | --- |
| **1000 Genomes (public data – pWGS)** | 27 | 24 | 3 | 24 | 3.9 TB |
| **WGS resequenced data (rWGS)** | 263 | 263 | 0 | 263 | 22.3 TB |

Quality metrics were extracted from the quality control (QC) PDF files from GenomeNext for reprocessed public data (n=24) and resequenced data (n=263) (Tables S4 and S5, respectively). The average depth of sequencing was computed using mapped read information and read-lengths for the resequenced data (n=263) (Figure S3) and the reprocessed public data (n=24) (Table S6). We also plotted the correlation of reported total processed reads vs. mapped reads to see if the relationship were linear and found it to be true (Figure S3). To check for completeness of all the data downloaded from GenomeNext, we generated a matrix of samples and all files expected under a given sample folder (e.g., BAM files, VCF files, etc.).


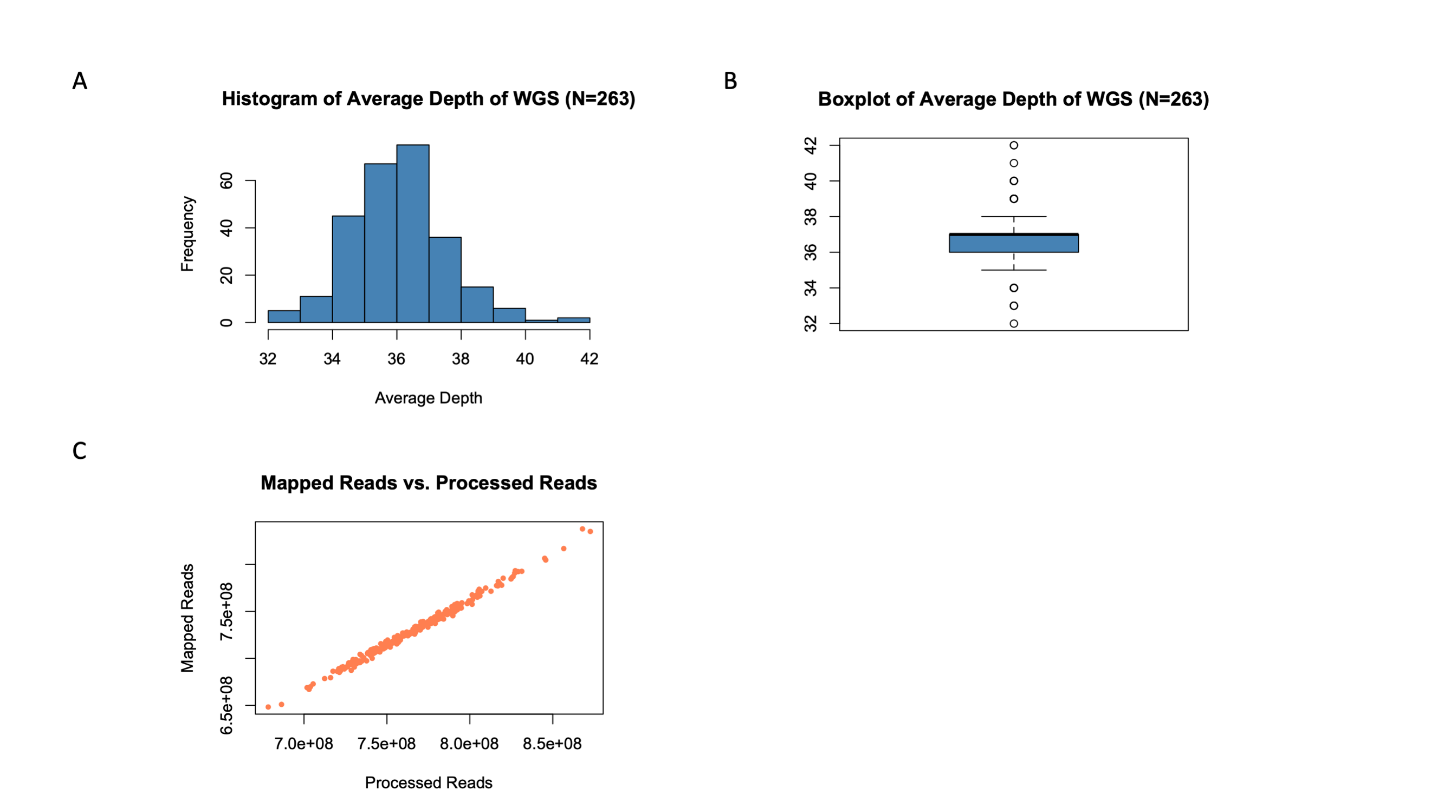


**Figure S3.** Quality control statistics of rWGS dataset (N=263). (A) A histogram of average read depth of coverage across HumanG1Kv37 for 263 resequenced WGS samples. (B) A box-plot of average read depth of coverage across HumanG1Kv37 for 263 resequenced WGS samples. (C) A scatter-plot of total number of reads mapped to reference sequence (HumanG1Kv37) as a function of total reads processed for alignment for each resequenced WGS sample.

**Table S6.** Public WGS (pWGS) from 1000 Genome Phase 3 downloaded and reprocessed data – summary statistics (QC pass samples)

| **Number** | **Samples** | **Processed** | **Discordant Reads** | **Mapq0 Reads** | **Unmapped Reads** | **Mapped** | **Average Depth** |
| --- | --- | --- | --- | --- | --- | --- | --- |
| **1** | HG02568 | 351,034,149 | 13,138,544 | 16,981,365 | 17,085,394 | 333,948,755 | 28 |
| **2** | HG03642 | 542,600,081 | 15,585,266 | 25,999,215 | 29,199,287 | 513,400,794 | 43 |
| **3** | HG01112 | 715,889,080 | 5,143,810 | 28,755,439 | 34,474,661 | 681,414,419 | 57 |
| **4** | HG00268 | 708,112,489 | 8,060,456 | 23,238,117 | 31,080,453 | 677,032,036 | 56 |
| **5** | HG03052 | 621,081,848 | 12,327,266 | 27,273,419 | 34,021,121 | 587,060,727 | 49 |
| **6** | NA18525 | 691,324,110 | 6,066,440 | 24,689,237 | 35,224,296 | 656,099,814 | 55 |
| **7** | NA20502 | 555,380,187 | 14,943,032 | 21,499,779 | 27,541,973 | 527,838,214 | 44 |
| **8** | NA18939 | 707,535,350 | 9,012,312 | 27,467,556 | 35,170,903 | 672,364,447 | 56 |
| **9** | HG01051 | 652,257,648 | 10,613,568 | 29,427,170 | 33,266,466 | 618,991,182 | 52 |
| **10** | HG00419 | 634,226,577 | 13,039,306 | 24,610,921 | 29,159,792 | 605,066,785 | 50 |
| **11** | HG03742 | 673,937,258 | 8,956,384 | 31,205,876 | 41,136,371 | 632,800,887 | 53 |
| **12** | NA19017 | 684,218,155 | 11,919,738 | 26,028,294 | 33,872,947 | 650,345,208 | 54 |
| **13** | HG01583 | 559,018,341 | 16,729,388 | 28,016,975 | 28,188,257 | 530,830,084 | 44 |
| **14** | HG00759 | 674,497,471 | 8,640,158 | 26,677,264 | 37,193,265 | 637,304,206 | 53 |
| **15** | NA19625 | 558,605,495 | 14,154,724 | 26,788,262 | 29,961,242 | 528,644,253 | 44 |
| **16** | HG01879 | 702,755,507 | 10,916,274 | 31,347,708 | 37,853,242 | 664,902,265 | 55 |
| **17** | HG01565 | 704,005,178 | 5,996,396 | 32,309,214 | 38,524,040 | 665,481,138 | 55 |
| **18** | HG01500 | 701,396,054 | 7,301,854 | 30,206,300 | 39,157,697 | 662,238,357 | 55 |
| **19** | HG01595 | 680,506,455 | 9,664,216 | 27,312,725 | 32,948,460 | 647,557,995 | 54 |
| **20** | HG00096 | 673,258,313 | 8,050,208 | 26,562,107 | 34,103,197 | 639,155,116 | 53 |
| **21** | NA19648 | 518,560,848 | 16,857,166 | 22,170,885 | 25,502,828 | 493,058,020 | 41 |
| **22** | NA20845 | 680,309,740 | 5,575,390 | 33,374,680 | 35,523,873 | 644,785,867 | 54 |
| **23** | HG02922 | 654,279,912 | 14,496,960 | 29,971,267 | 34,502,437 | 619,777,475 | 52 |
| **24** | HG03006 | 710,155,053 | 10,211,988 | 32,526,942 | 40,699,895 | 669,455,158 | 56 |
|  | Average Statistics | 639,789,387 | 10,725,035 | 27,268,363 | 33,141,337 | 606,648,050 | 51 |

samples failed QC = (NA19238, NA19239 – single reads; not paired end);

HG00276 is not on the list for PCR-free WGS; only low-coverage – but was processed by Databricks (HumanG1Kv37, GRCh38)

Mapped = Processed – Unmapped

Genome length = 3x10^9^

Read length – 250 bp

Supplementary Information – Nature paper states additional 2 trios were sequenced by PCR-free WGS, however (1 CEPH: NA12878, NA12891, NA12892, 1 YRI: NA19238, NA19239, NA19240) – All these are missing PCR-free data in the portal

**Table S7.** GenomeNext resequenced rWGS summary (N=263)

| **Statistic** | **Processed** | **Discordant** | **Mapq0** | **Unmapped** | **Mapped** | **Average Depth** |
| --- | --- | --- | --- | --- | --- | --- |
| **Minimum** | 678,435,694 | 1,382,636 | 26,482,204 | 29,491,105 | 648,242,190 | 32 |
| **1st Qu.** | 746,809,500 | 2,343,146 | 30,216,560 | 34,461,232 | 711,046,138 | 36 |
| **Median** | 766,996,302 | 3,191,934 | 31,927,018 | 36,287,654 | 730,666,603 | 37 |
| **Mean** | 767,540,183 | 3,799,360 | 31,937,208 | 36,312,190 | 731,227,993 | 36.57 |
| **3rd Qu.** | 786,304,491 | 4,793,391 | 33,625,400 | 38,189,484 | 748,822,998 | 37 |
| **Maximum** | 872,634,555 | 11,171,256 | 39,221,563 | 44,400,703 | 837,856,118 | 42 |

Principal Component Analysis (PCA)

Principal component analysis (PCA) is a standard technique to synthesize and summarize the main structural components of a data table containing measurements of different variables. PCA and Multi-dimensional reduction (MDR) techniques are frequently used in genotyping analyses to detect population structure (6), batch effects (DNA source, genotyping centers, chip versions, etc.), etc. Batch effects are highly undesirable in large-scale genotyping but are realistically unavoidable. These are best identified and minimized prior to any large study analyses. Given this challenge, we hypothesized that the PCA signature for super-populations should remain unaltered between different datasets in the absence of major technical variables that interfere with genotyping processes across datasets. To test this hypothesis, we used publicly available 1000 Genomes (Phase 3) genotype data from 2,504 samples, performed PCA analysis, and generated the super-population structure as a positive control. We compared these results to PCA plots generated using GSA genotyping data generated in-house on two independent datasets - ~ 700 samples (dataset 1) and ~ 900 samples (dataset 2).

Methods

Datasets

Publicly available 1000 Genomes data, Phase 3 (positive control) and Imagenetics GSA data (test data) were used in the PCA analysis.

Principal Component Analysis

Principal Component Analysis (PCA) was performed on 1000 Genome subsets and Imagenetics GSA genotyping data subsets using a C VCF-parsing program to read variant data into a sparse matrix (*Matrix*) in R (credit – bwlewis.github.io). Using the R package – *irlba*, the sparse data matrix was operated on to detect principal components, and using *threejs*, the 3D principal components were visualized*.* Additionally, we used PLINK to process the merged 258 sample 1KG VCF file and generated PCA data utilizing just the autosomal GSA assays and the 1KG genotypes. PLINK generates eigenvalues (amount of variation explained by each principal component), and eigenvectors (data points along different principal components / dimensions for each sample). We plotted the first two principal components and colored them based on the super population codes in the 1KG (Figure S4).


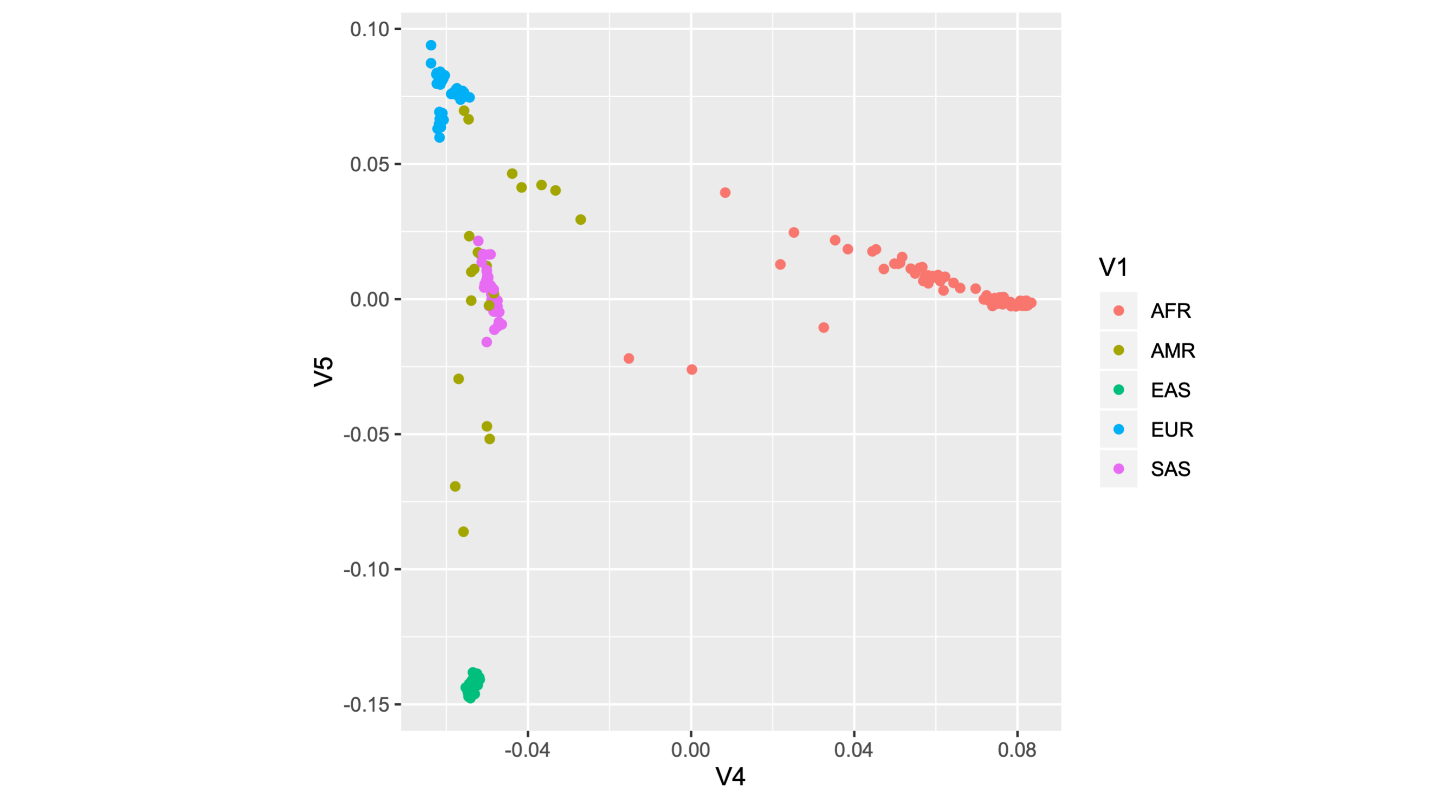


**Figure S4.** Principal component analysis (PCA) plotting the first two principle components generated using PLINK for 258 1000 Genomes Phase 3 genotype data using autosomal GSA assay loci.

PCA analysis testing – randomization experiment

To detect subtle systematic biases in GSA datasets, we randomly sub-sampled both the genomic loci (rows) and samples (columns) from Dataset 2. We synthetically created matrices with the following dimensions (rows x columns): (a) 10,000 x 500 (b) 10,000 x 900 (c) 600,000 x 900. Each matrix of a particular dimension was created independently three times, and PCA analysis was performed to check for deviation from the expected population structure observed in the 1KG.

Results

We analyzed the 1KG data by performing PCA on 2,504 individuals’ genotype data. These analyses were done on genotypes from all 1KG data, chromosome 1KG data, and 1KG data intersecting with GSA loci (all genotyped loci, chromosome 20, and GSA-specific loci). The super-population structure was reproducible and consistent across all three analyses of the 1KG and detected genotype differences between XX (females) and XY (males). We observed that 586,043 loci were sufficient to distinguish the 5 known major super-population groups. We considered these results to be our positive control for subsequent test analyses with GSA genotype data (Figure S5). To test the hypothesis that population structure would be reproducible if no other major variables distorted the expected structure, we applied PCA to the GSA data. Restricting GSA loci to autosomes (removal of sex chromosome and mitochondrial loci), our results were identical to those observed with the 1000 Genome Phase 3 genotype data (Figure S5). To validate this observation and ensure the absence of hidden, unobserved global variates, we performed a random sub-sampling experiment involving both genomic loci and samples (Figure S6). The results were consistently and reproducibly comparable to the positive control dataset and autosomal GSA datasets (Figure S7); this confirmed minimal technical artifacts in the GSA dataset.


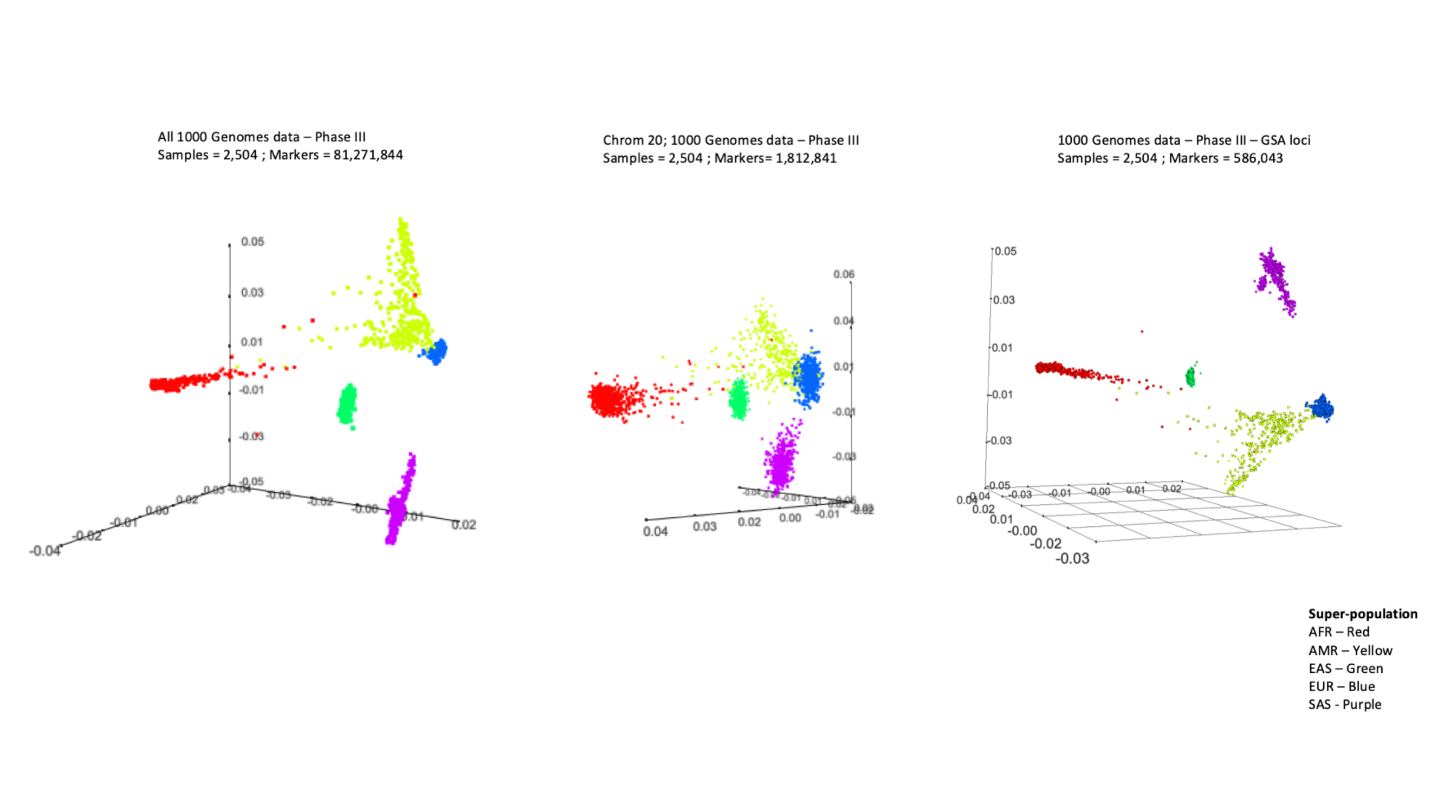


**Figure S5.** Principal component analysis (PCA) plotting the first three principal components and color coded by the five major super-population groups. *Left panel* plots the first 3 principal components for all 2,504 samples and all 1000 Genomes Phase 3 genotype data. *Middle panel* plots the first 3 principal components of chromosome 20 genotype data from 1000 Genomes Phase 3 for all 2,504 samples. *Right panel* plots the first 3 principal components of GSA assay loci genotype data from 1000 Genomes Phase 3 for all 2,504 samples.


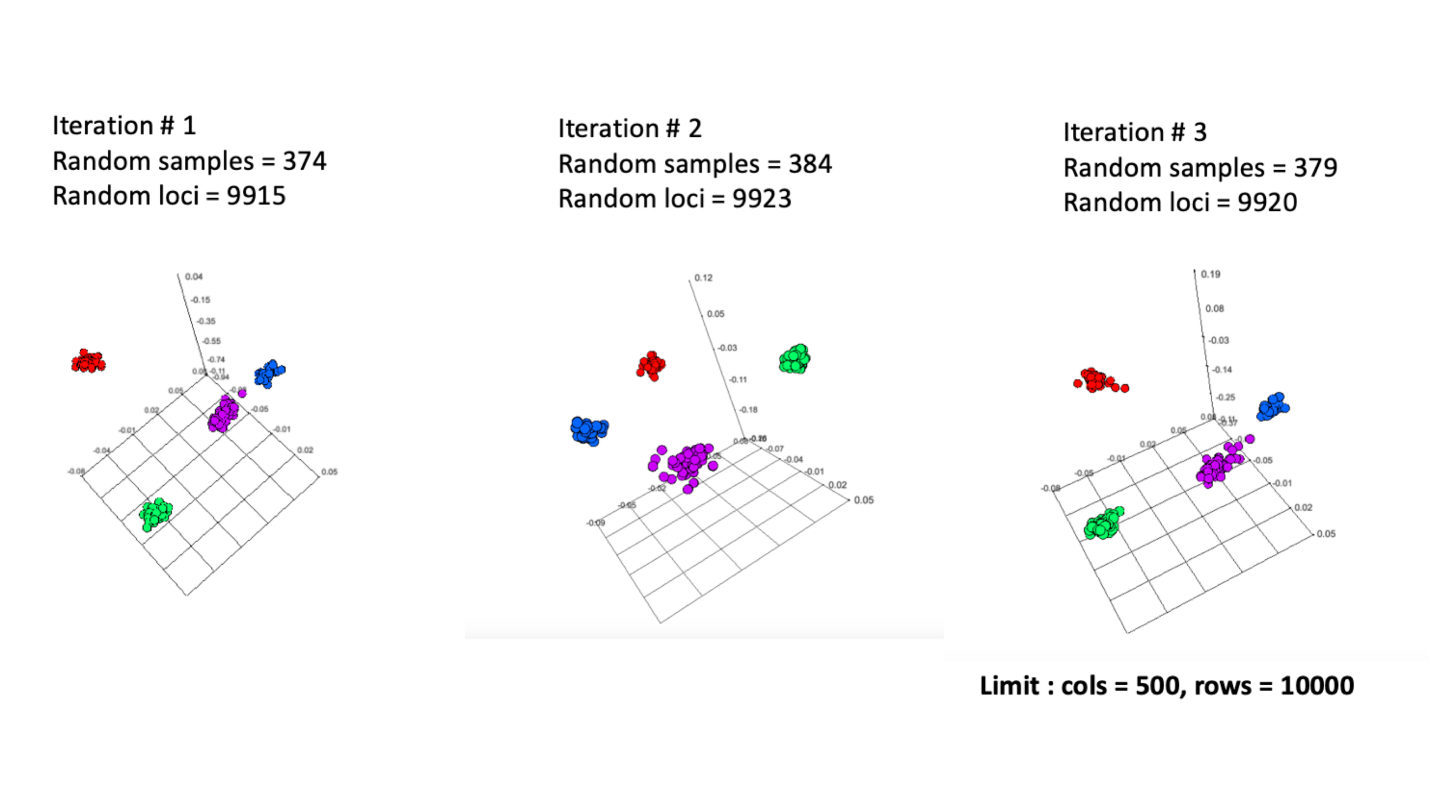


**Figure S6.** Principal component analysis (PCA) plotting the first three principal components of three random iterations of randomly selected samples and GSA loci with data from 1000 Genomes Phase 3 genotype data to show recapitulation of overall super-population grouping across iterations.


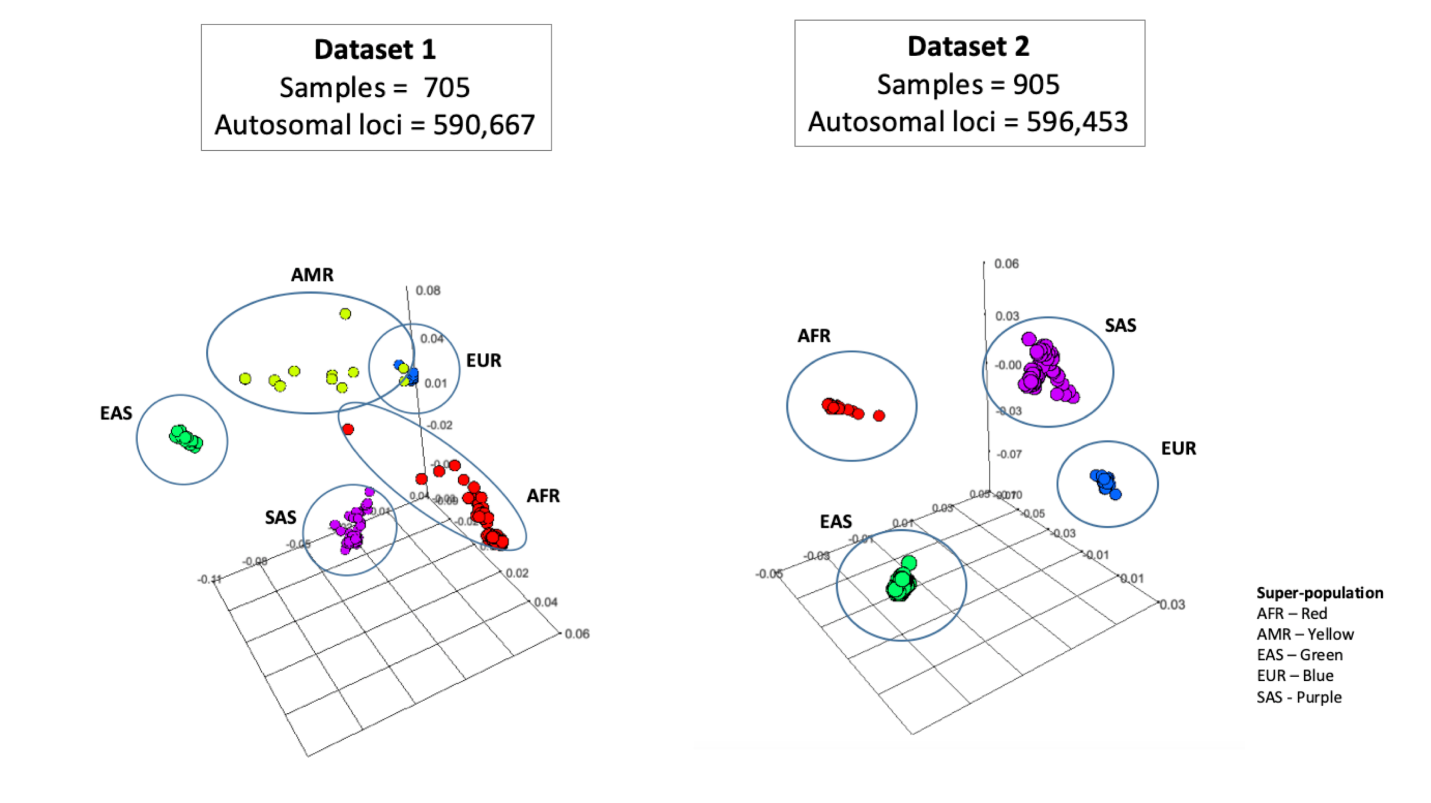


**Figure S7.** Principal component analysis (PCA) plotting the first three principal components of two GSA sample datasets – dataset 1 (n=705; *left panel*) and dataset 2 (n=905; *right panel*) using autosomal GSA loci; super-populations are grouped and color-coded.

R code

Code based on https://bwlewis.github.io/1000_genomes_examples/PCA.html

library(Matrix)

library(irlba)

library(threejs)

p = pipe("zcat /Users/703398698/Documents/Projects/GSA_Freq_validation/popln/mergedFile_batch1_genoCallRate_gt98_callRate_gt9975_mod.autosomes.VCF.Z | sed /^#/d | cut -f '10-' | /Users/703398698/Documents/Projects/GSA_Freq_validation/popln/parse_gsa | cut -f '1-2' ")

x = read.table(p, colClasses=c("integer","integer"), fill=TRUE, row.names=NULL)

gsa = sparseMatrix(i=x[,2], j=x[,1], x=1.0)

dim(gsa)

cm = colMeans(gsa)

p = irlba(gsa, nv=3, nu=3, tol=0.1, center=cm)

ids = readLines(pipe("zcat /Users/703398698/Documents/Projects/GSA_Freq_validation/popln/mergedFile_batch1_genoCallRate_gt98_callRate_gt9975_mod.autosomes.VCF.Z | sed -n /^#CHROM/p | tr '\t' '\n' | tail -n +10"))

ped = read.table(url("ftp://ftp.1000genomes.ebi.ac.uk/vol1/ftp/technical/working/20130606_sample_info/20130606_g1k.ped"),sep="\t",header=TRUE,row.names=2)[ids,6,drop=FALSE]

pop = read.table("ftp://ftp.1000genomes.ebi.ac.uk/vol1/ftp/phase3/20131219.populations.tsv",sep="\t",header=TRUE)

pop = pop[1:26,]

super = pop[,3]

names(super) = pop[,2]

super = factor(super)

ped$Superpopulation = super[as.character(ped$Population)]

N = length(levels(super))

scatterplot3js(p$u, col=rainbow(N)[ped$Superpopulation], size=0.5)

library(Matrix)

library(irlba)

library(threejs)

p = pipe("zcat /Users/703398698/Documents/Projects/GSA_Freq_validation/popln/mergedFile_batch2_mod_genoCallRate_gt98_callRate_gt9975_subNoCallwithRef.autosomes.VCF.Z | sed /^#/d | cut -f '10-' | /Users/703398698/Documents/Projects/GSA_Freq_validation/popln/parse_gsa | cut -f '1-2' ")

x = read.table(p, colClasses=c("integer","integer"), fill=TRUE, row.names=NULL)

gsa = sparseMatrix(i=x[,2], j=x[,1], x=1.0)

dim(gsa)

cm = colMeans(gsa)

p = irlba(gsa, nv=3, nu=3, tol=0.1, center=cm)

ids = readLines(pipe("zcat /Users/703398698/Documents/Projects/GSA_Freq_validation/popln/mergedFile_batch2_mod_genoCallRate_gt98_callRate_gt9975_subNoCallwithRef.autosomes.VCF.Z | sed -n /^#CHROM/p | tr '\t' '\n' | tail -n +10"))

ped = read.table(url("ftp://ftp.1000genomes.ebi.ac.uk/vol1/ftp/technical/working/20130606_sample_info/20130606_g1k.ped"),sep="\t",header=TRUE,row.names=2)[ids,6,drop=FALSE]

pop = read.table("ftp://ftp.1000genomes.ebi.ac.uk/vol1/ftp/phase3/20131219.populations.tsv",sep="\t",header=TRUE)

pop = pop[1:26,]

super = pop[,3]

names(super) = pop[,2]

super = factor(super)

ped$Superpopulation = super[as.character(ped$Population)]

N = length(levels(super))

scatterplot3js(p$u, col=rainbow(N)[ped$Superpopulation], size=0.5)

p = pipe("zcat /Users/703398698/Desktop/GSA/pca/gsa_b1_out_mod_auto.VCF.Z | sed /^#/d | cut -f '10-' | /Users/703398698/Documents/Projects/GSA_Freq_validation/popln/parse_gsa | cut -f '1-2' ")

x = read.table(p, colClasses=c("integer","integer"), fill=TRUE, row.names=NULL)

gsa = sparseMatrix(i=x[,2], j=x[,1], x=1.0)

dim(gsa)

cm = colMeans(gsa)

p = irlba(gsa, nv=3, nu=3, tol=0.1, center=cm)

ids = readLines(pipe("zcat /Users/703398698/Desktop/GSA/pca/gsa_b1_out_mod_auto.VCF.Z | sed -n /^#CHROM/p | tr '\t' '\n' | tail -n +10"))

ped = read.table(url("ftp://ftp.1000genomes.ebi.ac.uk/vol1/ftp/technical/working/20130606_sample_info/20130606_g1k.ped"),sep="\t",header=TRUE,row.names=2)[ids,6,drop=FALSE]

pop = read.table("ftp://ftp.1000genomes.ebi.ac.uk/vol1/ftp/phase3/20131219.populations.tsv",sep="\t",header=TRUE)

pop = pop[1:26,]

super = pop[,3]

names(super) = pop[,2]

super = factor(super)

ped$Superpopulation = super[as.character(ped$Population)]

N = length(levels(super))

scatterplot3js(p$u, col=rainbow(N)[ped$Superpopulation], size=0.5)

p = pipe("zcat /Users/703398698/Desktop/GSA/pca/gsa_b2_out_mod_auto.VCF.Z | sed /^#/d | cut -f '10-' | /Users/703398698/Documents/Projects/GSA_Freq_validation/popln/parse_gsa | cut -f '1-2' ")

x = read.table(p, colClasses=c("integer","integer"), fill=TRUE, row.names=NULL)

gsa = sparseMatrix(i=x[,2], j=x[,1], x=1.0)

dim(gsa)

cm = colMeans(gsa)

p = irlba(gsa, nv=3, nu=3, tol=0.1, center=cm)

ids = readLines(pipe("zcat /Users/703398698/Desktop/GSA/pca/gsa_b2_out_mod_auto.VCF.Z | sed -n /^#CHROM/p | tr '\t' '\n' | tail -n +10"))

ped = read.table(url("ftp://ftp.1000genomes.ebi.ac.uk/vol1/ftp/technical/working/20130606_sample_info/20130606_g1k.ped"),sep="\t",header=TRUE,row.names=2)[ids,6,drop=FALSE]

pop = read.table("ftp://ftp.1000genomes.ebi.ac.uk/vol1/ftp/phase3/20131219.populations.tsv",sep="\t",header=TRUE)

pop = pop[1:26,]

super = pop[,3]

names(super) = pop[,2]

super = factor(super)

ped$Superpopulation = super[as.character(ped$Population)]

N = length(levels(super))

scatterplot3js(p$u, col=rainbow(N)[ped$Superpopulation], size=0.5)

Sample DNA contamination estimation and detection

The GSA measures the relative intensity of fluorescently labeled probes associated with A and B alleles. After normalizing the intensities, the Illumina software assigns a genotype for each genomic locus assayed as AA (homozygous A, B-allele frequency (BAF) =~ 0; absence of B allele), or AB (heterozygous, BAF =~ 0.5; 50% B allele), or BB (homozygous B, BAF =~ 1; 100% B-allele). A missing genotype is assigned in positions with intensities outside the expected clusters. In addition, the output also contains the estimated abundance of the B allele called the B allele frequency (BAF) and the log-likelihood ratio (LLR) of observed signal intensity at a given locus relative to expected signal intensity.

Regression-based method to detect sample contamination

The regression-based method detects contamination by identifying shifts in signal between the expected and the B allele frequency in sites called as homozygous^[[1]](#footnote-1)^*. Consider a sample with the AA genotype at a particular genomic location whose DNA sample is contaminated. As the population frequency of the minor allele (B allele) increases, the sample is increasingly likely to be contaminated with the B allele (7). In absence of sample contamination, we expect BAF values close to 0, 0.5, and 1 for genotypes AA, AB and BB, respectively; however, in presence of contamination, the expected BAF scales with the level of contamination.

$$E\left[ BAF \right| g=AA; \propto, p_{B}]= \propto p_{B}$$

and, $E\left[ BAF \right| g=BB; \propto, p_{A}]= 1 - \propto p_{A}$

where $p_{A}$ and $p_{B}$ are the population frequencies of A and B, $\propto$ is the contamination level (fraction; range 0 - 1).

This gives a linear regression model for BAF

$$BAF= \gamma+ \alpha p+\tau I\left( g=AA \right)+\varepsilon$$

Where $\gamma$ is the intercept, $p=\left\{ \begin{aligned} p_{B}, if g=AA \\ {-p}_{A}, if g=BB \end{aligned} \right.$ , $\tau$ is the difference between expected BAF for AA and BB genotypes, and $\varepsilon$ is the normally distributed error term. The test for contamination can be evaluated by testing the null hypothesis that $\alpha$=0 against the one-sided alternative that $\alpha>0$.

In addition to using publicly available databases that record population frequency of the A and B alleles, allele frequencies can also be estimated from the Illumina genotyping cluster file that we generated from the 1,104 samples from diverse population groups. Using the cluster file, we estimated the minor allele frequency (MAF) $f$

$$f=\frac{\left( 2*mC \right)+hC}{(2*T)}$$

Where $mC$ is the minor allele genotype count, $hC$ is the heterozygous genotype count, and $T$ is the total count of all genotypes at a particular genotyping locus. Following this derivation of the MAF and given the minor allele frequency and given that the genotype is AA, it is feasible (i) to calculate the probability that B is the minor allele and (ii) to compute the expected BAF.

$$\Pr\left( B is minor allele \right|g=AA and f)= \frac{Pr(B is minor allele, g=AA and f)}{\Pr\left( B is minor allele, g=AA and f \right)+Pr(A is minor allele, g=AA and f)}$$

$$\Pr\left( B is minor allele \right|g=AA and f)=\frac{{(1-f)}^{2}}{{(1-f)}^{2}+f^{2}}$$

$$E\left[ BAF \right|g=AA; \alpha, f]=\alpha\frac{f(1-f)}{{(1-f)}^{2}+f^{2}}$$

where $\alpha$ is level of contamination. The linear regression model can be updated to:

$$BAF= \gamma+ \alpha f+\tau I\left( g=AA \right)+\varepsilon$$

Jun, G et. al. show that the linear regression model to calculate BAF using minor allele frequency is identical to population frequency-based computation; therefore, this method allows detection of contamination using genotypes encoded either as AA, AB or BB, and removes the necessity to decode the A and B alleles to their respective reference genome nucleotides (A, C, G, or T). This method also allows for implementation of sample contamination detection in early stages of data analysis and serves as a sample contamination detection quality control step. Using linear regression analysis of known and predicted DNA contamination, we generated a standard curve for a continuum of contamination between DNA samples from genetically unrelated individuals and between DNA samples from genetically related individuals (Figure S8). Additionally, we evaluated the relationship between sample contamination and GSA call-rate and heterozygous to homozygous call ratio (Figure S9).


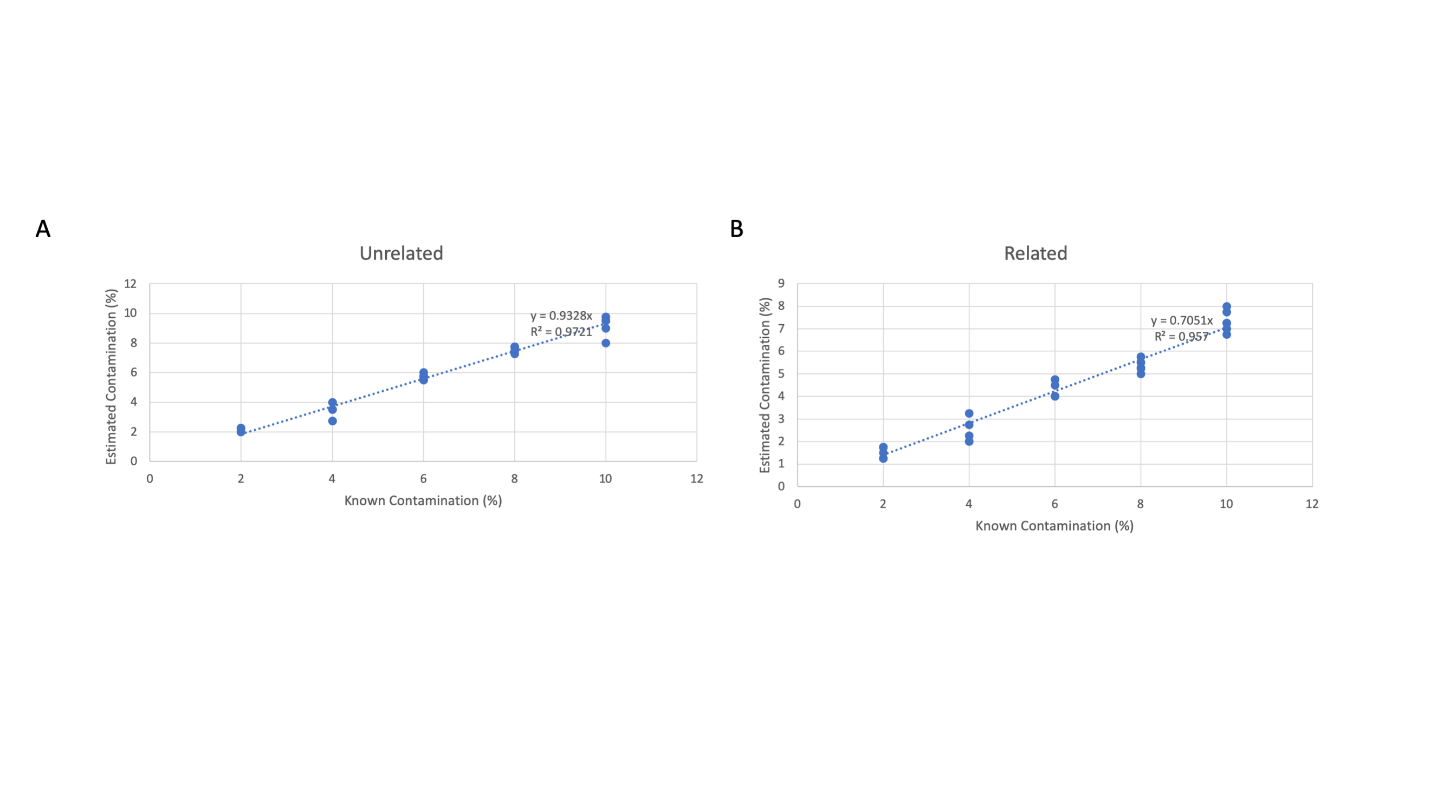


**Figure S8.** Standard linear regression analysis of estimated sample contamination (%) as a function of known sample contamination (%). Each known level of sample contamination (2% - 10%) data was generated in triplicate. (A) Estimation of sample contamination under the assumption that primary sample DNA and contaminant sample DNA are from unrelated individuals. (B) Estimation of sample contamination under the assumption that primary sample DNA and contaminant sample DNA are from related individuals.


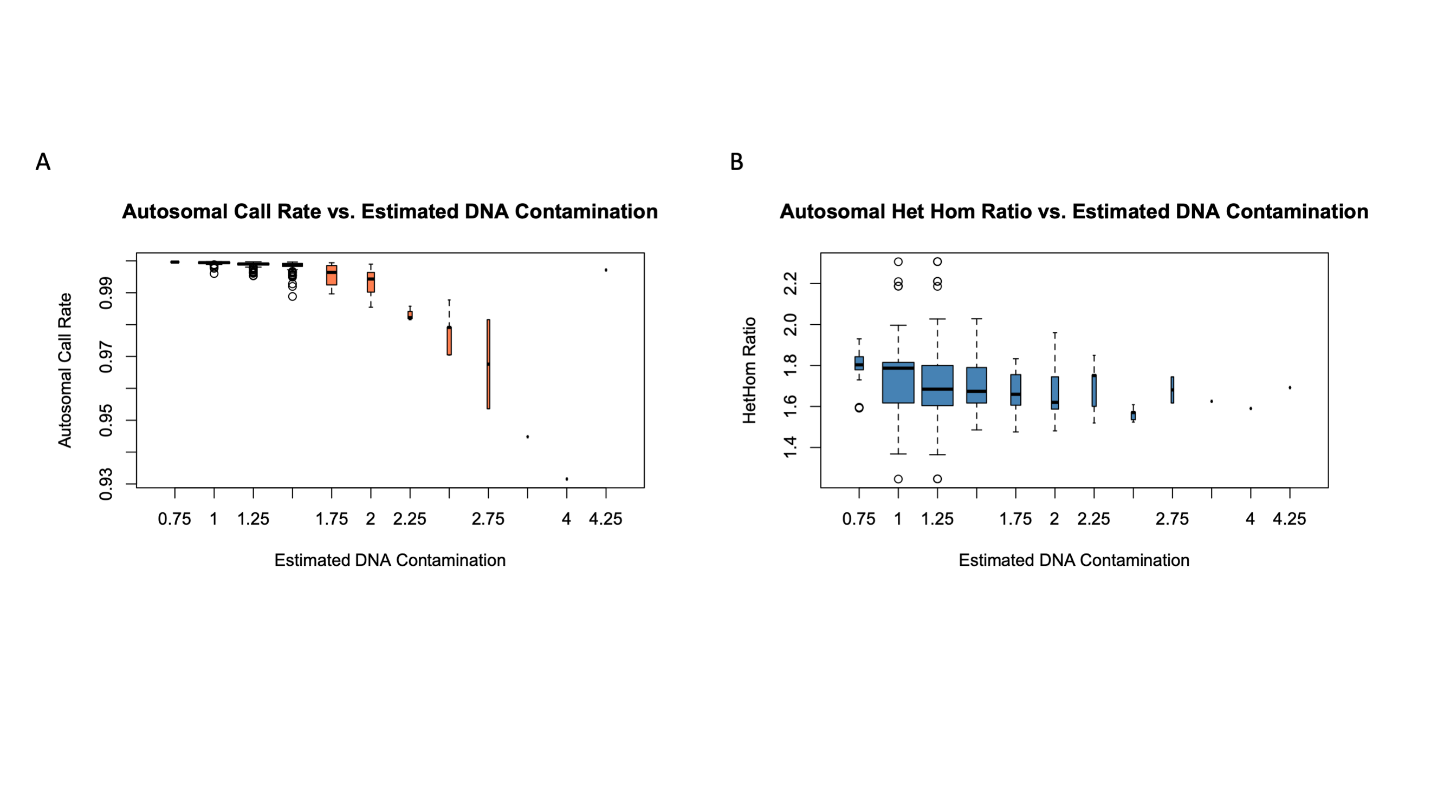


**Figure S9.** A box-plot analysis and comparison of autosomal call rate (*panel A*) and heterozygous calls / homozygous calls ratio (*panel B*) to estimated DNA sample contamination.

GSA quality control analyses

The quality control (QC) for the GSA was performed at multiple levels including (a) the BeadChip Array, (b) sample replicate, and (c) the VCF statistics.

Quality control for BeadChip Array

The QC for GSA data at the level of BeadChip was evaluated for (a) call rate, (b) p10GC, and (c) sample contamination. Each BeadChip was analyzed and the data was plotted using the R-code (below) to detect outlier samples (Figure S10).


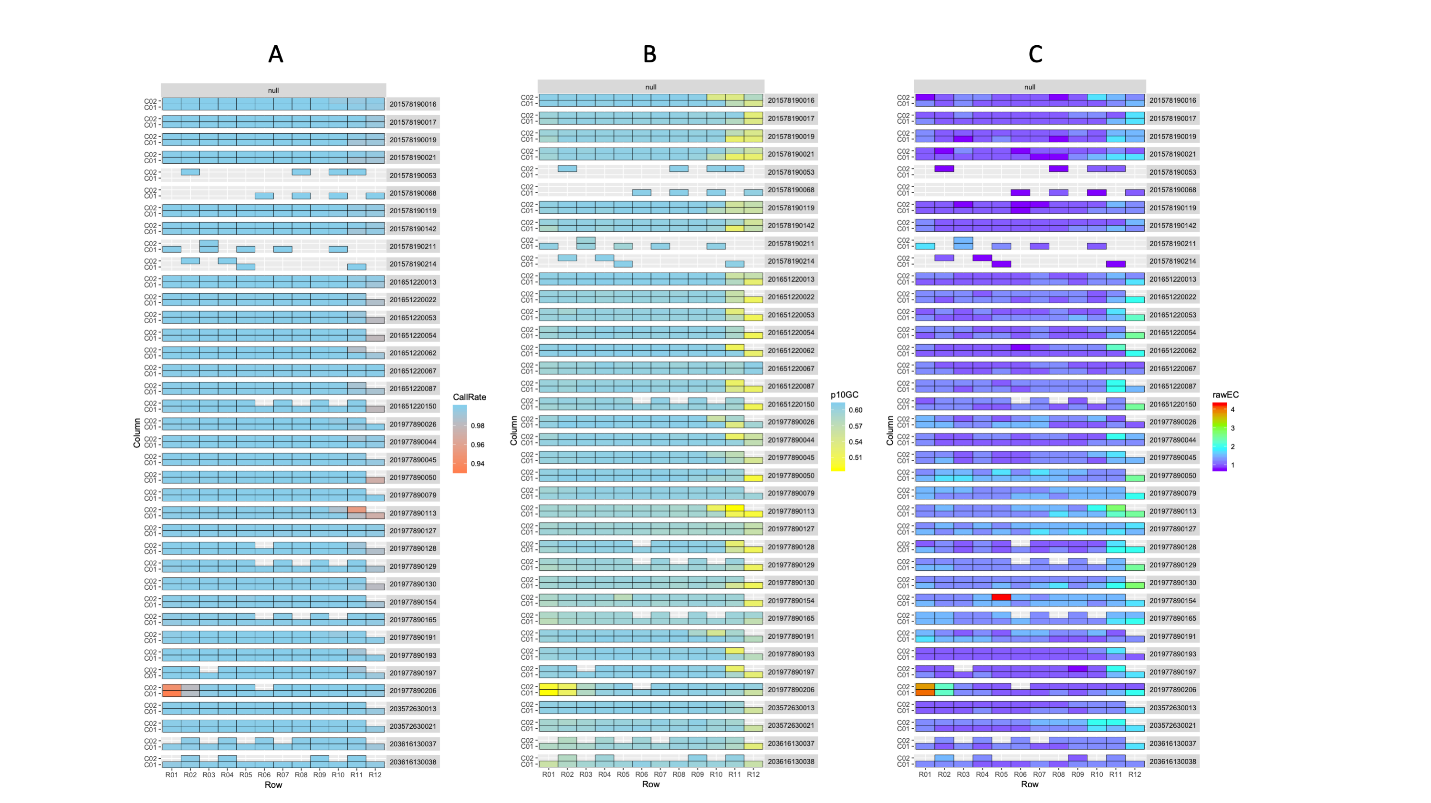


**Figure S10.** Heatmaps of BeadChip array quality control analysis of call-rate (*left*), p10GC (*middle*), and estimated DNA contamination (*right*). The color gradient scales for the three panels are as follows: call-rate (*orange* < 0.94 – *blue* > 0.99), p10GC (*yellow* < 0.50 – *blue* > 0.60) and estimated DNA contamination (rainbow gradient: *purple* ~ 1%, *blue* ~ 2%, *green* ~3%, *orange* / *red* ~ > 4%).

df<-read.table("/Users/703398698/Documents/Projects/GSA_replicates/GSA_WGS_263/dataset_263_master_QC.csv", sep="," , header=T)

ggplot(df, aes(Row, Column, fill=CallRate)) + geom_tile(colour="black") + facet_grid(BeadChip~Group) + scale_fill_gradient(low="coral", high="skyblue") + theme(strip.text.y=element_text(angle=360))

df<-read.table("/Users/703398698/Documents/Projects/GSA_replicates/GSA_WGS_263/dataset_263_master_QC.csv", sep="," , header=T)

ggplot(df, aes(Row, Column, fill=CallRate)) + geom_tile(colour="black") + facet_grid(BeadChip~Group) + scale_fill_gradient(low="yellow", high="skyblue") + theme(strip.text.y=element_text(angle=360))

df<-read.table("/Users/703398698/Documents/Projects/GSA_replicates/GSA_WGS_263/dataset_263_master_QC.csv", sep="," , header=T)

ggplot(df, aes(Row, Column, fill=rawEC)) + geom_tile(colour="black") + facet_grid(BeadChip~Group) + scale_fill_gradient(colours=rev(rainbow(4))) + theme(strip.text.y=element_text(angle=360))

Quality control for GSA triplicate samples

The QC for GSA triplicate samples were evaluated in tandem as R1, R2, and R3, respectively, across the quality control metrics of (a) call rate, (b) p10GC, (c) sample contamination, (d) no genotype calls, and (e) heterozygous genotype / homozygous genotype ratio (Figure S11). Additionally, replicate sample outliers were detected by applying a Z-score method to call rate data (Figure S12). The R-code used to generate these plots is shown below:

ds <- read.table("/Users/703398698/Desktop/GSA/Supplementary_Data/triplicate_VCF/merged_genomeStudio_VCFStats.csv", sep=",", header=T)

ggplot(ds, aes(Dataset, Sample, fill=withGenotypes/TotalGSAAssays)) + geom_tile(color="gray")+scale_fill_gradient(low="coral", high="skyblue") + labs(title="Call Rate \n", fill="Call Rate\n") + theme(axis.text.y=element_text(size=3))

ggplot(ds, aes(Dataset, Sample, fill=withGenotypes/TotalGSAAssays)) + geom_tile(color="gray")+scale_fill_gradientn(colours=rainbow(4))+ labs(title="Call Rate \n", fill="Call Rate\n") + theme(axis.text.y=element_text(size=3))

ggplot(ds, aes(Dataset, Sample, fill=p10GC)) + geom_tile(color="gray")+scale_fill_gradientn(colours=rainbow(4)) + labs(title="p10GC \n", fill="p10GC\n") + theme(axis.text.y=element_text(size=3))

ggplot(ds, aes(Dataset, Sample, fill=rawEC)) + geom_tile(color="gray")+scale_fill_gradientn(colours=rev(rainbow(4))) + labs(title="Estimated DNA Contamination \n", fill="Est Contam\n") + theme(axis.text.y=element_text(size=3))

ggplot(ds, aes(Dataset, Sample, fill=NoGenotypes)) + geom_tile(color="gray")+scale_fill_gradient(low="skyblue", high="coral") + labs(title="No Genotype Calls \n", fill="No Genotype Calls\n") + theme(axis.text.y=element_text(size=3))

ggplot(ds, aes(Dataset, Sample, fill=auto_het_SNV/auto_homalt_SNV)) + geom_tile(color="gray")+scale_fill_gradientn(colours=rainbow(4)) + labs(title="Het / Hom Ratio\n", fill="Het / Hom Ratio\n") + theme(axis.text.y=element_text(size=3))

tmp<-cbind(df,'Z_score'=(df$Autosome_data-599557)/sqrt(var(df$Autosome_data)))

plot(tmp$Z_score, pch=16, cex=1, col="steelblue", ylab="Z-Score")

abline(h=-4, lty=2)

abline(h=-2, lty=2)

abline(h=0, lty=1, col="red")


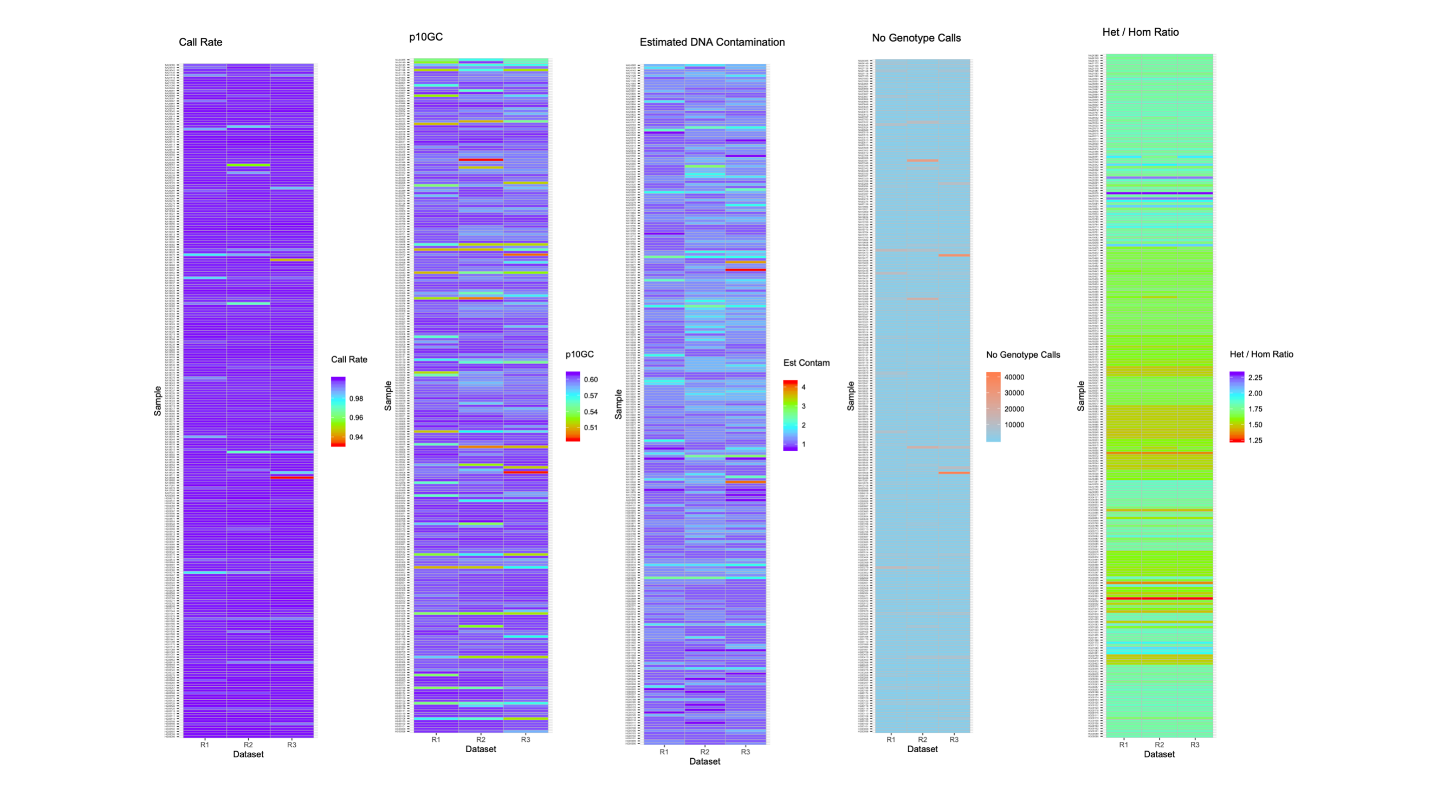


**Figure S11.** Heatmaps of reproducibility quality control analysis using replicate data as measured by call rate, p10GC, estimated DNA contamination, number of assays with no genotype calls, and heterozygote to homozygote ratio. The color gradient scales for these five heatmaps are as follows: rainbow gradient for call rate (*purple* > 0.99 – *red* < 0.94), rainbow gradient for p10GC (*purple >*0.60 – *red <* 0.5), estimated DNA contamination (*purple* < 1% – *red* > 4%), no genotype calls (*blue* < 166,000 – *orange* > 400,000), and heterozygote / homozygote ratio (*purple* > 2.25 – *red* < 1.25) respectively.


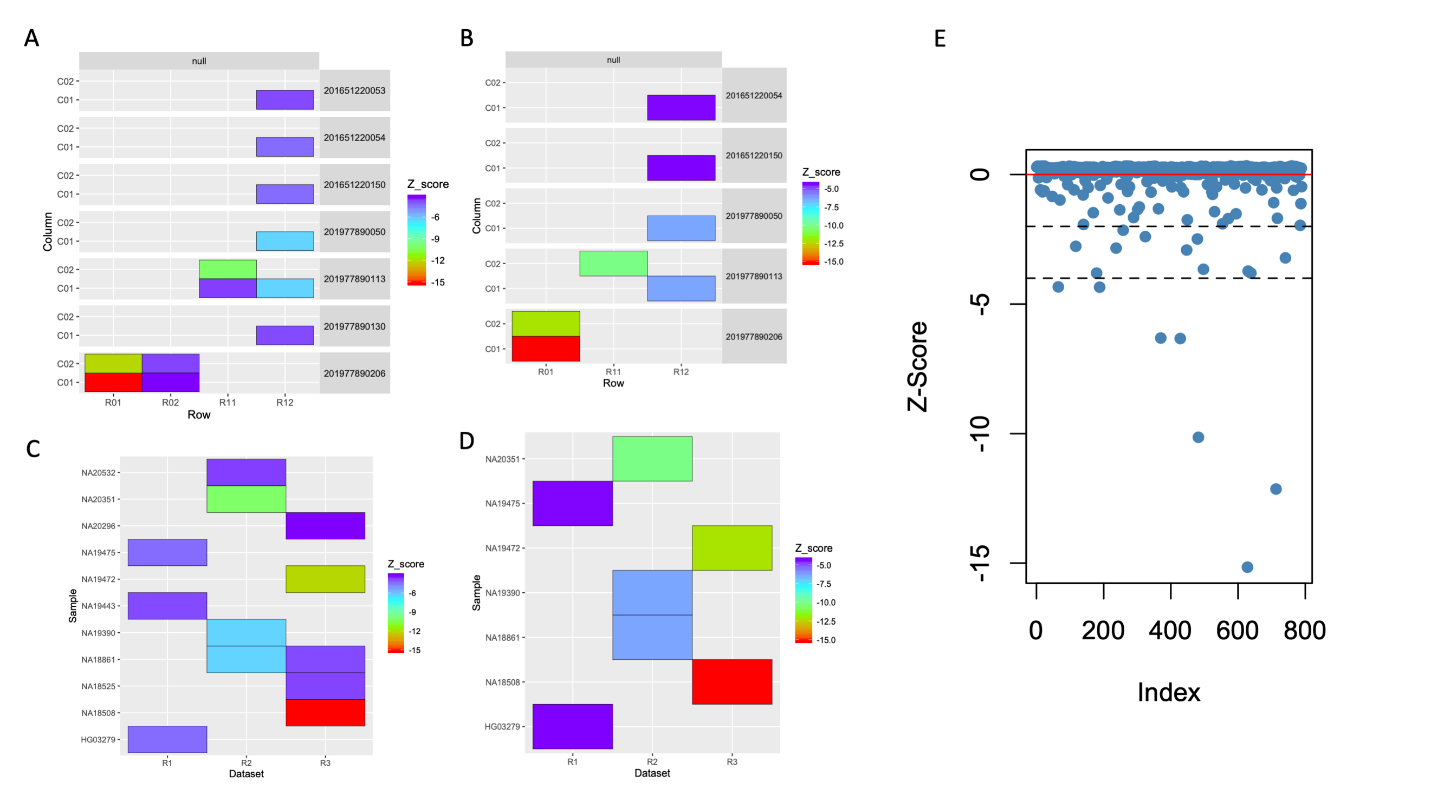


**Figure S12.** Quality control of GSA data by detecting triplicate samples with outlier call rates. *Panels A and B:* The outlier call rate detected along the edges of BeadChip array (rows 11 and 12); rows 01 and 02 samples were detected as samples with DNA contamination. *Panel A* uses a stringent Z-score threshold (< -3), whereas *panel B* uses a lenient Z-score threshold (< -4). *Panels C* and *D* show outlier detection across the three replicates; they parallel *Panels A* and *B. Panel E* plots Z-scores as a function of GSA triplicate sample data index. The *dashed-lines* depict lenient (<-4) and stringent (< -3) thresholds.

Quality control of GSA triplicate VCF files

We evaluated 236 samples’ triplicate VCF files for quality. Summary statistics for each replicate VCF file were generated using *rtg* (a program for VCF statistics) and plotted relative to each other to detect correlation (Figure S13). It was run as follows: *rtg VCFstats [sampleid_replicate#.vcf.gz]*. The summary of this analysis is the file *GSA_236_triplicates_VCFSummary.xls.*


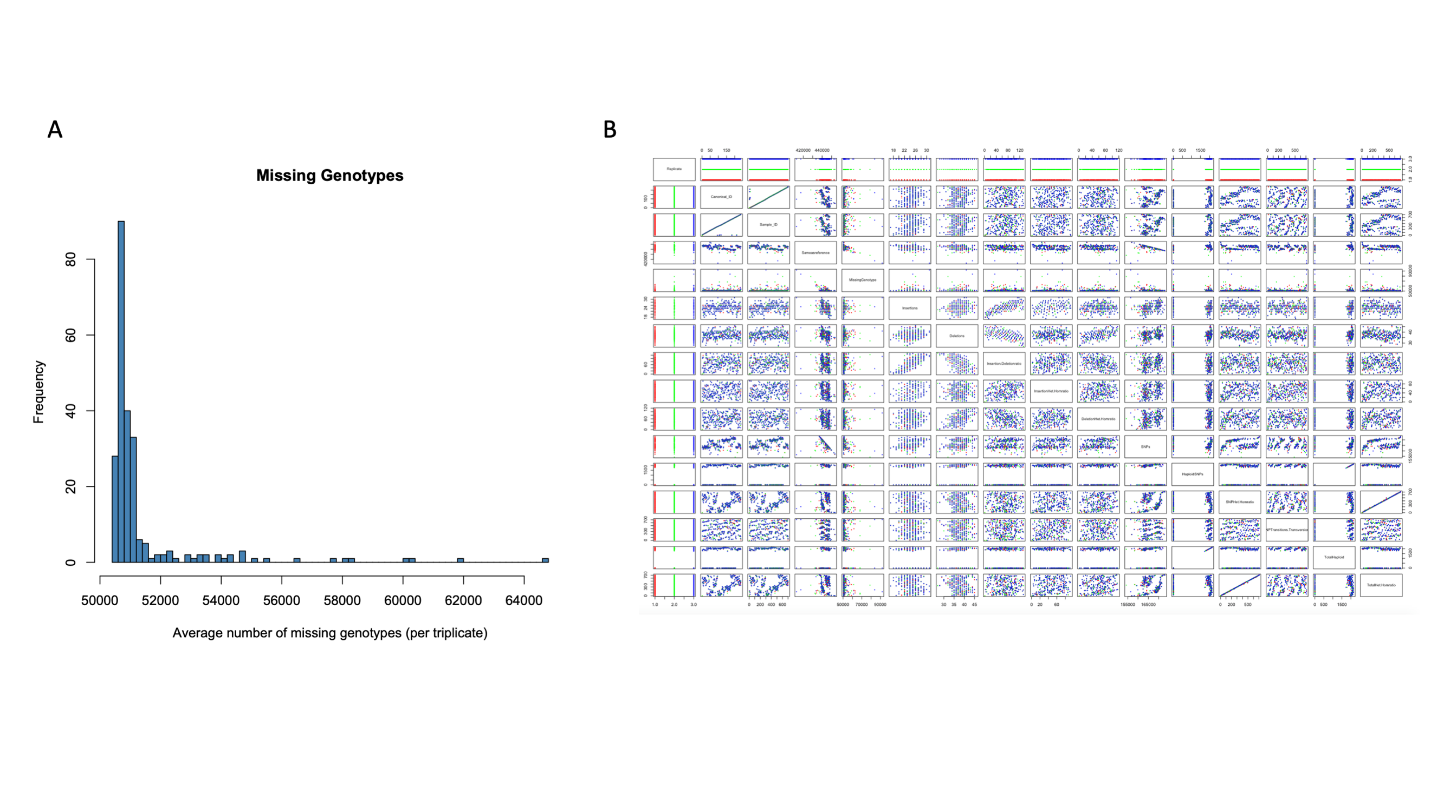


**Figure S13.** Quality control analysis of GSA call rate and GSA VCF statistics. (A) A histogram of the number of missing genotypes per sample for all GSA triplicate data. (B) All-vs-all scatter-plot analysis of GSA VCF statistics for all triplicate data.

Transitions and transversions

The GSA SNV assays were stratified by the type of nucleotide change, i.e., transition (purine to purine and pyrimidine to pyrimidine) or transversion (purine to pyrimidines and vice versa). Transversions performed poorer than transitions across the performance metrics of sensitivity, specificity, PPV, and concordance (Figure S14 A). Performance metrics of low-complexity regions are shown in Figure S14 B.


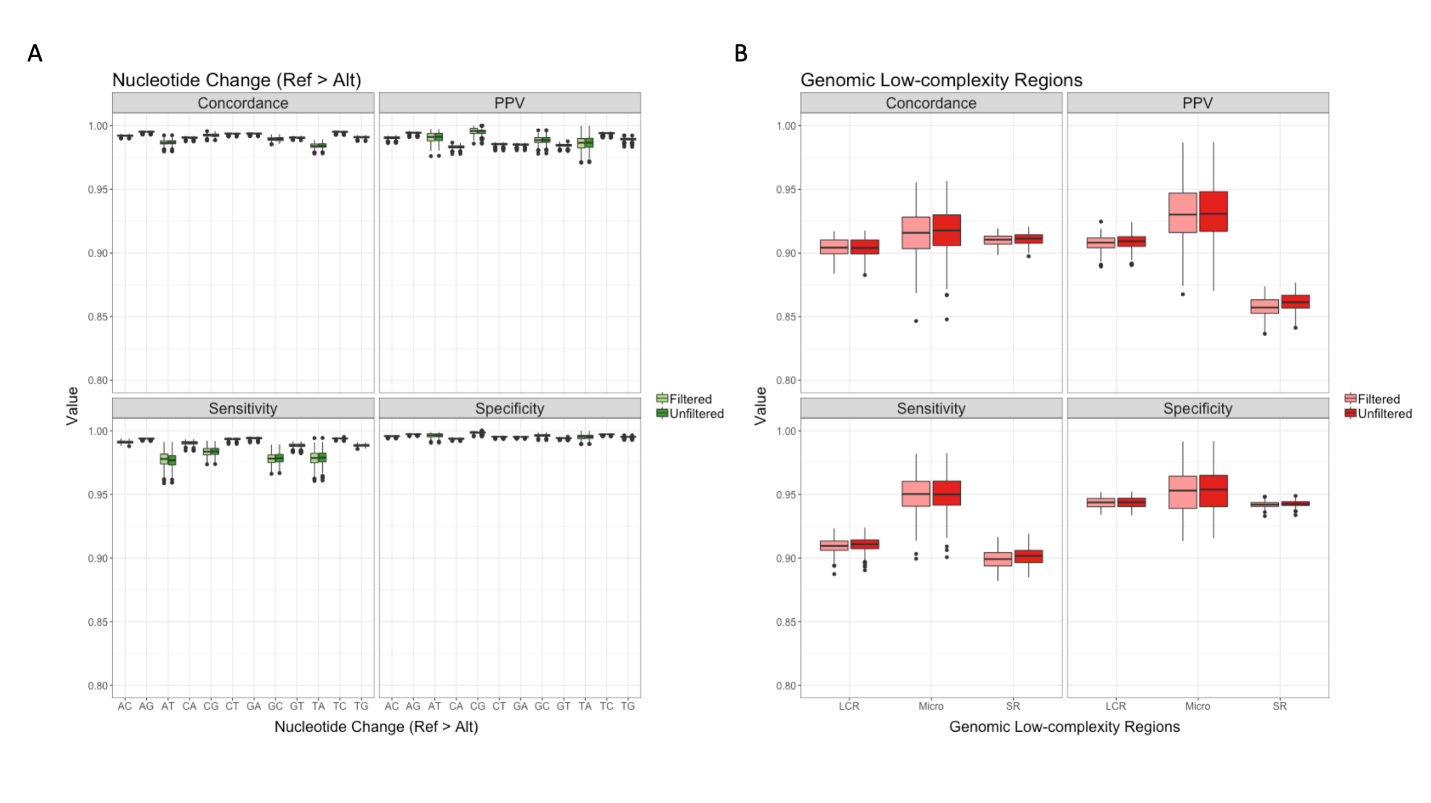


**Figure S14.** A box-plot analysis of performance metrics of GSA vs 1KG benchmark dataset when assays are classified according to (A) type of single-nucleotide change (transition (TNS), transversion (TVS)). All 12 possible nucleotide changes are shown along the x-axis. The first nucleotide is the reference allele, and the second nucleotide is the variant allele. Data filtered and samples removed for low-quality are shown in *light green*. (B) Interrogation of a low-complexity genomic regions. The data filtered and samples removed for low-quality are shown in *light red*.

Transitions and transversions with special focus on complementary transversions

Transitions interchange bases of similar shape, whereas transversions interchange bases of differing structure, i.e., one-ring pyrimidines (C, T) for two-ring purines (A, G) or vice versa (Figure S17). Given 4 nucleotide bases (A,C,G,T), there are a total of 16 possible interchanges (4x4). 8 transitions and 8 transversions. However, 4 “transitions” of the 8 possible transitions are unobserved due to self-interchange (e.g., A>A). This leaves 12 total interchanges: 4 transitions and 8 transversions (Figure S15). The 4 possible transitions are: A>G, G>A, C>T, and T>C. The 8 possible transversions are: A>C, C>A, A>T, T>A, C>G, G>C, G>T, T>G. Of the 8 possible transversions, half (4) are changes between complementary nucleotides (e.g., A>T) (Figure S15).


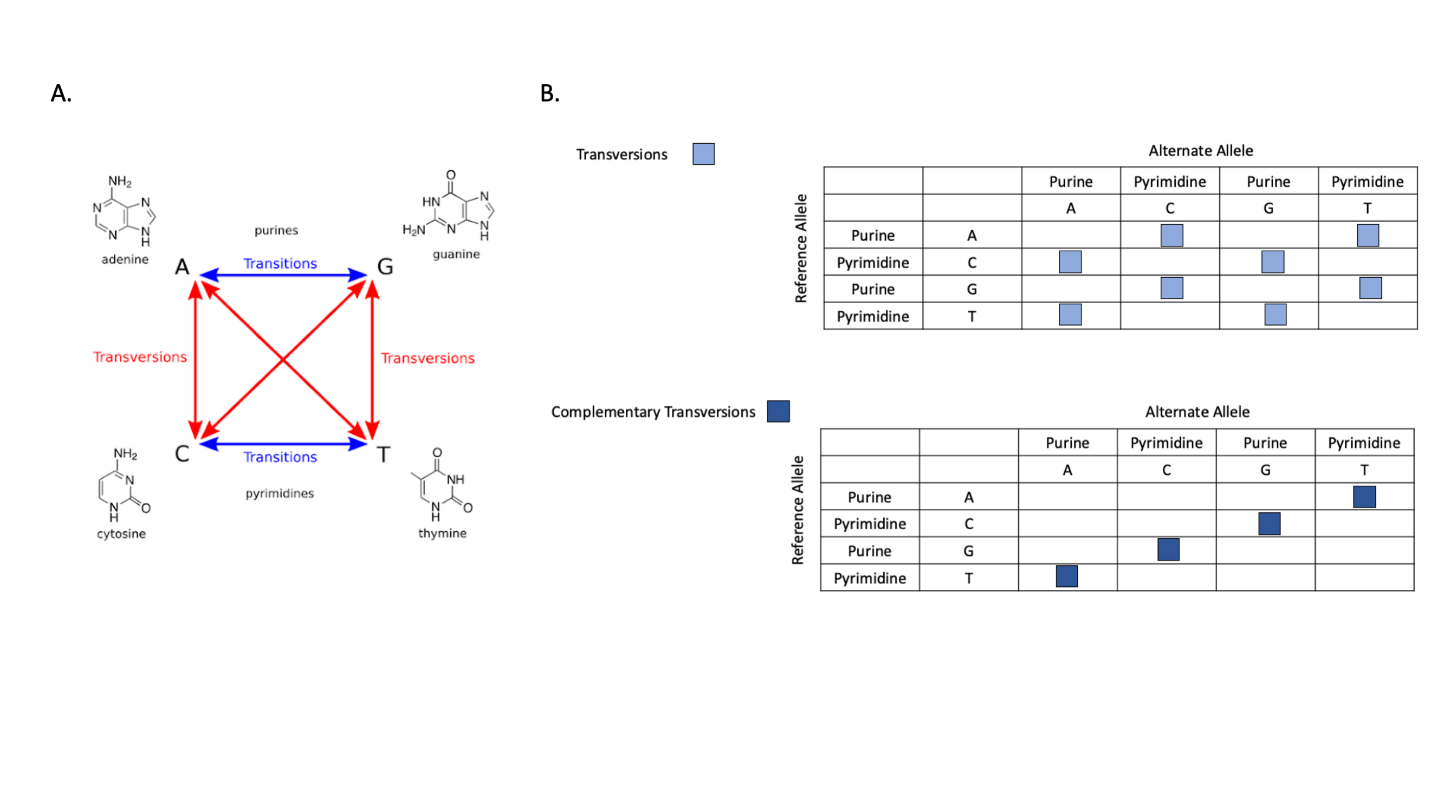


**Figure S15.** A schematic diagram showing the 12 possible transitions and transversions. (A) A schematic illustration of nucleotide substitutions leading to transitions (*blue arrows*) and transversions (*red arrows*). (B) Tabulated representation of all transversions (8) (*top table*) and complementary transversions (4) (*bottom table*)*.*  All transversions are shown as *light blue squares* in the top table, whereas complementary transversions are shown as *dark blue squares* in the bottom table.

Data submitted to NCBI

BioProject (PRJNA792997) was created at the National Center for Biotechnology Information (NCBI) for our study data. A total of 262 BioSample accession identifiers were created (Table S8) (Individual NA17281 did not consent to making genetic data publicly available and no data were submitted for this sample). The BAM files were submitted to the NCBI Sequence Read Archive (SRA) database via AWS S3 bucket and variation data for GSA and WGS were submitted to the NCBI dbSNP database (SFTP server).

Data were submitted to dbSNP database and will be publicly available when next dbSNP build (B156) is released. Our submitted data are accessible on NCBI FTP server: https://ftp.ncbi.nlm.nih.gov/snp/submission/SANFORD_IMAGENETICS/

Submitted variation data will receive a submitted SNP (ss) number and a reference SNP (rs) number. Variation that are assigned rs numbers are distributed as part of dbSNP once data are processed and released in the next build. Variation detected by GSA genotyping of 262 samples and by WGS genotyping of 260 samples were submitted to dbSNP (WGS variation data for HG00111 and HG00257 were not submitted to dbSNP database due to discordance between GSA and WGS data in our analyses).

SRA data are accessible on NCBI SRA database (https://www.ncbi.nlm.nih.gov/sra) via accession PRJNA792997 (261 samples’ submission id: SUB10951790; HG00111 submission id: SUB11086930 (accession id: SRR18019669)). All 262 samples’ BAM files’ sequence data were submitted to NCBI SRA database (Table S9).

**Table S8. List of 262 BioSample accessions submitted to NCBI.**

| **Accession** | **Sample Name** | **SPUID** | **Organism** | **Tax ID** | **Isolate** | **BioProject** |
| --- | --- | --- | --- | --- | --- | --- |
| SAMN24495081 | HG00096 | HG00096 | Homo sapiens | 9606 | HG00096 | PRJNA792997 |
| SAMN24495082 | HG00099 | HG00099 | Homo sapiens | 9606 | HG00099 | PRJNA792997 |
| SAMN24495083 | HG00101 | HG00101 | Homo sapiens | 9606 | HG00101 | PRJNA792997 |
| SAMN24495084 | HG00102 | HG00102 | Homo sapiens | 9606 | HG00102 | PRJNA792997 |
| SAMN24495085 | HG00103 | HG00103 | Homo sapiens | 9606 | HG00103 | PRJNA792997 |
| SAMN24495086 | HG00106 | HG00106 | Homo sapiens | 9606 | HG00106 | PRJNA792997 |
| SAMN24495087 | HG00108 | HG00108 | Homo sapiens | 9606 | HG00108 | PRJNA792997 |
| SAMN24495088 | HG00110 | HG00110 | Homo sapiens | 9606 | HG00110 | PRJNA792997 |
| SAMN24495089 | HG00111 | HG00111 | Homo sapiens | 9606 | HG00111 | PRJNA792997 |
| SAMN24495090 | HG00116 | HG00116 | Homo sapiens | 9606 | HG00116 | PRJNA792997 |
| SAMN24495091 | HG00118 | HG00118 | Homo sapiens | 9606 | HG00118 | PRJNA792997 |
| SAMN24495092 | HG00120 | HG00120 | Homo sapiens | 9606 | HG00120 | PRJNA792997 |
| SAMN24495093 | HG00122 | HG00122 | Homo sapiens | 9606 | HG00122 | PRJNA792997 |
| SAMN24495094 | HG00126 | HG00126 | Homo sapiens | 9606 | HG00126 | PRJNA792997 |
| SAMN24495095 | HG00133 | HG00133 | Homo sapiens | 9606 | HG00133 | PRJNA792997 |
| SAMN24495096 | HG00174 | HG00174 | Homo sapiens | 9606 | HG00174 | PRJNA792997 |
| SAMN24495097 | HG00185 | HG00185 | Homo sapiens | 9606 | HG00185 | PRJNA792997 |
| SAMN24495098 | HG00188 | HG00188 | Homo sapiens | 9606 | HG00188 | PRJNA792997 |
| SAMN24495099 | HG00253 | HG00253 | Homo sapiens | 9606 | HG00253 | PRJNA792997 |
| SAMN24495100 | HG00257 | HG00257 | Homo sapiens | 9606 | HG00257 | PRJNA792997 |
| SAMN24495101 | HG00263 | HG00263 | Homo sapiens | 9606 | HG00263 | PRJNA792997 |
| SAMN24495102 | HG00265 | HG00265 | Homo sapiens | 9606 | HG00265 | PRJNA792997 |
| SAMN24495103 | HG00266 | HG00266 | Homo sapiens | 9606 | HG00266 | PRJNA792997 |
| SAMN24495104 | HG00268 | HG00268 | Homo sapiens | 9606 | HG00268 | PRJNA792997 |
| SAMN24495105 | HG00276 | HG00276 | Homo sapiens | 9606 | HG00276 | PRJNA792997 |
| SAMN24495106 | HG00332 | HG00332 | Homo sapiens | 9606 | HG00332 | PRJNA792997 |
| SAMN24495107 | HG00345 | HG00345 | Homo sapiens | 9606 | HG00345 | PRJNA792997 |
| SAMN24495108 | HG00366 | HG00366 | Homo sapiens | 9606 | HG00366 | PRJNA792997 |
| SAMN24495109 | HG00403 | HG00403 | Homo sapiens | 9606 | HG00403 | PRJNA792997 |
| SAMN24495110 | HG00419 | HG00419 | Homo sapiens | 9606 | HG00419 | PRJNA792997 |
| SAMN24495111 | HG00452 | HG00452 | Homo sapiens | 9606 | HG00452 | PRJNA792997 |
| SAMN24495112 | HG00759 | HG00759 | Homo sapiens | 9606 | HG00759 | PRJNA792997 |
| SAMN24495113 | HG01051 | HG01051 | Homo sapiens | 9606 | HG01051 | PRJNA792997 |
| SAMN24495114 | HG01083 | HG01083 | Homo sapiens | 9606 | HG01083 | PRJNA792997 |
| SAMN24495115 | HG01089 | HG01089 | Homo sapiens | 9606 | HG01089 | PRJNA792997 |
| SAMN24495116 | HG01112 | HG01112 | Homo sapiens | 9606 | HG01112 | PRJNA792997 |
| SAMN24495117 | HG01170 | HG01170 | Homo sapiens | 9606 | HG01170 | PRJNA792997 |
| SAMN24495118 | HG01398 | HG01398 | Homo sapiens | 9606 | HG01398 | PRJNA792997 |
| SAMN24495119 | HG01441 | HG01441 | Homo sapiens | 9606 | HG01441 | PRJNA792997 |
| SAMN24495120 | HG01455 | HG01455 | Homo sapiens | 9606 | HG01455 | PRJNA792997 |
| SAMN24495121 | HG01500 | HG01500 | Homo sapiens | 9606 | HG01500 | PRJNA792997 |
| SAMN24495122 | HG01530 | HG01530 | Homo sapiens | 9606 | HG01530 | PRJNA792997 |
| SAMN24495123 | HG01565 | HG01565 | Homo sapiens | 9606 | HG01565 | PRJNA792997 |
| SAMN24495124 | HG01583 | HG01583 | Homo sapiens | 9606 | HG01583 | PRJNA792997 |
| SAMN24495125 | HG01595 | HG01595 | Homo sapiens | 9606 | HG01595 | PRJNA792997 |
| SAMN24495126 | HG01605 | HG01605 | Homo sapiens | 9606 | HG01605 | PRJNA792997 |
| SAMN24495127 | HG01620 | HG01620 | Homo sapiens | 9606 | HG01620 | PRJNA792997 |
| SAMN24495128 | HG01879 | HG01879 | Homo sapiens | 9606 | HG01879 | PRJNA792997 |
| SAMN24495129 | HG01941 | HG01941 | Homo sapiens | 9606 | HG01941 | PRJNA792997 |
| SAMN24495130 | HG01944 | HG01944 | Homo sapiens | 9606 | HG01944 | PRJNA792997 |
| SAMN24495131 | HG02012 | HG02012 | Homo sapiens | 9606 | HG02012 | PRJNA792997 |
| SAMN24495132 | HG02032 | HG02032 | Homo sapiens | 9606 | HG02032 | PRJNA792997 |
| SAMN24495133 | HG02052 | HG02052 | Homo sapiens | 9606 | HG02052 | PRJNA792997 |
| SAMN24495134 | HG02271 | HG02271 | Homo sapiens | 9606 | HG02271 | PRJNA792997 |
| SAMN24495135 | HG02304 | HG02304 | Homo sapiens | 9606 | HG02304 | PRJNA792997 |
| SAMN24495136 | HG02360 | HG02360 | Homo sapiens | 9606 | HG02360 | PRJNA792997 |
| SAMN24495137 | HG02568 | HG02568 | Homo sapiens | 9606 | HG02568 | PRJNA792997 |
| SAMN24495138 | HG02628 | HG02628 | Homo sapiens | 9606 | HG02628 | PRJNA792997 |
| SAMN24495139 | HG02651 | HG02651 | Homo sapiens | 9606 | HG02651 | PRJNA792997 |
| SAMN24495140 | HG02684 | HG02684 | Homo sapiens | 9606 | HG02684 | PRJNA792997 |
| SAMN24495141 | HG02922 | HG02922 | Homo sapiens | 9606 | HG02922 | PRJNA792997 |
| SAMN24495142 | HG03006 | HG03006 | Homo sapiens | 9606 | HG03006 | PRJNA792997 |
| SAMN24495143 | HG03052 | HG03052 | Homo sapiens | 9606 | HG03052 | PRJNA792997 |
| SAMN24495144 | HG03267 | HG03267 | Homo sapiens | 9606 | HG03267 | PRJNA792997 |
| SAMN24495145 | HG03279 | HG03279 | Homo sapiens | 9606 | HG03279 | PRJNA792997 |
| SAMN24495146 | HG03366 | HG03366 | Homo sapiens | 9606 | HG03366 | PRJNA792997 |
| SAMN24495147 | HG03369 | HG03369 | Homo sapiens | 9606 | HG03369 | PRJNA792997 |
| SAMN24495148 | HG03401 | HG03401 | Homo sapiens | 9606 | HG03401 | PRJNA792997 |
| SAMN24495149 | HG03464 | HG03464 | Homo sapiens | 9606 | HG03464 | PRJNA792997 |
| SAMN24495150 | HG03472 | HG03472 | Homo sapiens | 9606 | HG03472 | PRJNA792997 |
| SAMN24495151 | HG03514 | HG03514 | Homo sapiens | 9606 | HG03514 | PRJNA792997 |
| SAMN24495152 | HG03575 | HG03575 | Homo sapiens | 9606 | HG03575 | PRJNA792997 |
| SAMN24495153 | HG03642 | HG03642 | Homo sapiens | 9606 | HG03642 | PRJNA792997 |
| SAMN24495154 | HG03681 | HG03681 | Homo sapiens | 9606 | HG03681 | PRJNA792997 |
| SAMN24495155 | HG03685 | HG03685 | Homo sapiens | 9606 | HG03685 | PRJNA792997 |
| SAMN24495156 | HG03686 | HG03686 | Homo sapiens | 9606 | HG03686 | PRJNA792997 |
| SAMN24495157 | HG03687 | HG03687 | Homo sapiens | 9606 | HG03687 | PRJNA792997 |
| SAMN24495158 | HG03694 | HG03694 | Homo sapiens | 9606 | HG03694 | PRJNA792997 |
| SAMN24495159 | HG03705 | HG03705 | Homo sapiens | 9606 | HG03705 | PRJNA792997 |
| SAMN24495160 | HG03713 | HG03713 | Homo sapiens | 9606 | HG03713 | PRJNA792997 |
| SAMN24495161 | HG03742 | HG03742 | Homo sapiens | 9606 | HG03742 | PRJNA792997 |
| SAMN24495162 | HG03788 | HG03788 | Homo sapiens | 9606 | HG03788 | PRJNA792997 |
| SAMN24495163 | HG03790 | HG03790 | Homo sapiens | 9606 | HG03790 | PRJNA792997 |
| SAMN24495164 | HG03848 | HG03848 | Homo sapiens | 9606 | HG03848 | PRJNA792997 |
| SAMN24495165 | HG03854 | HG03854 | Homo sapiens | 9606 | HG03854 | PRJNA792997 |
| SAMN24495166 | HG03871 | HG03871 | Homo sapiens | 9606 | HG03871 | PRJNA792997 |
| SAMN24495167 | HG03885 | HG03885 | Homo sapiens | 9606 | HG03885 | PRJNA792997 |
| SAMN24495168 | HG03896 | HG03896 | Homo sapiens | 9606 | HG03896 | PRJNA792997 |
| SAMN24495169 | HG03967 | HG03967 | Homo sapiens | 9606 | HG03967 | PRJNA792997 |
| SAMN24495170 | HG03974 | HG03974 | Homo sapiens | 9606 | HG03974 | PRJNA792997 |
| SAMN24495171 | HG04060 | HG04060 | Homo sapiens | 9606 | HG04060 | PRJNA792997 |
| SAMN24495172 | HG04094 | HG04094 | Homo sapiens | 9606 | HG04094 | PRJNA792997 |
| SAMN24495173 | HG04131 | HG04131 | Homo sapiens | 9606 | HG04131 | PRJNA792997 |
| SAMN24495174 | HG04219 | HG04219 | Homo sapiens | 9606 | HG04219 | PRJNA792997 |
| SAMN24495175 | NA06989 | NA06989 | Homo sapiens | 9606 | NA06989 | PRJNA792997 |
| SAMN24495176 | NA07045 | NA07045 | Homo sapiens | 9606 | NA07045 | PRJNA792997 |
| SAMN24495177 | NA12156 | NA12156 | Homo sapiens | 9606 | NA12156 | PRJNA792997 |
| SAMN24495178 | NA12878 | NA12878 | Homo sapiens | 9606 | NA12878 | PRJNA792997 |
| SAMN24495180 | NA18486 | NA18486 | Homo sapiens | 9606 | NA18486 | PRJNA792997 |
| SAMN24495181 | NA18488 | NA18488 | Homo sapiens | 9606 | NA18488 | PRJNA792997 |
| SAMN24495182 | NA18508 | NA18508 | Homo sapiens | 9606 | NA18508 | PRJNA792997 |
| SAMN24495183 | NA18511 | NA18511 | Homo sapiens | 9606 | NA18511 | PRJNA792997 |
| SAMN24495184 | NA18525 | NA18525 | Homo sapiens | 9606 | NA18525 | PRJNA792997 |
| SAMN24495185 | NA18543 | NA18543 | Homo sapiens | 9606 | NA18543 | PRJNA792997 |
| SAMN24495186 | NA18544 | NA18544 | Homo sapiens | 9606 | NA18544 | PRJNA792997 |
| SAMN24495187 | NA18552 | NA18552 | Homo sapiens | 9606 | NA18552 | PRJNA792997 |
| SAMN24495188 | NA18559 | NA18559 | Homo sapiens | 9606 | NA18559 | PRJNA792997 |
| SAMN24495189 | NA18572 | NA18572 | Homo sapiens | 9606 | NA18572 | PRJNA792997 |
| SAMN24495190 | NA18606 | NA18606 | Homo sapiens | 9606 | NA18606 | PRJNA792997 |
| SAMN24495191 | NA18856 | NA18856 | Homo sapiens | 9606 | NA18856 | PRJNA792997 |
| SAMN24495192 | NA18861 | NA18861 | Homo sapiens | 9606 | NA18861 | PRJNA792997 |
| SAMN24495193 | NA18915 | NA18915 | Homo sapiens | 9606 | NA18915 | PRJNA792997 |
| SAMN24495194 | NA18916 | NA18916 | Homo sapiens | 9606 | NA18916 | PRJNA792997 |
| SAMN24495195 | NA18933 | NA18933 | Homo sapiens | 9606 | NA18933 | PRJNA792997 |
| SAMN24495196 | NA18939 | NA18939 | Homo sapiens | 9606 | NA18939 | PRJNA792997 |
| SAMN24495197 | NA18944 | NA18944 | Homo sapiens | 9606 | NA18944 | PRJNA792997 |
| SAMN24495198 | NA18948 | NA18948 | Homo sapiens | 9606 | NA18948 | PRJNA792997 |
| SAMN24495199 | NA18953 | NA18953 | Homo sapiens | 9606 | NA18953 | PRJNA792997 |
| SAMN24495200 | NA18959 | NA18959 | Homo sapiens | 9606 | NA18959 | PRJNA792997 |
| SAMN24495201 | NA18965 | NA18965 | Homo sapiens | 9606 | NA18965 | PRJNA792997 |
| SAMN24495202 | NA18966 | NA18966 | Homo sapiens | 9606 | NA18966 | PRJNA792997 |
| SAMN24495203 | NA18970 | NA18970 | Homo sapiens | 9606 | NA18970 | PRJNA792997 |
| SAMN24495204 | NA18971 | NA18971 | Homo sapiens | 9606 | NA18971 | PRJNA792997 |
| SAMN24495205 | NA18974 | NA18974 | Homo sapiens | 9606 | NA18974 | PRJNA792997 |
| SAMN24495206 | NA18983 | NA18983 | Homo sapiens | 9606 | NA18983 | PRJNA792997 |
| SAMN24495207 | NA18990 | NA18990 | Homo sapiens | 9606 | NA18990 | PRJNA792997 |
| SAMN24495208 | NA18994 | NA18994 | Homo sapiens | 9606 | NA18994 | PRJNA792997 |
| SAMN24495209 | NA19017 | NA19017 | Homo sapiens | 9606 | NA19017 | PRJNA792997 |
| SAMN24495210 | NA19019 | NA19019 | Homo sapiens | 9606 | NA19019 | PRJNA792997 |
| SAMN24495211 | NA19023 | NA19023 | Homo sapiens | 9606 | NA19023 | PRJNA792997 |
| SAMN24495212 | NA19024 | NA19024 | Homo sapiens | 9606 | NA19024 | PRJNA792997 |
| SAMN24495213 | NA19026 | NA19026 | Homo sapiens | 9606 | NA19026 | PRJNA792997 |
| SAMN24495214 | NA19031 | NA19031 | Homo sapiens | 9606 | NA19031 | PRJNA792997 |
| SAMN24495215 | NA19036 | NA19036 | Homo sapiens | 9606 | NA19036 | PRJNA792997 |
| SAMN24495216 | NA19037 | NA19037 | Homo sapiens | 9606 | NA19037 | PRJNA792997 |
| SAMN24495217 | NA19041 | NA19041 | Homo sapiens | 9606 | NA19041 | PRJNA792997 |
| SAMN24495218 | NA19042 | NA19042 | Homo sapiens | 9606 | NA19042 | PRJNA792997 |
| SAMN24495219 | NA19043 | NA19043 | Homo sapiens | 9606 | NA19043 | PRJNA792997 |
| SAMN24495220 | NA19056 | NA19056 | Homo sapiens | 9606 | NA19056 | PRJNA792997 |
| SAMN24495221 | NA19070 | NA19070 | Homo sapiens | 9606 | NA19070 | PRJNA792997 |
| SAMN24495222 | NA19072 | NA19072 | Homo sapiens | 9606 | NA19072 | PRJNA792997 |
| SAMN24495223 | NA19076 | NA19076 | Homo sapiens | 9606 | NA19076 | PRJNA792997 |
| SAMN24495224 | NA19102 | NA19102 | Homo sapiens | 9606 | NA19102 | PRJNA792997 |
| SAMN24495225 | NA19119 | NA19119 | Homo sapiens | 9606 | NA19119 | PRJNA792997 |
| SAMN24495226 | NA19130 | NA19130 | Homo sapiens | 9606 | NA19130 | PRJNA792997 |
| SAMN24495227 | NA19131 | NA19131 | Homo sapiens | 9606 | NA19131 | PRJNA792997 |
| SAMN24495228 | NA19147 | NA19147 | Homo sapiens | 9606 | NA19147 | PRJNA792997 |
| SAMN24495229 | NA19159 | NA19159 | Homo sapiens | 9606 | NA19159 | PRJNA792997 |
| SAMN24495230 | NA19160 | NA19160 | Homo sapiens | 9606 | NA19160 | PRJNA792997 |
| SAMN24495231 | NA19189 | NA19189 | Homo sapiens | 9606 | NA19189 | PRJNA792997 |
| SAMN24495232 | NA19209 | NA19209 | Homo sapiens | 9606 | NA19209 | PRJNA792997 |
| SAMN24495233 | NA19238 | NA19238 | Homo sapiens | 9606 | NA19238 | PRJNA792997 |
| SAMN24495234 | NA19239 | NA19239 | Homo sapiens | 9606 | NA19239 | PRJNA792997 |
| SAMN24495235 | NA19248 | NA19248 | Homo sapiens | 9606 | NA19248 | PRJNA792997 |
| SAMN24495236 | NA19308 | NA19308 | Homo sapiens | 9606 | NA19308 | PRJNA792997 |
| SAMN24495237 | NA19314 | NA19314 | Homo sapiens | 9606 | NA19314 | PRJNA792997 |
| SAMN24495238 | NA19319 | NA19319 | Homo sapiens | 9606 | NA19319 | PRJNA792997 |
| SAMN24495239 | NA19320 | NA19320 | Homo sapiens | 9606 | NA19320 | PRJNA792997 |
| SAMN24495240 | NA19321 | NA19321 | Homo sapiens | 9606 | NA19321 | PRJNA792997 |
| SAMN24495241 | NA19323 | NA19323 | Homo sapiens | 9606 | NA19323 | PRJNA792997 |
| SAMN24495242 | NA19324 | NA19324 | Homo sapiens | 9606 | NA19324 | PRJNA792997 |
| SAMN24495243 | NA19327 | NA19327 | Homo sapiens | 9606 | NA19327 | PRJNA792997 |
| SAMN24495244 | NA19347 | NA19347 | Homo sapiens | 9606 | NA19347 | PRJNA792997 |
| SAMN24495245 | NA19350 | NA19350 | Homo sapiens | 9606 | NA19350 | PRJNA792997 |
| SAMN24495246 | NA19355 | NA19355 | Homo sapiens | 9606 | NA19355 | PRJNA792997 |
| SAMN24495247 | NA19374 | NA19374 | Homo sapiens | 9606 | NA19374 | PRJNA792997 |
| SAMN24495248 | NA19376 | NA19376 | Homo sapiens | 9606 | NA19376 | PRJNA792997 |
| SAMN24495249 | NA19380 | NA19380 | Homo sapiens | 9606 | NA19380 | PRJNA792997 |
| SAMN24495250 | NA19390 | NA19390 | Homo sapiens | 9606 | NA19390 | PRJNA792997 |
| SAMN24495251 | NA19395 | NA19395 | Homo sapiens | 9606 | NA19395 | PRJNA792997 |
| SAMN24495252 | NA19399 | NA19399 | Homo sapiens | 9606 | NA19399 | PRJNA792997 |
| SAMN24495253 | NA19403 | NA19403 | Homo sapiens | 9606 | NA19403 | PRJNA792997 |
| SAMN24495254 | NA19429 | NA19429 | Homo sapiens | 9606 | NA19429 | PRJNA792997 |
| SAMN24495255 | NA19434 | NA19434 | Homo sapiens | 9606 | NA19434 | PRJNA792997 |
| SAMN24495256 | NA19435 | NA19435 | Homo sapiens | 9606 | NA19435 | PRJNA792997 |
| SAMN24495257 | NA19436 | NA19436 | Homo sapiens | 9606 | NA19436 | PRJNA792997 |
| SAMN24495258 | NA19437 | NA19437 | Homo sapiens | 9606 | NA19437 | PRJNA792997 |
| SAMN24495259 | NA19440 | NA19440 | Homo sapiens | 9606 | NA19440 | PRJNA792997 |
| SAMN24495260 | NA19443 | NA19443 | Homo sapiens | 9606 | NA19443 | PRJNA792997 |
| SAMN24495261 | NA19445 | NA19445 | Homo sapiens | 9606 | NA19445 | PRJNA792997 |
| SAMN24495262 | NA19452 | NA19452 | Homo sapiens | 9606 | NA19452 | PRJNA792997 |
| SAMN24495263 | NA19457 | NA19457 | Homo sapiens | 9606 | NA19457 | PRJNA792997 |
| SAMN24495264 | NA19466 | NA19466 | Homo sapiens | 9606 | NA19466 | PRJNA792997 |
| SAMN24495265 | NA19468 | NA19468 | Homo sapiens | 9606 | NA19468 | PRJNA792997 |
| SAMN24495266 | NA19471 | NA19471 | Homo sapiens | 9606 | NA19471 | PRJNA792997 |
| SAMN24495267 | NA19472 | NA19472 | Homo sapiens | 9606 | NA19472 | PRJNA792997 |
| SAMN24495268 | NA19473 | NA19473 | Homo sapiens | 9606 | NA19473 | PRJNA792997 |
| SAMN24495269 | NA19475 | NA19475 | Homo sapiens | 9606 | NA19475 | PRJNA792997 |
| SAMN24495270 | NA19625 | NA19625 | Homo sapiens | 9606 | NA19625 | PRJNA792997 |
| SAMN24495271 | NA19648 | NA19648 | Homo sapiens | 9606 | NA19648 | PRJNA792997 |
| SAMN24495272 | NA19658 | NA19658 | Homo sapiens | 9606 | NA19658 | PRJNA792997 |
| SAMN24495273 | NA19682 | NA19682 | Homo sapiens | 9606 | NA19682 | PRJNA792997 |
| SAMN24495274 | NA19700 | NA19700 | Homo sapiens | 9606 | NA19700 | PRJNA792997 |
| SAMN24495275 | NA19701 | NA19701 | Homo sapiens | 9606 | NA19701 | PRJNA792997 |
| SAMN24495276 | NA19704 | NA19704 | Homo sapiens | 9606 | NA19704 | PRJNA792997 |
| SAMN24495277 | NA19713 | NA19713 | Homo sapiens | 9606 | NA19713 | PRJNA792997 |
| SAMN24495278 | NA19764 | NA19764 | Homo sapiens | 9606 | NA19764 | PRJNA792997 |
| SAMN24495279 | NA19780 | NA19780 | Homo sapiens | 9606 | NA19780 | PRJNA792997 |
| SAMN24495280 | NA19789 | NA19789 | Homo sapiens | 9606 | NA19789 | PRJNA792997 |
| SAMN24495281 | NA19795 | NA19795 | Homo sapiens | 9606 | NA19795 | PRJNA792997 |
| SAMN24495282 | NA19834 | NA19834 | Homo sapiens | 9606 | NA19834 | PRJNA792997 |
| SAMN24495283 | NA19835 | NA19835 | Homo sapiens | 9606 | NA19835 | PRJNA792997 |
| SAMN24495284 | NA19909 | NA19909 | Homo sapiens | 9606 | NA19909 | PRJNA792997 |
| SAMN24495285 | NA19921 | NA19921 | Homo sapiens | 9606 | NA19921 | PRJNA792997 |
| SAMN24495286 | NA19984 | NA19984 | Homo sapiens | 9606 | NA19984 | PRJNA792997 |
| SAMN24495287 | NA20126 | NA20126 | Homo sapiens | 9606 | NA20126 | PRJNA792997 |
| SAMN24495288 | NA20274 | NA20274 | Homo sapiens | 9606 | NA20274 | PRJNA792997 |
| SAMN24495289 | NA20276 | NA20276 | Homo sapiens | 9606 | NA20276 | PRJNA792997 |
| SAMN24495290 | NA20278 | NA20278 | Homo sapiens | 9606 | NA20278 | PRJNA792997 |
| SAMN24495291 | NA20287 | NA20287 | Homo sapiens | 9606 | NA20287 | PRJNA792997 |
| SAMN24495292 | NA20289 | NA20289 | Homo sapiens | 9606 | NA20289 | PRJNA792997 |
| SAMN24495293 | NA20291 | NA20291 | Homo sapiens | 9606 | NA20291 | PRJNA792997 |
| SAMN24495294 | NA20294 | NA20294 | Homo sapiens | 9606 | NA20294 | PRJNA792997 |
| SAMN24495295 | NA20296 | NA20296 | Homo sapiens | 9606 | NA20296 | PRJNA792997 |
| SAMN24495296 | NA20299 | NA20299 | Homo sapiens | 9606 | NA20299 | PRJNA792997 |
| SAMN24495297 | NA20320 | NA20320 | Homo sapiens | 9606 | NA20320 | PRJNA792997 |
| SAMN24495298 | NA20321 | NA20321 | Homo sapiens | 9606 | NA20321 | PRJNA792997 |
| SAMN24495299 | NA20332 | NA20332 | Homo sapiens | 9606 | NA20332 | PRJNA792997 |
| SAMN24495300 | NA20339 | NA20339 | Homo sapiens | 9606 | NA20339 | PRJNA792997 |
| SAMN24495301 | NA20342 | NA20342 | Homo sapiens | 9606 | NA20342 | PRJNA792997 |
| SAMN24495302 | NA20346 | NA20346 | Homo sapiens | 9606 | NA20346 | PRJNA792997 |
| SAMN24495303 | NA20348 | NA20348 | Homo sapiens | 9606 | NA20348 | PRJNA792997 |
| SAMN24495304 | NA20351 | NA20351 | Homo sapiens | 9606 | NA20351 | PRJNA792997 |
| SAMN24495305 | NA20355 | NA20355 | Homo sapiens | 9606 | NA20355 | PRJNA792997 |
| SAMN24495306 | NA20356 | NA20356 | Homo sapiens | 9606 | NA20356 | PRJNA792997 |
| SAMN24495307 | NA20412 | NA20412 | Homo sapiens | 9606 | NA20412 | PRJNA792997 |
| SAMN24495308 | NA20502 | NA20502 | Homo sapiens | 9606 | NA20502 | PRJNA792997 |
| SAMN24495309 | NA20509 | NA20509 | Homo sapiens | 9606 | NA20509 | PRJNA792997 |
| SAMN24495310 | NA20510 | NA20510 | Homo sapiens | 9606 | NA20510 | PRJNA792997 |
| SAMN24495311 | NA20511 | NA20511 | Homo sapiens | 9606 | NA20511 | PRJNA792997 |
| SAMN24495312 | NA20513 | NA20513 | Homo sapiens | 9606 | NA20513 | PRJNA792997 |
| SAMN24495313 | NA20515 | NA20515 | Homo sapiens | 9606 | NA20515 | PRJNA792997 |
| SAMN24495314 | NA20516 | NA20516 | Homo sapiens | 9606 | NA20516 | PRJNA792997 |
| SAMN24495315 | NA20519 | NA20519 | Homo sapiens | 9606 | NA20519 | PRJNA792997 |
| SAMN24495316 | NA20520 | NA20520 | Homo sapiens | 9606 | NA20520 | PRJNA792997 |
| SAMN24495317 | NA20524 | NA20524 | Homo sapiens | 9606 | NA20524 | PRJNA792997 |
| SAMN24495318 | NA20525 | NA20525 | Homo sapiens | 9606 | NA20525 | PRJNA792997 |
| SAMN24495319 | NA20532 | NA20532 | Homo sapiens | 9606 | NA20532 | PRJNA792997 |
| SAMN24495320 | NA20763 | NA20763 | Homo sapiens | 9606 | NA20763 | PRJNA792997 |
| SAMN24495321 | NA20767 | NA20767 | Homo sapiens | 9606 | NA20767 | PRJNA792997 |
| SAMN24495322 | NA20812 | NA20812 | Homo sapiens | 9606 | NA20812 | PRJNA792997 |
| SAMN24495323 | NA20814 | NA20814 | Homo sapiens | 9606 | NA20814 | PRJNA792997 |
| SAMN24495324 | NA20822 | NA20822 | Homo sapiens | 9606 | NA20822 | PRJNA792997 |
| SAMN24495325 | NA20845 | NA20845 | Homo sapiens | 9606 | NA20845 | PRJNA792997 |
| SAMN24495326 | NA20846 | NA20846 | Homo sapiens | 9606 | NA20846 | PRJNA792997 |
| SAMN24495327 | NA20863 | NA20863 | Homo sapiens | 9606 | NA20863 | PRJNA792997 |
| SAMN24495328 | NA20864 | NA20864 | Homo sapiens | 9606 | NA20864 | PRJNA792997 |
| SAMN24495329 | NA20867 | NA20867 | Homo sapiens | 9606 | NA20867 | PRJNA792997 |
| SAMN24495330 | NA20887 | NA20887 | Homo sapiens | 9606 | NA20887 | PRJNA792997 |
| SAMN24495331 | NA20889 | NA20889 | Homo sapiens | 9606 | NA20889 | PRJNA792997 |
| SAMN24495332 | NA20890 | NA20890 | Homo sapiens | 9606 | NA20890 | PRJNA792997 |
| SAMN24495333 | NA20901 | NA20901 | Homo sapiens | 9606 | NA20901 | PRJNA792997 |
| SAMN24495334 | NA20904 | NA20904 | Homo sapiens | 9606 | NA20904 | PRJNA792997 |
| SAMN24495335 | NA21090 | NA21090 | Homo sapiens | 9606 | NA21090 | PRJNA792997 |
| SAMN24495336 | NA21093 | NA21093 | Homo sapiens | 9606 | NA21093 | PRJNA792997 |
| SAMN24495337 | NA21105 | NA21105 | Homo sapiens | 9606 | NA21105 | PRJNA792997 |
| SAMN24495338 | NA21118 | NA21118 | Homo sapiens | 9606 | NA21118 | PRJNA792997 |
| SAMN24495339 | NA21128 | NA21128 | Homo sapiens | 9606 | NA21128 | PRJNA792997 |
| SAMN24495340 | NA21135 | NA21135 | Homo sapiens | 9606 | NA21135 | PRJNA792997 |
| SAMN24495341 | NA24143 | NA24143 | Homo sapiens | 9606 | NA24143 | PRJNA792997 |
| SAMN24495342 | NA24149 | NA24149 | Homo sapiens | 9606 | NA24149 | PRJNA792997 |
| SAMN24495343 | NA24385 | NA24385 | Homo sapiens | 9606 | NA24385 | PRJNA792997 |

**Table S9. SRA accession numbers of submitted WGS BAM file data for 262 samples**

| Accession | Study | Bioproject_accession | Biosample_accession | Library_ID | Filename |
| --- | --- | --- | --- | --- | --- |
| SRR17635100 | SRP355248 | PRJNA792997 | SAMN24495081 | FD01114910 | HG00096.bam |
| SRR17635099 | SRP355248 | PRJNA792997 | SAMN24495082 | FD01114909 | HG00099.bam |
| SRR17634890 | SRP355248 | PRJNA792997 | SAMN24495083 | FD01114908 | HG00101.bam |
| SRR17634983 | SRP355248 | PRJNA792997 | SAMN24495084 | FD01114907 | HG00102.bam |
| SRR17634973 | SRP355248 | PRJNA792997 | SAMN24495085 | FD01114906 | HG00103.bam |
| SRR17634930 | SRP355248 | PRJNA792997 | SAMN24495086 | FD01114905 | HG00106.bam |
| SRR17634918 | SRP355248 | PRJNA792997 | SAMN24495087 | FD01114822 | HG00108.bam |
| SRR17634870 | SRP355248 | PRJNA792997 | SAMN24495088 | FD01114834 | HG00110.bam |
| SRR17634859 | SRP355248 | PRJNA792997 | SAMN24495090 | FD01114901 | HG00116.bam |
| SRR17634848 | SRP355248 | PRJNA792997 | SAMN24495091 | FD01114900 | HG00118.bam |
| SRR17635098 | SRP355248 | PRJNA792997 | SAMN24495092 | FD01114899 | HG00120.bam |
| SRR17635087 | SRP355248 | PRJNA792997 | SAMN24495093 | FD01114898 | HG00122.bam |
| SRR17635076 | SRP355248 | PRJNA792997 | SAMN24495094 | FD01114820 | HG00126.bam |
| SRR17635033 | SRP355248 | PRJNA792997 | SAMN24495095 | FD01114819 | HG00133.bam |
| SRR17635011 | SRP355248 | PRJNA792997 | SAMN24495096 | FD01114818 | HG00174.bam |
| SRR17634965 | SRP355248 | PRJNA792997 | SAMN24495097 | FD01114894 | HG00185.bam |
| SRR17635008 | SRP355248 | PRJNA792997 | SAMN24495098 | FD01114893 | HG00188.bam |
| SRR17634902 | SRP355248 | PRJNA792997 | SAMN24495099 | FD01114892 | HG00253.bam |
| SRR17634950 | SRP355248 | PRJNA792997 | SAMN24495100 | FD01114891 | HG00257.bam |
| SRR17634880 | SRP355248 | PRJNA792997 | SAMN24495101 | FD01114890 | HG00263.bam |
| SRR17634889 | SRP355248 | PRJNA792997 | SAMN24495102 | FD01114830 | HG00265.bam |
| SRR17635052 | SRP355248 | PRJNA792997 | SAMN24495103 | FD01114829 | HG00266.bam |
| SRR17635063 | SRP355248 | PRJNA792997 | SAMN24495104 | FD02929129 | HG00268.bam |
| SRR17634999 | SRP355248 | PRJNA792997 | SAMN24495105 | FD01114843 | HG00276.bam |
| SRR17635042 | SRP355248 | PRJNA792997 | SAMN24495106 | FD01114886 | HG00332.bam |
| SRR17634978 | SRP355248 | PRJNA792997 | SAMN24495107 | FD01114885 | HG00345.bam |
| SRR17634988 | SRP355248 | PRJNA792997 | SAMN24495108 | FD01114884 | HG00366.bam |
| SRR17634986 | SRP355248 | PRJNA792997 | SAMN24495109 | FD01114883 | HG00403.bam |
| SRR17634985 | SRP355248 | PRJNA792997 | SAMN24495110 | FD01114882 | HG00419.bam |
| SRR17634984 | SRP355248 | PRJNA792997 | SAMN24495111 | FD01114828 | HG00452.bam |
| SRR17634982 | SRP355248 | PRJNA792997 | SAMN24495112 | FD02929306 | HG00759.bam |
| SRR17634981 | SRP355248 | PRJNA792997 | SAMN24495113 | FD01114827 | HG01051.bam |
| SRR17634980 | SRP355248 | PRJNA792997 | SAMN24495114 | FD01115359 | HG01083.bam |
| SRR17634979 | SRP355248 | PRJNA792997 | SAMN24495115 | FD01114826 | HG01089.bam |
| SRR17634925 | SRP355248 | PRJNA792997 | SAMN24495116 | FD02929205 | HG01112.bam |
| SRR17634924 | SRP355248 | PRJNA792997 | SAMN24495117 | FD01114878 | HG01170.bam |
| SRR17634923 | SRP355248 | PRJNA792997 | SAMN24495118 | FD01114877 | HG01398.bam |
| SRR17634922 | SRP355248 | PRJNA792997 | SAMN24495119 | FD02929315 | HG01441.bam |
| SRR17634975 | SRP355248 | PRJNA792997 | SAMN24495120 | FD01115367 | HG01455.bam |
| SRR17634974 | SRP355248 | PRJNA792997 | SAMN24495121 | FD02929254 | HG01500.bam |
| SRR17634940 | SRP355248 | PRJNA792997 | SAMN24495122 | FD01115312 | HG01530.bam |
| SRR17634939 | SRP355248 | PRJNA792997 | SAMN24495123 | FD02929260 | HG01565.bam |
| SRR17634938 | SRP355248 | PRJNA792997 | SAMN24495124 | FD02929265 | HG01583.bam |
| SRR17634937 | SRP355248 | PRJNA792997 | SAMN24495125 | FD02929267 | HG01595.bam |
| SRR17634936 | SRP355248 | PRJNA792997 | SAMN24495126 | FD01115375 | HG01605.bam |
| SRR17634935 | SRP355248 | PRJNA792997 | SAMN24495127 | FD01114876 | HG01620.bam |
| SRR17634934 | SRP355248 | PRJNA792997 | SAMN24495128 | FD02929294 | HG01879.bam |
| SRR17634933 | SRP355248 | PRJNA792997 | SAMN24495129 | FD02929266 | HG01941.bam |
| SRR17634932 | SRP355248 | PRJNA792997 | SAMN24495130 | FD01115328 | HG01944.bam |
| SRR17634931 | SRP355248 | PRJNA792997 | SAMN24495131 | FD01114875 | HG02012.bam |
| SRR17634929 | SRP355248 | PRJNA792997 | SAMN24495132 | FD02929273 | HG02032.bam |
| SRR17634928 | SRP355248 | PRJNA792997 | SAMN24495133 | FD01114874 | HG02052.bam |
| SRR17634927 | SRP355248 | PRJNA792997 | SAMN24495134 | FD02929274 | HG02271.bam |
| SRR17634926 | SRP355248 | PRJNA792997 | SAMN24495135 | FD02929304 | HG02304.bam |
| SRR17634865 | SRP355248 | PRJNA792997 | SAMN24495136 | FD02929299 | HG02360.bam |
| SRR17634864 | SRP355248 | PRJNA792997 | SAMN24495137 | FD02929277 | HG02568.bam |
| SRR17634863 | SRP355248 | PRJNA792997 | SAMN24495138 | FD01114838 | HG02628.bam |
| SRR17634921 | SRP355248 | PRJNA792997 | SAMN24495139 | FD02929298 | HG02651.bam |
| SRR17634920 | SRP355248 | PRJNA792997 | SAMN24495140 | FD01114835 | HG02684.bam |
| SRR17634919 | SRP355248 | PRJNA792997 | SAMN24495141 | FD02929282 | HG02922.bam |
| SRR17634917 | SRP355248 | PRJNA792997 | SAMN24495142 | FD02929255 | HG03006.bam |
| SRR17634916 | SRP355248 | PRJNA792997 | SAMN24495143 | FD02929259 | HG03052.bam |
| SRR17634915 | SRP355248 | PRJNA792997 | SAMN24495144 | FD01114842 | HG03267.bam |
| SRR17634914 | SRP355248 | PRJNA792997 | SAMN24495145 | FD02929207 | HG03279.bam |
| SRR17634913 | SRP355248 | PRJNA792997 | SAMN24495146 | FD02929209 | HG03366.bam |
| SRR17634912 | SRP355248 | PRJNA792997 | SAMN24495147 | FD02929204 | HG03369.bam |
| SRR17634911 | SRP355248 | PRJNA792997 | SAMN24495148 | FD02929202 | HG03401.bam |
| SRR17634910 | SRP355248 | PRJNA792997 | SAMN24495149 | FD02929293 | HG03464.bam |
| SRR17634909 | SRP355248 | PRJNA792997 | SAMN24495150 | FD02929301 | HG03472.bam |
| SRR17634871 | SRP355248 | PRJNA792997 | SAMN24495151 | FD02929271 | HG03514.bam |
| SRR17634869 | SRP355248 | PRJNA792997 | SAMN24495152 | FD02929151 | HG03575.bam |
| SRR17634868 | SRP355248 | PRJNA792997 | SAMN24495153 | FD02929237 | HG03642.bam |
| SRR17634867 | SRP355248 | PRJNA792997 | SAMN24495154 | FD02929251 | HG03681.bam |
| SRR17634866 | SRP355248 | PRJNA792997 | SAMN24495155 | FD02929256 | HG03685.bam |
| SRR17634843 | SRP355248 | PRJNA792997 | SAMN24495156 | FD02929244 | HG03686.bam |
| SRR17634842 | SRP355248 | PRJNA792997 | SAMN24495157 | FD02929250 | HG03687.bam |
| SRR17634841 | SRP355248 | PRJNA792997 | SAMN24495158 | FD02929300 | HG03694.bam |
| SRR17634862 | SRP355248 | PRJNA792997 | SAMN24495159 | FD02929144 | HG03705.bam |
| SRR17634861 | SRP355248 | PRJNA792997 | SAMN24495160 | FD02929228 | HG03713.bam |
| SRR17634860 | SRP355248 | PRJNA792997 | SAMN24495161 | FD02929303 | HG03742.bam |
| SRR17634858 | SRP355248 | PRJNA792997 | SAMN24495162 | FD02929229 | HG03788.bam |
| SRR17634857 | SRP355248 | PRJNA792997 | SAMN24495163 | FD02929230 | HG03790.bam |
| SRR17634856 | SRP355248 | PRJNA792997 | SAMN24495164 | FD02929257 | HG03848.bam |
| SRR17634855 | SRP355248 | PRJNA792997 | SAMN24495165 | FD02929239 | HG03854.bam |
| SRR17634854 | SRP355248 | PRJNA792997 | SAMN24495166 | FD02929242 | HG03871.bam |
| SRR17634853 | SRP355248 | PRJNA792997 | SAMN24495167 | FD02929262 | HG03885.bam |
| SRR17634852 | SRP355248 | PRJNA792997 | SAMN24495168 | FD02929245 | HG03896.bam |
| SRR17634851 | SRP355248 | PRJNA792997 | SAMN24495169 | FD02929232 | HG03967.bam |
| SRR17634850 | SRP355248 | PRJNA792997 | SAMN24495170 | FD02929310 | HG03974.bam |
| SRR17634849 | SRP355248 | PRJNA792997 | SAMN24495171 | FD01114870 | HG04060.bam |
| SRR17634847 | SRP355248 | PRJNA792997 | SAMN24495172 | FD02929243 | HG04094.bam |
| SRR17634846 | SRP355248 | PRJNA792997 | SAMN24495173 | FD02929241 | HG04131.bam |
| SRR17634845 | SRP355248 | PRJNA792997 | SAMN24495174 | FD02929240 | HG04219.bam |
| SRR17634844 | SRP355248 | PRJNA792997 | SAMN24495175 | FD01114869 | NA06989.bam |
| SRR17634906 | SRP355248 | PRJNA792997 | SAMN24495176 | FD01114868 | NA07045.bam |
| SRR17634905 | SRP355248 | PRJNA792997 | SAMN24495177 | FD01114867 | NA12156.bam |
| SRR17634904 | SRP355248 | PRJNA792997 | SAMN24495178 | FD01114866 | NA12878.bam |
| SRR17634840 | SRP355248 | PRJNA792997 | SAMN24495180 | FD01114837 | NA18486.bam |
| SRR17634908 | SRP355248 | PRJNA792997 | SAMN24495181 | FD02929297 | NA18488.bam |
| SRR17634907 | SRP355248 | PRJNA792997 | SAMN24495182 | FD02929279 | NA18508.bam |
| SRR17635097 | SRP355248 | PRJNA792997 | SAMN24495183 | FD01115383 | NA18511.bam |
| SRR17635096 | SRP355248 | PRJNA792997 | SAMN24495184 | FD02929309 | NA18525.bam |
| SRR17635095 | SRP355248 | PRJNA792997 | SAMN24495185 | FD02929317 | NA18543.bam |
| SRR17635094 | SRP355248 | PRJNA792997 | SAMN24495186 | FD02929287 | NA18544.bam |
| SRR17635093 | SRP355248 | PRJNA792997 | SAMN24495187 | FD01114836 | NA18552.bam |
| SRR17635092 | SRP355248 | PRJNA792997 | SAMN24495188 | FD02929312 | NA18559.bam |
| SRR17635091 | SRP355248 | PRJNA792997 | SAMN24495189 | FD02929136 | NA18572.bam |
| SRR17635090 | SRP355248 | PRJNA792997 | SAMN24495190 | FD02929311 | NA18606.bam |
| SRR17635089 | SRP355248 | PRJNA792997 | SAMN24495191 | FD01114821 | NA18856.bam |
| SRR17635088 | SRP355248 | PRJNA792997 | SAMN24495192 | FD02929160 | NA18861.bam |
| SRR17635086 | SRP355248 | PRJNA792997 | SAMN24495193 | FD02929218 | NA18915.bam |
| SRR17635085 | SRP355248 | PRJNA792997 | SAMN24495194 | FD02929236 | NA18916.bam |
| SRR17635084 | SRP355248 | PRJNA792997 | SAMN24495195 | FD01114862 | NA18933.bam |
| SRR17635083 | SRP355248 | PRJNA792997 | SAMN24495196 | FD02929270 | NA18939.bam |
| SRR17635082 | SRP355248 | PRJNA792997 | SAMN24495197 | FD02929263 | NA18944.bam |
| SRR17635081 | SRP355248 | PRJNA792997 | SAMN24495198 | FD02929258 | NA18948.bam |
| SRR17635080 | SRP355248 | PRJNA792997 | SAMN24495199 | FD02929288 | NA18953.bam |
| SRR17635079 | SRP355248 | PRJNA792997 | SAMN24495200 | FD02929220 | NA18959.bam |
| SRR17635078 | SRP355248 | PRJNA792997 | SAMN24495201 | FD02929261 | NA18965.bam |
| SRR17635077 | SRP355248 | PRJNA792997 | SAMN24495202 | FD02929231 | NA18966.bam |
| SRR17635075 | SRP355248 | PRJNA792997 | SAMN24495203 | FD02929247 | NA18970.bam |
| SRR17635074 | SRP355248 | PRJNA792997 | SAMN24495204 | FD01114861 | NA18971.bam |
| SRR17635073 | SRP355248 | PRJNA792997 | SAMN24495205 | FD02929210 | NA18974.bam |
| SRR17635072 | SRP355248 | PRJNA792997 | SAMN24495206 | FD02929219 | NA18983.bam |
| SRR17635071 | SRP355248 | PRJNA792997 | SAMN24495207 | FD02929208 | NA18990.bam |
| SRR17635070 | SRP355248 | PRJNA792997 | SAMN24495208 | FD02929249 | NA18994.bam |
| SRR17635069 | SRP355248 | PRJNA792997 | SAMN24495209 | FD02929217 | NA19017.bam |
| SRR17635036 | SRP355248 | PRJNA792997 | SAMN24495210 | FD02929212 | NA19019.bam |
| SRR17635035 | SRP355248 | PRJNA792997 | SAMN24495211 | FD02929139 | NA19023.bam |
| SRR17635034 | SRP355248 | PRJNA792997 | SAMN24495212 | FD02929269 | NA19024.bam |
| SRR17635032 | SRP355248 | PRJNA792997 | SAMN24495213 | FD02929215 | NA19026.bam |
| SRR17635031 | SRP355248 | PRJNA792997 | SAMN24495214 | FD02929193 | NA19031.bam |
| SRR17635030 | SRP355248 | PRJNA792997 | SAMN24495215 | FD02929191 | NA19036.bam |
| SRR17635029 | SRP355248 | PRJNA792997 | SAMN24495216 | FD02929176 | NA19037.bam |
| SRR17635028 | SRP355248 | PRJNA792997 | SAMN24495217 | FD02929201 | NA19041.bam |
| SRR17635027 | SRP355248 | PRJNA792997 | SAMN24495218 | FD02929188 | NA19042.bam |
| SRR17635026 | SRP355248 | PRJNA792997 | SAMN24495219 | FD02929199 | NA19043.bam |
| SRR17635025 | SRP355248 | PRJNA792997 | SAMN24495220 | FD02929216 | NA19056.bam |
| SRR17635024 | SRP355248 | PRJNA792997 | SAMN24495221 | FD02929189 | NA19070.bam |
| SRR17635023 | SRP355248 | PRJNA792997 | SAMN24495222 | FD02929221 | NA19072.bam |
| SRR17635012 | SRP355248 | PRJNA792997 | SAMN24495223 | FD02929238 | NA19076.bam |
| SRR17635013 | SRP355248 | PRJNA792997 | SAMN24495224 | FD02929272 | NA19102.bam |
| SRR17635014 | SRP355248 | PRJNA792997 | SAMN24495225 | FD02929246 | NA19119.bam |
| SRR17635015 | SRP355248 | PRJNA792997 | SAMN24495226 | FD02929291 | NA19130.bam |
| SRR17635016 | SRP355248 | PRJNA792997 | SAMN24495227 | FD02929224 | NA19131.bam |
| SRR17635017 | SRP355248 | PRJNA792997 | SAMN24495228 | FD02929164 | NA19147.bam |
| SRR17635018 | SRP355248 | PRJNA792997 | SAMN24495229 | FD02929134 | NA19159.bam |
| SRR17635019 | SRP355248 | PRJNA792997 | SAMN24495230 | FD02929227 | NA19160.bam |
| SRR17635021 | SRP355248 | PRJNA792997 | SAMN24495231 | FD02929313 | NA19189.bam |
| SRR17635022 | SRP355248 | PRJNA792997 | SAMN24495232 | FD02929234 | NA19209.bam |
| SRR17634966 | SRP355248 | PRJNA792997 | SAMN24495233 | FD01114854 | NA19238.bam |
| SRR17634967 | SRP355248 | PRJNA792997 | SAMN24495234 | FD01114853 | NA19239.bam |
| SRR17634968 | SRP355248 | PRJNA792997 | SAMN24495235 | FD01114860 | NA19248.bam |
| SRR17634969 | SRP355248 | PRJNA792997 | SAMN24495236 | FD02929150 | NA19308.bam |
| SRR17634970 | SRP355248 | PRJNA792997 | SAMN24495237 | FD02929132 | NA19314.bam |
| SRR17634971 | SRP355248 | PRJNA792997 | SAMN24495238 | FD02929135 | NA19319.bam |
| SRR17634972 | SRP355248 | PRJNA792997 | SAMN24495239 | FD02929275 | NA19320.bam |
| SRR17635005 | SRP355248 | PRJNA792997 | SAMN24495240 | FD02929141 | NA19321.bam |
| SRR17635006 | SRP355248 | PRJNA792997 | SAMN24495241 | FD02929130 | NA19323.bam |
| SRR17635007 | SRP355248 | PRJNA792997 | SAMN24495242 | FD02929140 | NA19324.bam |
| SRR17635009 | SRP355248 | PRJNA792997 | SAMN24495243 | FD02929194 | NA19327.bam |
| SRR17635010 | SRP355248 | PRJNA792997 | SAMN24495244 | FD02929131 | NA19347.bam |
| SRR17634964 | SRP355248 | PRJNA792997 | SAMN24495245 | FD02929190 | NA19350.bam |
| SRR17635020 | SRP355248 | PRJNA792997 | SAMN24495246 | FD02929175 | NA19355.bam |
| SRR17634963 | SRP355248 | PRJNA792997 | SAMN24495247 | FD02929187 | NA19374.bam |
| SRR17634962 | SRP355248 | PRJNA792997 | SAMN24495248 | FD02929172 | NA19376.bam |
| SRR17634961 | SRP355248 | PRJNA792997 | SAMN24495249 | FD02929127 | NA19380.bam |
| SRR17634960 | SRP355248 | PRJNA792997 | SAMN24495250 | FD02929137 | NA19390.bam |
| SRR17634959 | SRP355248 | PRJNA792997 | SAMN24495251 | FD02929196 | NA19395.bam |
| SRR17634958 | SRP355248 | PRJNA792997 | SAMN24495252 | FD02929292 | NA19399.bam |
| SRR17634901 | SRP355248 | PRJNA792997 | SAMN24495253 | FD02929195 | NA19403.bam |
| SRR17634900 | SRP355248 | PRJNA792997 | SAMN24495254 | FD02929278 | NA19429.bam |
| SRR17634899 | SRP355248 | PRJNA792997 | SAMN24495255 | FD02929142 | NA19434.bam |
| SRR17634957 | SRP355248 | PRJNA792997 | SAMN24495256 | FD02929295 | NA19435.bam |
| SRR17634956 | SRP355248 | PRJNA792997 | SAMN24495257 | FD02929149 | NA19436.bam |
| SRR17634955 | SRP355248 | PRJNA792997 | SAMN24495258 | FD01115352 | NA19437.bam |
| SRR17634954 | SRP355248 | PRJNA792997 | SAMN24495259 | FD02929222 | NA19440.bam |
| SRR17634953 | SRP355248 | PRJNA792997 | SAMN24495260 | FD02929289 | NA19443.bam |
| SRR17634952 | SRP355248 | PRJNA792997 | SAMN24495261 | FD02929197 | NA19445.bam |
| SRR17634951 | SRP355248 | PRJNA792997 | SAMN24495262 | FD02929198 | NA19452.bam |
| SRR17634949 | SRP355248 | PRJNA792997 | SAMN24495263 | FD02929290 | NA19457.bam |
| SRR17634948 | SRP355248 | PRJNA792997 | SAMN24495264 | FD02929186 | NA19466.bam |
| SRR17634947 | SRP355248 | PRJNA792997 | SAMN24495265 | FD02929169 | NA19468.bam |
| SRR17634946 | SRP355248 | PRJNA792997 | SAMN24495266 | FD02929285 | NA19471.bam |
| SRR17634945 | SRP355248 | PRJNA792997 | SAMN24495267 | FD02929316 | NA19472.bam |
| SRR17634944 | SRP355248 | PRJNA792997 | SAMN24495268 | FD02929280 | NA19473.bam |
| SRR17634943 | SRP355248 | PRJNA792997 | SAMN24495269 | FD02929305 | NA19475.bam |
| SRR17634942 | SRP355248 | PRJNA792997 | SAMN24495270 | FD02929179 | NA19625.bam |
| SRR17634941 | SRP355248 | PRJNA792997 | SAMN24495271 | FD02929264 | NA19648.bam |
| SRR17634903 | SRP355248 | PRJNA792997 | SAMN24495272 | FD02929308 | NA19658.bam |
| SRR17634879 | SRP355248 | PRJNA792997 | SAMN24495273 | FD02929203 | NA19682.bam |
| SRR17634878 | SRP355248 | PRJNA792997 | SAMN24495274 | FD02929157 | NA19700.bam |
| SRR17634898 | SRP355248 | PRJNA792997 | SAMN24495275 | FD02929159 | NA19701.bam |
| SRR17634897 | SRP355248 | PRJNA792997 | SAMN24495276 | FD02929162 | NA19704.bam |
| SRR17634896 | SRP355248 | PRJNA792997 | SAMN24495277 | FD02929178 | NA19713.bam |
| SRR17634895 | SRP355248 | PRJNA792997 | SAMN24495278 | FD01114859 | NA19764.bam |
| SRR17634894 | SRP355248 | PRJNA792997 | SAMN24495279 | FD02929296 | NA19780.bam |
| SRR17634893 | SRP355248 | PRJNA792997 | SAMN24495280 | FD02929283 | NA19789.bam |
| SRR17634892 | SRP355248 | PRJNA792997 | SAMN24495281 | FD02929284 | NA19795.bam |
| SRR17634891 | SRP355248 | PRJNA792997 | SAMN24495282 | FD02929226 | NA19834.bam |
| SRR17634888 | SRP355248 | PRJNA792997 | SAMN24495283 | FD02929138 | NA19835.bam |
| SRR17634887 | SRP355248 | PRJNA792997 | SAMN24495284 | FD02929181 | NA19909.bam |
| SRR17634886 | SRP355248 | PRJNA792997 | SAMN24495285 | FD02929184 | NA19921.bam |
| SRR17634885 | SRP355248 | PRJNA792997 | SAMN24495286 | FD02929281 | NA19984.bam |
| SRR17634884 | SRP355248 | PRJNA792997 | SAMN24495287 | FD01115304 | NA20126.bam |
| SRR17634883 | SRP355248 | PRJNA792997 | SAMN24495288 | FD02929163 | NA20274.bam |
| SRR17634882 | SRP355248 | PRJNA792997 | SAMN24495289 | FD02929319 | NA20276.bam |
| SRR17634881 | SRP355248 | PRJNA792997 | SAMN24495290 | FD02929302 | NA20278.bam |
| SRR17634877 | SRP355248 | PRJNA792997 | SAMN24495291 | FD02929147 | NA20287.bam |
| SRR17634876 | SRP355248 | PRJNA792997 | SAMN24495292 | FD01115320 | NA20289.bam |
| SRR17635051 | SRP355248 | PRJNA792997 | SAMN24495293 | FD02929320 | NA20291.bam |
| SRR17634875 | SRP355248 | PRJNA792997 | SAMN24495294 | FD02929177 | NA20294.bam |
| SRR17634874 | SRP355248 | PRJNA792997 | SAMN24495295 | FD02929307 | NA20296.bam |
| SRR17634873 | SRP355248 | PRJNA792997 | SAMN24495296 | FD01115303 | NA20299.bam |
| SRR17634872 | SRP355248 | PRJNA792997 | SAMN24495297 | FD02929126 | NA20320.bam |
| SRR17635068 | SRP355248 | PRJNA792997 | SAMN24495298 | FD02929276 | NA20321.bam |
| SRR17635067 | SRP355248 | PRJNA792997 | SAMN24495299 | FD02929161 | NA20332.bam |
| SRR17635066 | SRP355248 | PRJNA792997 | SAMN24495300 | FD02929158 | NA20339.bam |
| SRR17635065 | SRP355248 | PRJNA792997 | SAMN24495301 | FD02929171 | NA20342.bam |
| SRR17635064 | SRP355248 | PRJNA792997 | SAMN24495302 | FD02929173 | NA20346.bam |
| SRR17635062 | SRP355248 | PRJNA792997 | SAMN24495303 | FD02929167 | NA20348.bam |
| SRR17635061 | SRP355248 | PRJNA792997 | SAMN24495304 | FD02929166 | NA20351.bam |
| SRR17635060 | SRP355248 | PRJNA792997 | SAMN24495305 | FD02929155 | NA20355.bam |
| SRR17635059 | SRP355248 | PRJNA792997 | SAMN24495306 | FD02929170 | NA20356.bam |
| SRR17635058 | SRP355248 | PRJNA792997 | SAMN24495307 | FD02929174 | NA20412.bam |
| SRR17635057 | SRP355248 | PRJNA792997 | SAMN24495308 | FD02929286 | NA20502.bam |
| SRR17635056 | SRP355248 | PRJNA792997 | SAMN24495309 | FD01115344 | NA20509.bam |
| SRR17635055 | SRP355248 | PRJNA792997 | SAMN24495310 | FD02929168 | NA20510.bam |
| SRR17635054 | SRP355248 | PRJNA792997 | SAMN24495311 | FD02929128 | NA20511.bam |
| SRR17635053 | SRP355248 | PRJNA792997 | SAMN24495312 | FD02929148 | NA20513.bam |
| SRR17634998 | SRP355248 | PRJNA792997 | SAMN24495313 | FD02929318 | NA20515.bam |
| SRR17634997 | SRP355248 | PRJNA792997 | SAMN24495314 | FD01114858 | NA20516.bam |
| SRR17635050 | SRP355248 | PRJNA792997 | SAMN24495315 | FD02929145 | NA20519.bam |
| SRR17635049 | SRP355248 | PRJNA792997 | SAMN24495316 | FD02929165 | NA20520.bam |
| SRR17635048 | SRP355248 | PRJNA792997 | SAMN24495317 | FD02929146 | NA20524.bam |
| SRR17635047 | SRP355248 | PRJNA792997 | SAMN24495318 | FD02929143 | NA20525.bam |
| SRR17635046 | SRP355248 | PRJNA792997 | SAMN24495319 | FD02929154 | NA20532.bam |
| SRR17635045 | SRP355248 | PRJNA792997 | SAMN24495320 | FD02929156 | NA20763.bam |
| SRR17635044 | SRP355248 | PRJNA792997 | SAMN24495321 | FD02929211 | NA20767.bam |
| SRR17635043 | SRP355248 | PRJNA792997 | SAMN24495322 | FD01115360 | NA20812.bam |
| SRR17635041 | SRP355248 | PRJNA792997 | SAMN24495323 | FD02929153 | NA20814.bam |
| SRR17635040 | SRP355248 | PRJNA792997 | SAMN24495324 | FD01115296 | NA20822.bam |
| SRR17635039 | SRP355248 | PRJNA792997 | SAMN24495325 | FD02929233 | NA20845.bam |
| SRR17635038 | SRP355248 | PRJNA792997 | SAMN24495326 | FD02929133 | NA20846.bam |
| SRR17635037 | SRP355248 | PRJNA792997 | SAMN24495327 | FD02929206 | NA20863.bam |
| SRR17635004 | SRP355248 | PRJNA792997 | SAMN24495328 | FD02929152 | NA20864.bam |
| SRR17635003 | SRP355248 | PRJNA792997 | SAMN24495329 | FD02929235 | NA20867.bam |
| SRR17635002 | SRP355248 | PRJNA792997 | SAMN24495330 | FD02929225 | NA20887.bam |
| SRR17635001 | SRP355248 | PRJNA792997 | SAMN24495331 | FD02929314 | NA20889.bam |
| SRR17635000 | SRP355248 | PRJNA792997 | SAMN24495332 | FD02929253 | NA20890.bam |
| SRR17634977 | SRP355248 | PRJNA792997 | SAMN24495333 | FD02929252 | NA20901.bam |
| SRR17634976 | SRP355248 | PRJNA792997 | SAMN24495334 | FD02929248 | NA20904.bam |
| SRR17634996 | SRP355248 | PRJNA792997 | SAMN24495335 | FD02929200 | NA21090.bam |
| SRR17634995 | SRP355248 | PRJNA792997 | SAMN24495336 | FD02929185 | NA21093.bam |
| SRR17634994 | SRP355248 | PRJNA792997 | SAMN24495337 | FD02929214 | NA21105.bam |
| SRR17634993 | SRP355248 | PRJNA792997 | SAMN24495338 | FD02929180 | NA21118.bam |
| SRR17634992 | SRP355248 | PRJNA792997 | SAMN24495339 | FD02929183 | NA21128.bam |
| SRR17634991 | SRP355248 | PRJNA792997 | SAMN24495340 | FD02929182 | NA21135.bam |
| SRR17634990 | SRP355248 | PRJNA792997 | SAMN24495341 | FD01114846 | NA24143.bam |
| SRR17634989 | SRP355248 | PRJNA792997 | SAMN24495342 | FD01114845 | NA24149.bam |
| SRR17634987 | SRP355248 | PRJNA792997 | SAMN24495343 | FD01114844 | NA24385.bam |
| SRR18019669 | SRP355248 | PRJNA792997 | SAMN24495089 | FD01114902 | HG00111.bam |

References

1. Biesecker LG, Mullikin JC, Facio FM, Turner C, Cherukuri PF, Blakesley RW, et al. The ClinSeq Project: piloting large-scale genome sequencing for research in genomic medicine. Genome Res. 2009;19(9):1665-74.

2. Genomes Project C, Abecasis GR, Altshuler D, Auton A, Brooks LD, Durbin RM, et al. A map of human genome variation from population-scale sequencing. Nature. 2010;467(7319):1061-73.

3. Genomes Project C, Abecasis GR, Auton A, Brooks LD, DePristo MA, Durbin RM, et al. An integrated map of genetic variation from 1,092 human genomes. Nature. 2012;491(7422):56-65.

4. Genomes Project C, Auton A, Brooks LD, Durbin RM, Garrison EP, Kang HM, et al. A global reference for human genetic variation. Nature. 2015;526(7571):68-74.

5. Lee S, Abecasis GR, Boehnke M, Lin X. Rare-variant association analysis: study designs and statistical tests. Am J Hum Genet. 2014;95(1):5-23.

6. Reich D, Price AL, Patterson N. Principal component analysis of genetic data. Nature Genetics. 2008;40(5):491-2.

7. Jun G, Flickinger M, Hetrick KN, Romm JM, Doheny KF, Abecasis GR, et al. Detecting and estimating contamination of human DNA samples in sequencing and array-based genotype data. Am J Hum Genet. 2012;91(5):839-48.

8. Zook JM, Chapman B, Wang J, Mittelman D, Hofmann O, Hide W, et al. Integrating human sequence data sets provides a resource of benchmark SNP and indel genotype calls. Nature Biotechnology. 2014;32(3):246-51.

9. Belsare S, Levy-Sakin M, Mostovoy Y, Durinck S, Chaudhuri S, Xiao M, et al. Evaluating the quality of the 1000 genomes project data. BMC Genomics. 2019;20(1):620.

10. Wall JD, Tang LF, Zerbe B, Kvale MN, Kwok P-Y, Schaefer C, et al. Estimating genotype error rates from high-coverage next-generation sequence data. Genome Res. 2014;24(11):1734-9.

1. *We extended the 7. Jun G, Flickinger M, Hetrick KN, Romm JM, Doheny KF, Abecasis GR, et al. Detecting and estimating contamination of human DNA samples in sequencing and array-based genotype data. Am J Hum Genet. 2012;91(5):839-48. methodology not only to include the sites that are called as homozygous, but also to include sites that have missing genotypes. The purpose of including genotype sites that are missed by the Illumina genotyping software. This effect is due to ambiguous signal intensity to confidently call one of three possible genotypes (AA, or AB, or BB). [↑](#footnote-ref-1)
